# Supplementary material for: Scalable generation of hematopoietic stem cell-engineered off-the-shelf mono-specific cytotoxic T cells targeting solid tumors
Source: Cell Rep Med. 2026 Aug 19;7(9):102998. doi: 10.1016/j.xcrm.2026.102998 (PMC13589493; doi:10.1016/j.xcrm.2026.102998)
Supplement: Supplementary file 1 [file mmc1.pdf]

Cell Reports Medicine, Volume *7*

Supplemental information

Scalable generation of hematopoietic stem cell-engineered off-the-shelf mono-specific cytotoxic T cells targeting solid tumors

Yichen Zhu, Jiaji Yu, Yu Jeong Kim, Yanxin Tian, Zhe Li, Yuning Chen, Zibai Lyu, Enbo Zhu, Annabel S. Zhao, Nathan Ma, Catherine Zhang, Adam Kramer, Matthew Wilson, Ryan Hon, Yu-Chen Wang, Siyu Lin, Xinyuan Shen, Zoe Hahn, Yuchong Zhang, Aijun Wang, Yan-Ruide Li, and Lili Yang

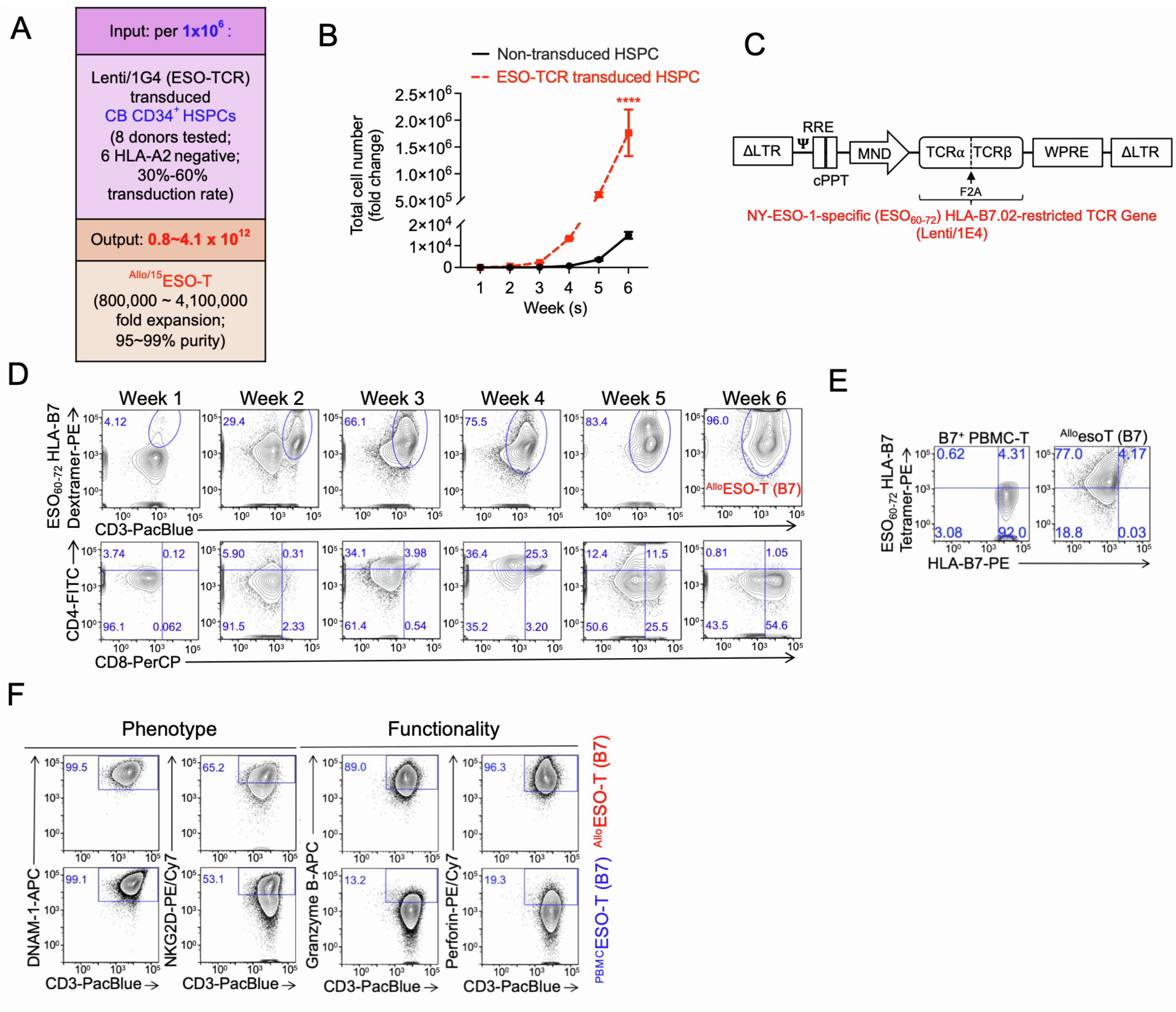

Figure S1: Generation and characterization of allogeneic NY-ESO-1 targeting mono-specific cytotoxic T (^{Allo}ESO-T) cells; related to Figure 1.

(A) Yield of $^{Allo/15}$ESO-T cells from 8 different CB donors.

(B) Growth curve of HSPCs either non-transduced or transduced with exogenous ESO-TCR in the culture (n = 3).

(C) Schematics showing the design of the lentiviral vector carrying HLA-B7.02 restricted, NY-ESO-$1_{60\text{-}72}$-specifc TCR.

(D) FACS monitoring of the generation of ^{Allo}ESO-T (B7) cells (identified as $ESO_{60\text{-}72}$ HLA-B7 Dextramer$^+$CD3$^+$ cells) over the 6-week *ex vivo* HSPC-ESO-T cell culture.

(E) FACS analyses showing the generation of ^{Allo}ESO-T (B7) is independent of matching MHC expression for positive selection.

(F) FACS detection of surface markers and cytotoxic molecules of ^{Allo}ESO-T (B7) cells.

Representative of 3 independent biological replicates. Data are presented as the mean ± SEM. ns, not significant, *p<0.05, ****p<0.0001 by Student's *t* test (B).

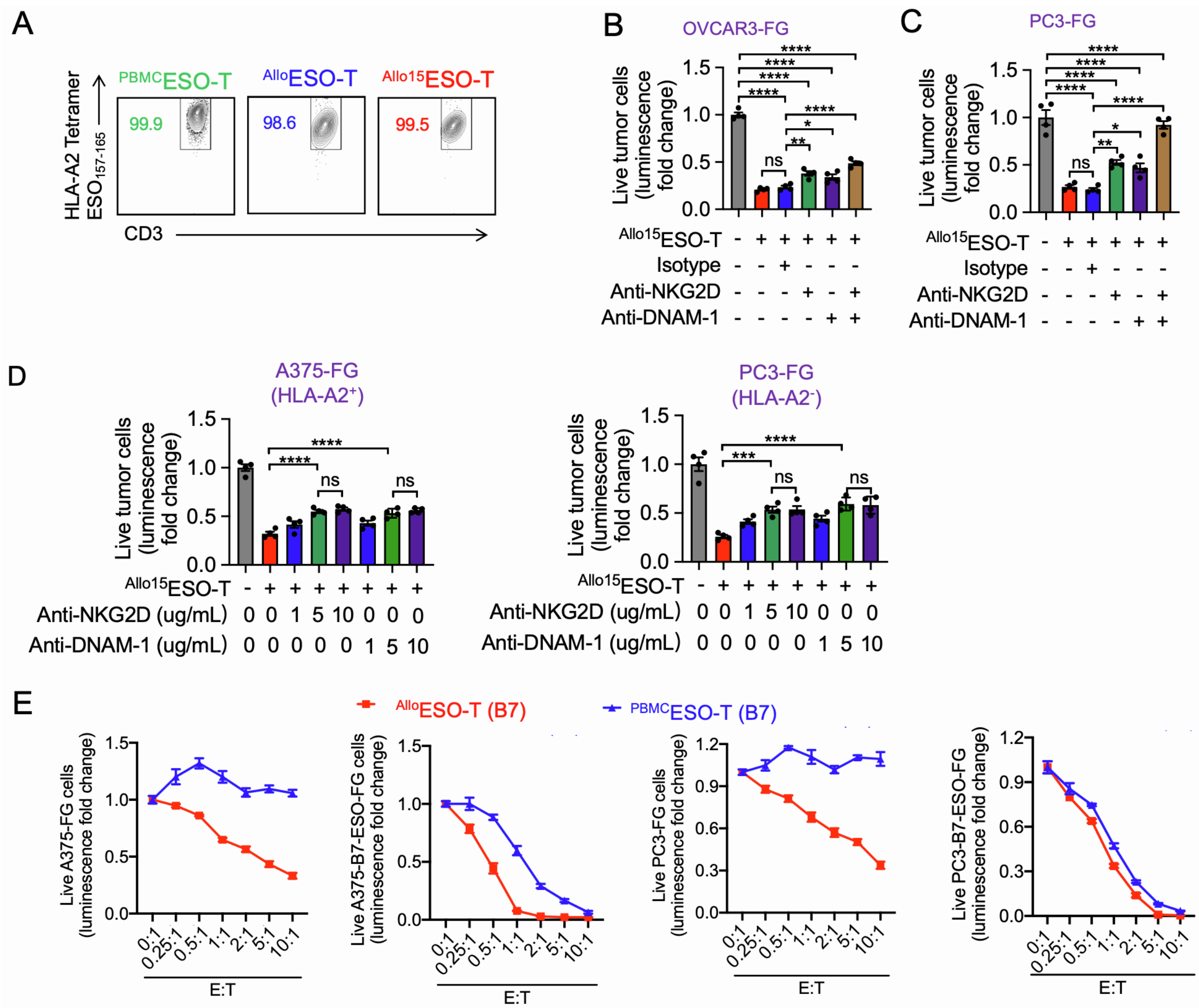

Figure S2: *In vitro* antitumor efficacy and mechanism of action (MOA) study of $^{Allo/15}$ESO-T cells; related to Figure 2.

(A) NY-ESO-1 tetramer staining of ^{Allo15}ESO-T, ^{Allo}ESO-T, and ^{PBMC}ESO-T cell products.

(B-D) Studying the tumor cell killing mechanisms of ^{Allo15}ESO-T cells mediated by NKG2D and DNAM-1. (B) OVCAR3-FG tumor cell killing data at 24 h (E:T ratio = 0.5:1; n = 4). (C) PC3-FG tumor cell killing data at 24 h (E:T ratio = 5:1; n = 4). (D) Dose–response titration study of NK receptor–blocking antibodies. Tumor cell killing at 24 h at indicated concentrations.

(E) *In vitro* tumor cell killing data at 24 h by ^{Allo}ESO-T (B7) (n = 4). ^{PBMC}ESO-T (B7) cells were included as a therapeutic cell control.

Representative of 3 experiments. Data are presented as the mean ± SEM. ns, not significant, *p<0.05, **p<0.01, ***p<0.001, ****p<0.0001 by one-way ANOVA (A-D).

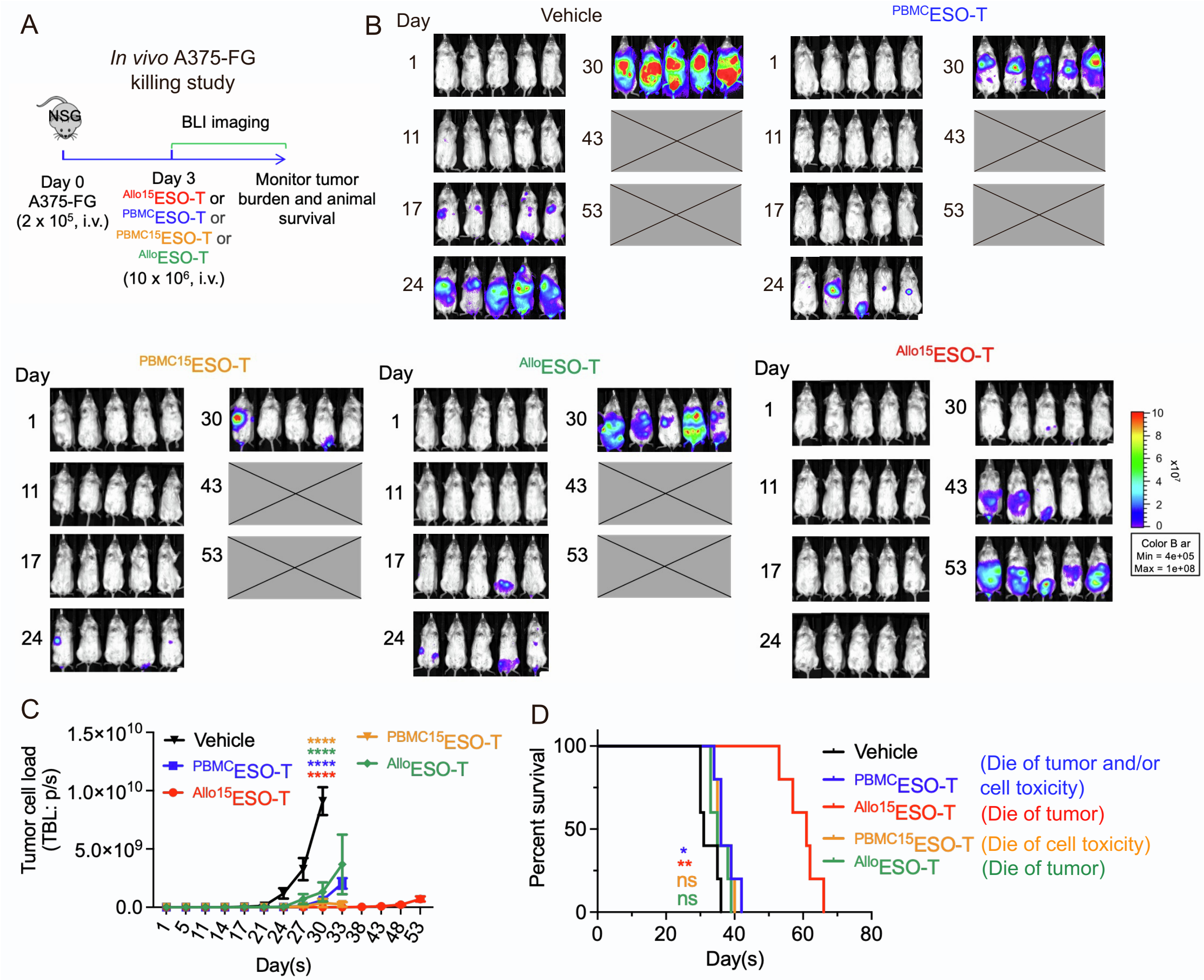

Figure S3: *In vivo* antitumor efficacy study of Allo/15ESO-T cells in a metastatic melanoma tumor model. related to Figure 4.

(A) Experimental design. BLI, live animal bioluminescence imaging. (B) BLI images measuring tumor loads in experimental mice over time. (C) Quantification of c (n = 5). (D) Kaplan–Meier survival curves (n = 5).

Note that the data for the Vehicle, Allo15ESO-T, and PBMCESO-T groups were also presented in the main Figs. 4A-4E.

Representative of 2 independent biological replicates. Data are presented as the mean ± SEM. ****p<0.0001 by one-way ANOVA (C)

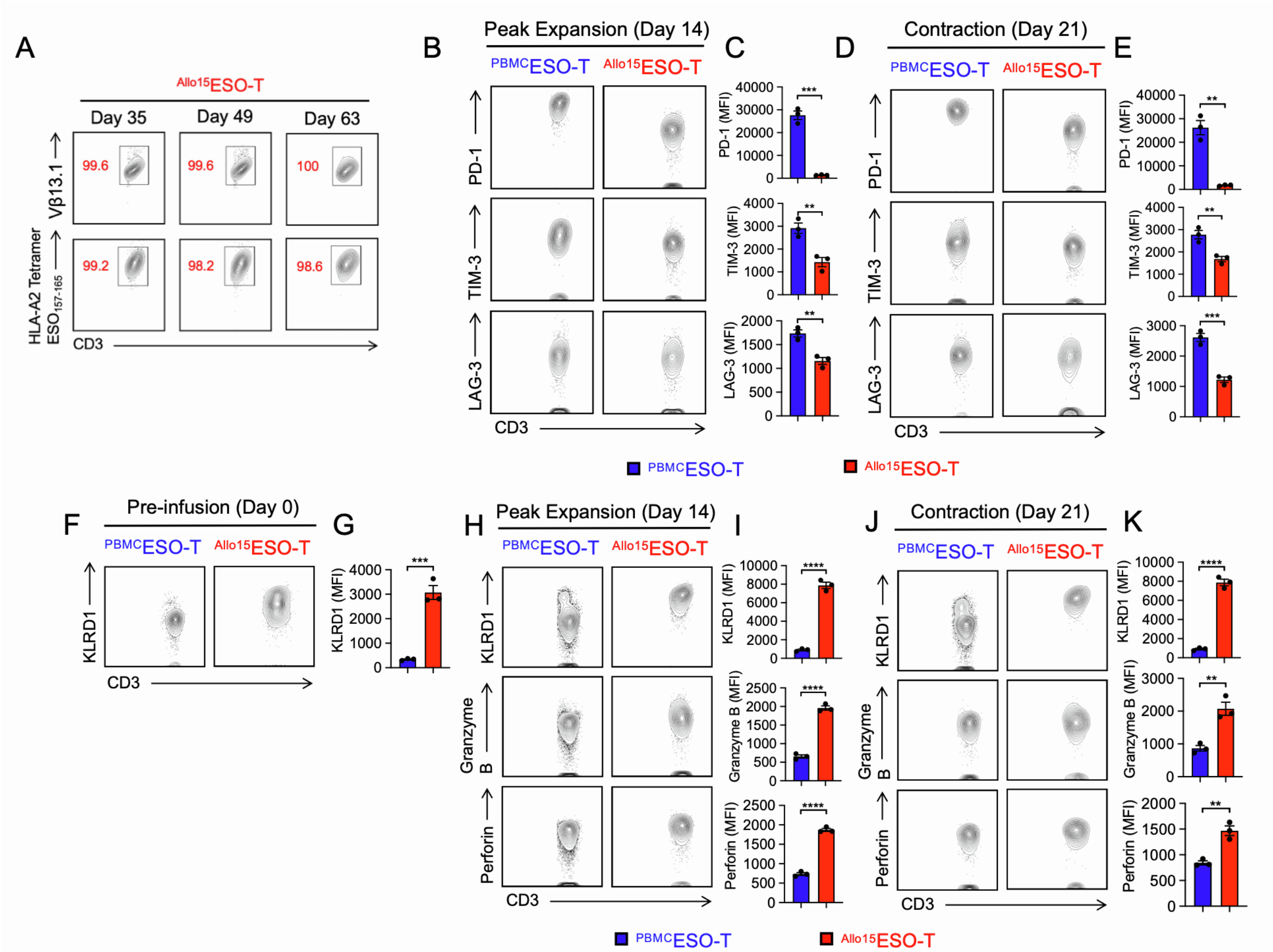

Figure S4: ***In vivo*** **profiling of** ^{Allo15}**ESO-T cells in a metastatic melanoma tumor model. related to Figures 3 and 5.**

(A) Transgenic TCR and NY-ESO-1 tetramer staining of ^{Allo15}ESO-T cells *in vivo* at multiple time points.

(B, D, F, H, and J) FACS detection of TEMRA and exhaustion marker expression *in vivo*. (C, E, G, I, and K) Quantification of (B, D, F, H, and J) (n = 3).

Representative of 3 independent biological replicates. Data are presented as the mean ± SEM. $^{**}p<0.01$, $^{***}p<0.001$, $^{****}p<0.0001$ by Student's *t* test (C, E, G, I, and K)

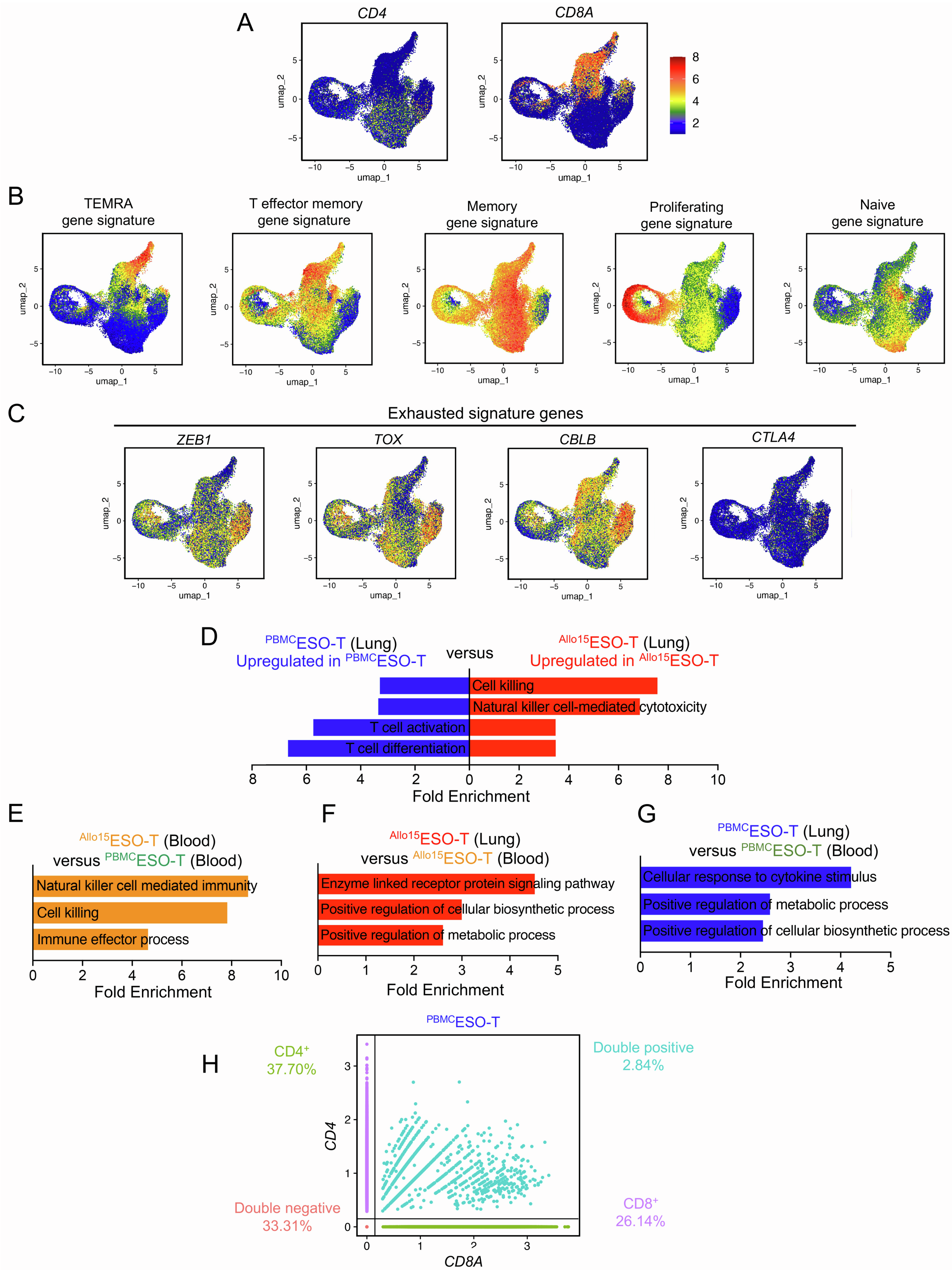

Figure S5: *In vivo* gene profiling study of ^{Allo15}ESO-T cells; related to Figure 5.
(A) UMAP plots showing the expression of *CD4* and *CD8A*.

(B) UMAP plots showing the expression distribution of TEMRA, T effector memory, memory, proliferating, naïve gene signatures.
(C) UMAP plots showing the expression of the exhausted signature genes *ZEB1*, *TOX*, *CBLB* and *CTLA4*.
(D-E) Pathway analyses of differentially expressed genes comparing lung-derived (D) and periphery blood-derived (E) ^{Allo15}ESO-T cells with ^{PBMC}ESO-T cells.
(F-G) Pathway analysis of differentially expressed genes comparing lung- versus blood-derived ^{Allo15}ESO-T cells (f) and lung- versus blood-derived ^{PBMC}ESO-T cells (G).
(H) Scatter plot showing single-cell expression levels of *CD4* (y-axis) and *CD8A* (x-axis) in ^{PBMC}ESO-T cells. Cells were classified into four populations: $CD4^+$ (green, 37.70%), $CD8^+$ (purple, 26.14%), double positive (cyan, 2.84%), and double negative (red, 33.31%), based on thresholded expression of *CD4* and *CD8A* transcripts.

The experiment was performed once; cells isolated from five mice of each experimental group were combined for analyses.

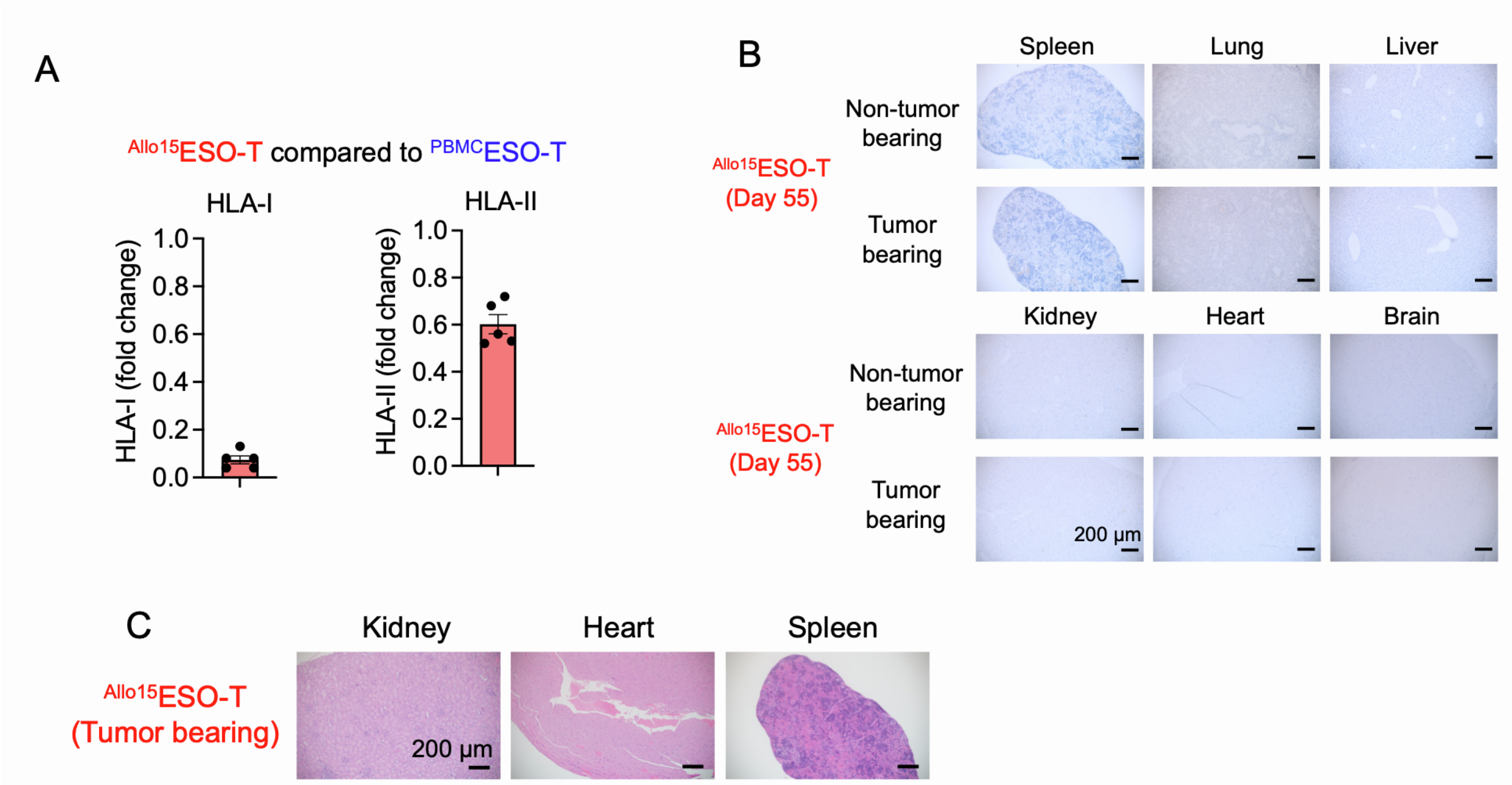

D

Group	Mouse ID	Pathologist's score																										
		Bone marrow			Spleen			Ileum			Pancreas			Kidney			Lung			Liver			Heart			Brain		
		Inf	HN	NHN	Inf	HN	NHN	Inf	HN	NHN	Inf	HN	NHN	Inf	HN	NHN	Inf	HN	NHN	Inf	HN	NHN	Inf	HN	NHN	Inf	HN	NHN
Allo15ESO-T	1	0	0	0	0	0	0	1	0	0	0	0	0	0	0	0	0	0	0	0	0	0	0	0	0	0	0	0
	2	0	0	0	0	0	0	0	0	0	0	0	0	0	0	0	0	0	0	0	0	0	0	0	0	0	0	0
	3	0	0	0	1	0	0	0	0	0	0	0	0	0	0	0	0	0	0	1	0	0	0	0	0	0	0	0
	4	0	0	0	0	0	0	0	0	0	0	0	0	0	0	0	1	0	0	0	0	0	0	0	0	0	0	0
	5	0	0	0	0	0	0	0	0	0	0	0	0	0	0	0	0	0	0	0	0	0	0	0	0	0	0	0
Blank NSG	6	0	0	0	0	0	0	0	0	0	0	0	0	0	0	0	0	0	0	0	0	0	0	0	0	0	0	0
	7	0	0	0	0	0	0	0	0	0	0	0	0	0	0	0	0	0	0	0	0	0	0	0	0	0	0	0
	8	0	0	0	0	0	0	0	0	0	0	0	0	0	0	0	0	0	0	0	0	0	0	0	0	0	0	0
	9	0	0	0	0	0	0	1	0	0	0	0	0	0	0	0	0	0	0	0	0	0	0	0	0	0	0	0
	10	0	0	0	0	0	0	0	0	0	0	0	0	0	0	0	0	0	0	1	0	0	0	0	0	0	0	0

Pathologist's score: 0 No abnormal findings; 1 Mild; 2 Moderate; 3 Severe

LEGEND: Inf: Inflammation; HN: Hematopoietic Neoplasm; NHN: Non-Hematopoietic Neoplasm

Figure S6. Safety and immunogenicity study of Allo15ESO-T cells; related to Figure 7.
(A) Fold change of HLA-I/II expression between Allo15ESO-T cells and PBMCESO-T cells.
(B) Immunohistochemical staining of human CD3 in major organs at day 55 post-infusion. Scale bar, 200 μm.
(C) Hematoxylin and eosin-stained tissue sections. Tissues were collected on day 55.
(D) Studying the long-term safety of Allo15ESO-T cells. Tissues from experimental mice were collected 55 days after injection with Allo15ESO-T cells. Data were presented as pathologist's scores of individual mouse tissues (n = 5).

Representative of 3 biological replicates. Data are presented as the mean ± SEM. ns, not significant by one-way ANOVA (B).

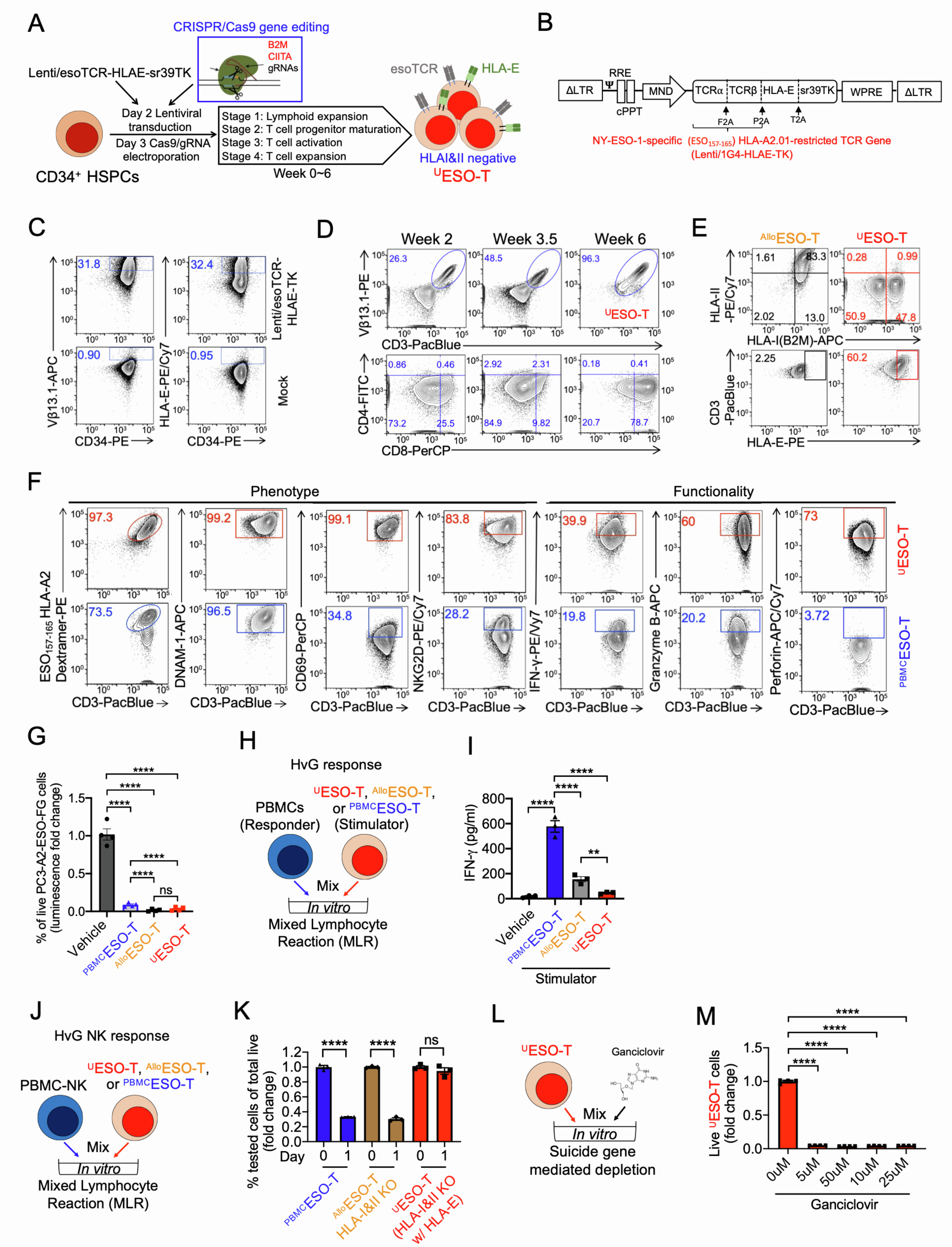

Figure S7: Development of allogeneic HLA-ablated universal T (^{U}ESO-T) cells.

(A) Schematics showing the generation of ^{U}ESO-T cells.
(B) Schematics showing the design of design of the lentiviral vector encoding a pair of ESO TCR α and β chains, an HLA-E, and a sr39TK suicide gene.
(C) Intracellular expression of ESO TCR and HLA-E in $CD34^{+}$ HSPCs at 72 h after lentivector transduction.
(D) FACS monitoring of the generation of ^{U}ESO-T cells over the 6-week *ex vivo* HSPC-ESO-T cell culture.
(E) FACS detection of HLA-I/II and HLA-E expressions in ^{U}ESO-T cells.
(F) FACS detection of surface markers, intracellular cytokines and cytotoxic molecules in ^{U}ESO-T cells. ^{Allo}ESO-T cells were included for comparison.
(G) Tumor cell killing data by ^{U}ESO-T cells at 24 h.
(H-I) Studying the T cell-mediated allorejection of ^{U}ESO-T cells. (H) Experimental design. (I) ELISA analyses of IFNγ production on day 4 ($n = 4$).
(J-K) Studying the NK cell-mediated allorejection of ^{U}ESO-T cells. (J) Experimental design. (K) FACS analyses of the indicated live cells ($n = 3$).
(L-M) Studying the functional activity of the suicide gene sr39TK as an additional safety mechanism in ^{U}ESO-T cells. (L) Experimental design. ^{U}ESO-T cells were cultured in the presence of gradient concentrations of ganciclovir for 3 days. (M) FACS analyses of the indicated live cells on day 3 (n=4).

Representative of 3 independent biological replicates. Data are presented as the mean ± SEM. ns, not significant, $^{**}p < 0.01$, $^{****}p < 0.0001$, by one-way ANOVA (G, I, K, M).

Table S1. Cord blood donor information; related to Figure 1.

CB donors	Gender	Race	ABO/Rh
1	Male	Caucasian	O-
2	Female	Caucasian	A+
3	Female	Hispanic	O-
4	Male	Black	A-
5	Female	Hispanic	A+
6	Male	Asian	B+
7	Male	Caucasian	B-
8	Female	Unknown	O+
