# Supplementary material for: Scalable generation of hematopoietic stem cell-engineered off-the-shelf mono-specific cytotoxic T cells targeting solid tumors
Source: Cell Rep Med. 2026 Aug 19;7(9):102998. doi: 10.1016/j.xcrm.2026.102998 (PMC13589493; doi:10.1016/j.xcrm.2026.102998)
Supplement: Supplementary file 2 [file mmc2.pdf]

Cell Reports Medicine

Article

Scalable generation of hematopoietic stem cell-engineered off-the-shelf mono-specific cytotoxic T cells targeting solid tumors

Graphical abstract

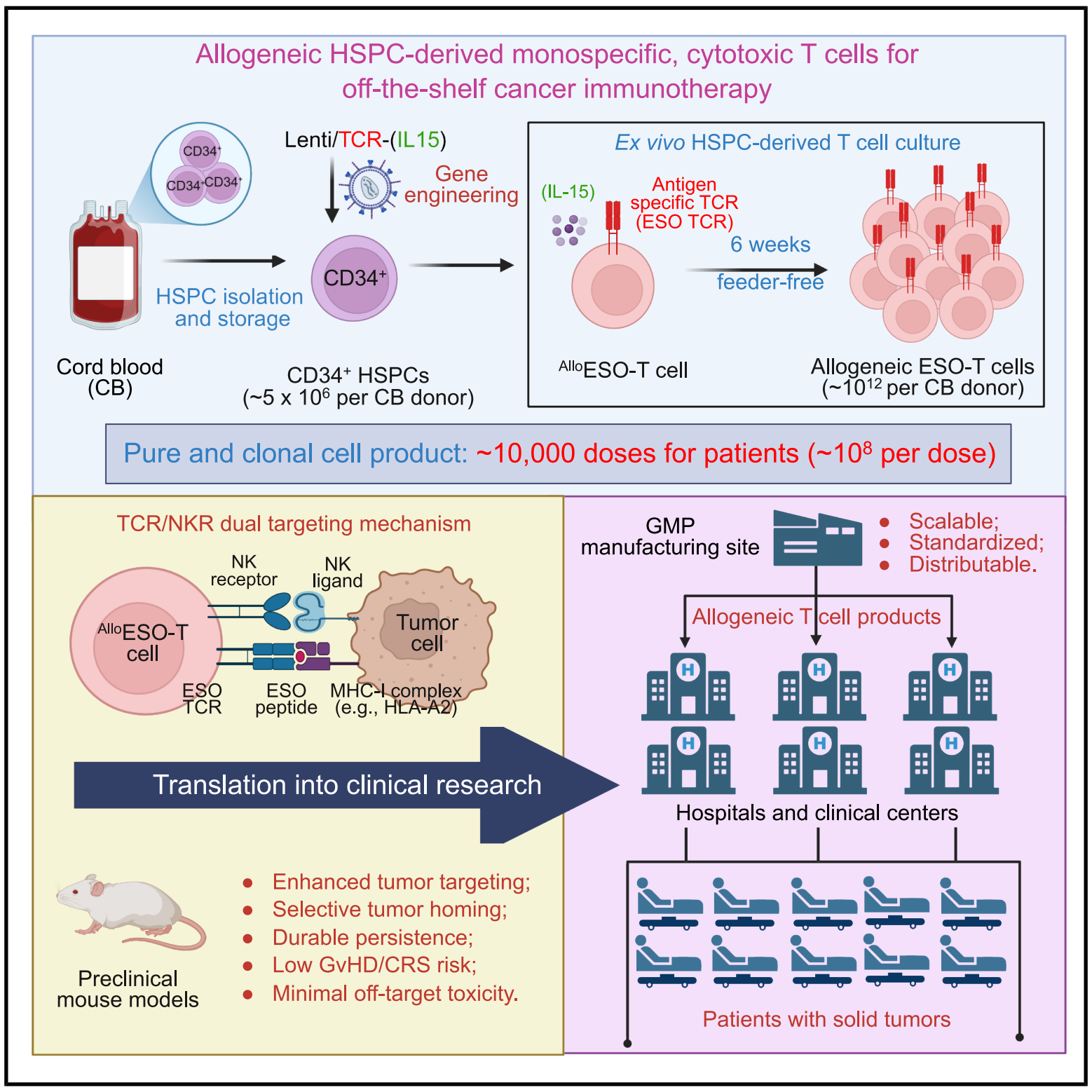

Highlights

- HSPC-derived AlloESO-T cells enable scalable, feeder-free T cell manufacturing
- AlloESO-T cells overcome autologous complexity, HLA restriction, and donor variability
- AlloESO-T cells display tumor homing, durable persistence, and TCR/NKR dual targeting
- AlloESO-T cells show minimal GvHD/CRS risk and stable hypoimmunogenicity

Authors

Yichen Zhu, Jiaji Yu, Yu Jeong Kim, ..., Aijun Wang, Yan-Ruide Li, Lili Yang

Correspondence

charlie.li@ucla.edu (Y.-R.L.),
liliyang@ucla.edu (L.Y.)

In brief

Zhu et al. develop a scalable, feeder-free platform to generate allogeneic NY-ESO-1-specific T (AlloESO-T) cells from human HSPCs. These mono-specific, cytotoxic T cells combine TCR-driven antigen specificity with NK-like cytotoxicity, achieving enhanced tumor targeting, durable persistence, and reduced CRS/GvHD risk, which support their potential as an off-the-shelf therapy for solid tumors.

Zhu et al., 2026, Cell Reports Medicine 7, 102998
September 15, 2026 © 2026 The Author(s). Published by Elsevier Inc.
https://doi.org/10.1016/j.xcrm.2026.102998

Cell Reports Medicine

CellPress OPEN ACCESS

Article

Scalable generation of hematopoietic stem cell-engineered off-the-shelf mono-specific cytotoxic T cells targeting solid tumors

Yichen Zhu,[1,2,10] Jiaji Yu,[1,2,10] Yu Jeong Kim,[1,2] Yanxin Tian,[1,2] Zhe Li,[1,2] Yuning Chen,[1,2] Zibai Lyu,[1,2] Enbo Zhu,[1,2] Annabel S. Zhao,[1,2] Nathan Ma,[1,2] Catherine Zhang,[1,2] Adam Kramer,[1,2] Matthew Wilson,[1,2] Ryan Hon,[1,2] Yu-Chen Wang,[3] Siyu Lin,[4] Xinyuan Shen,[1,2] Zoe Hahn,[1,2] Yuchong Zhang,[1,2] Aijun Wang,[4] Yan-Ruide Li,[1,2,*] and Lili Yang[1,2,5,6,7,8,9,11,*]

[1]Department of Microbiology, Immunology & Molecular Genetics, University of California, Los Angeles (UCLA), Los Angeles, CA 90095, USA
[2]Department of Bioengineering, UCLA, Los Angeles, CA 90095, USA
[3]Department of Medicine, Division of Cardiology, UCLA, Los Angeles, CA 90095, USA
[4]Department of Biomedical Engineering, University of California, Davis, Davis, CA 95616, USA
[5]Eli and Edythe Broad Centre of Regenerative Medicine and Stem Cell Research, UCLA, Los Angeles, CA 90095, USA
[6]Jonsson Comprehensive Cancer Center, UCLA, Los Angeles, CA 90095, USA
[7]Molecular Biology Institute, UCLA, Los Angeles, CA 90095, USA
[8]Parker Institute for Cancer Immunotherapy, UCLA, Los Angeles, CA 90095, USA
[9]Goodman-Luskin Microbiome Center, UCLA, Los Angeles, CA 90095, USA
[10]These authors contributed equally
[11]Lead contact
*Correspondence: charlie.li@ucla.edu (Y.-R.L.), liliyang@ucla.edu (L.Y.)
https://doi.org/10.1016/j.xcrm.2026.102998

SUMMARY

Adoptive T cell therapy for solid tumors is limited by autologous manufacturing complexity and, in allogeneic settings, risks including graft-versus-host disease (GvHD), HLA restriction, and donor variability. We develop a scalable, feeder-free platform to differentiate gene-engineered hematopoietic stem and progenitor cells (HSPCs) into allogeneic, NY-ESO-1-specific cytotoxic T (^{Allo}ESO-T) cells. Product phenotype, function, tumor homing, and safety are assessed against solid tumor models and benchmarked to peripheral blood mononuclear cell (PBMC)-derived TCR-engineered T cells. ^{Allo}ESO-T cells display a uniform cytotoxic phenotype, with dual tumor targeting through a transgenic TCR and natural killer receptors. Relative to PBMC-derived counterparts, ^{Allo}ESO-T cells show superior cytotoxicity, selective solid-tumor homing, durable killing persistence, and resilience to immune evasion. They also maintain low GvHD and cytokine release syndrome risk, while retaining stable hypoimmunogenic features. These findings establish HSPC-derived ^{Allo}ESO-T cells as an off-the-shelf, mono-specific cytotoxic T cell therapy with scalable manufacturing, enhanced efficacy, and improved safety, which support broad applicability of ^{Allo}ESO-T cells across solid tumors.

INTRODUCTION

Adoptive cell therapy (ACT) using cytotoxic T cells has emerged as a transformative strategy for targeting tumors with antigen-specific precision.[1–3] Among the various ACT modalities, T cell receptor-engineered T (TCR-T) cells offer the unique ability to recognize intracellular tumor-associated antigens presented by human leukocyte antigen (HLA) molecules, expanding therapeutic opportunities beyond surface antigens.[4,5] A prominent class of such intracellular targets is cancer-testis antigens (CTAs), which are characterized by high immunogenicity, consistent expression in a variety of cancers, and restricted expression in immune-privileged tissues.[6–8] The recent FDA approval of afamitresgene autoleucel (Tecelra)—a TCR-T therapy targeting the CTA MAGE-A4—for the treatment of unresectable or metastatic synovial sarcoma—marks a major milestone in the field, representing the first regulatory approval of a genetically modified T cell therapy for a solid tumor.[9] Another extensively studied CTA is NY-ESO-1, which has emerged as a prototypical target for TCR-T therapy.[10] Encoded by *CTAG1B*, NY-ESO-1 is aberrantly expressed in a wide spectrum of malignancies—including melanoma, ovarian cancer, lung cancer, and synovial sarcoma—while remaining largely absent from normal somatic tissues.[11–13] Importantly, NY-ESO-1 is highly immunogenic and capable of eliciting robust T cell responses across multiple HLA alleles.[14–16] Multiple early phase clinical trials have demonstrated the therapeutic potential of NY-ESO-1-specific TCR-T cells.[10,17–21] NY-ESO-1-specific TCRs produced objective responses in patients with melanoma (45%) and synovial sarcoma (67%).[22]

Despite recent clinical progress, the broad application of TCR-T cell therapy in solid tumors remains constrained by multiple barriers.[23,24] These include inefficient trafficking of transferred T cells to tumor sites, functional impairment within the immunosuppressive tumor microenvironment (TME), tumor

© 2026 The Author(s). Published by Elsevier Inc.
This is an open access article under the CC BY license (http://creativecommons.org/licenses/by/4.0/).

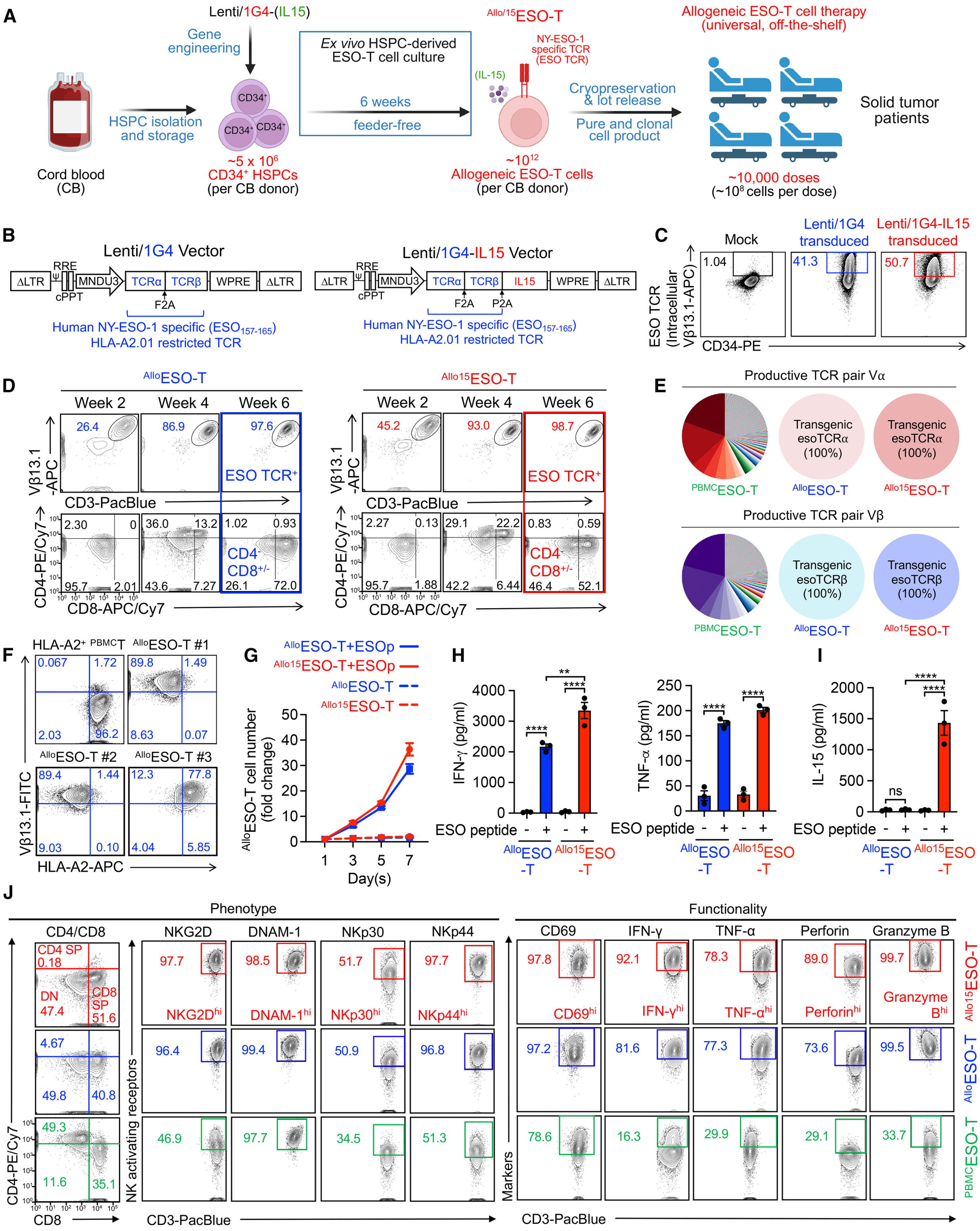

Figure 1. Generation and characterization of $^{Allo/15}$ESO-T cells

(A) Schematics showing $^{Allo/15}$ESO-T cell generation. CB, cord blood; Lenti/1G4-(IL-15), lentiviral vector encoding ESO TCR α and β chains, and/or an additional human soluble interleukin 15 (IL-15). HLA-A2.01 restricted, NY-ESO-1$_{157–165}$-specific TCR is denoted as 1G4.

(legend continued on next page)

antigen heterogeneity or loss that undermines durable responses,[25–29] the need for patient-specific leukapheresis, time-intensive and laborious manufacturing workflows, and considerable variability in T cell quality and yield across patient.[30] In contrast, allogeneic TCR-T approaches offer the promise of "off-the-shelf" availability but raise their own set of complex challenges.[31,32] A primary concern in the allogeneic setting is the risk of graft-versus-host disease (GvHD) and alloreactivity, which can result from the presence of endogenous polyclonal TCRs that could mispair with the introduced TCR chains and mediate recognition of mismatched host HLA molecules.[31,32] Overcoming these issues requires extensive gene editing to disrupt endogenous TCR expression and mitigate HLA mismatch, typically through multiplexed knockout of *TRAC* and *TRBC* loci and/or HLA class I components.[33] While such editing strategies can enhance safety, they introduce additional layers of genetic manipulation, increase manufacturing complexity, and raise regulatory concerns.[34–37] These limitations highlight the urgent need for next-generation TCR-T platforms that are not only potent and antigen-specific but also safe, mono-specific, and compatible with robust, scalable manufacturing.

Stem cells, including pluripotent stem cells (PSCs) and hematopoietic stem and progenitor cells (HSPCs), provide a versatile platform for genetic engineering and directed differentiation into functional immune effectors.[38–42] In this study, we developed a next-generation *in vitro* system for generating mono-specific cytotoxic T cells from HSPCs, engineered to express a high-affinity TCR targeting NY-ESO-1. We conducted a comprehensive preclinical evaluation of these engineered cells to evaluate their pharmacology, *in vitro* and *in vivo* efficacy, mechanism of action (MOA), pharmacokinetics/pharmacodynamics (PK-PD), safety, and immunogenicity, establishing the therapeutic potential of this stem cell-based platform for scalable and precise TCR-T cell therapy targeting solid tumors.

RESULTS

Scalable generation of HSPC-engineered off-the-shelf mono-specific cytotoxic NY-ESO-1-specific T cells

Cryopreserved $CD34^+$ HSPCs derived from human cord blood (e.g., from HemaCare) were utilized to generate T cell products, using a clinically guided differentiation protocol (Figure 1A).[38] Following thawing, $CD34^+$ HSPCs were transduced with lentiviral vectors co-expressing a TCR, along with additional genes (AGs), and then cultured for 48 h (Figures 1A and 1B). The TCR utilized in this study was specific to the NY-ESO-$1_{157–165}$/HLA-A2 complex (clone 1G4) and has been clinically employed.[17] Lentiviral transduction resulted in efficient gene delivery into HSPCs (Figure 1C). Human interleukin 15 (IL-15) was included as an optional AG in the lentivector to enhance the *in vivo* persistence and antitumor activity of the engineered T cell products.[38,43–48]

The gene-modified HSPCs were cultured for approximately 6 weeks to generate allogeneic, NY-ESO-1-specific, mono-specific cytotoxic T (^{Allo}ESO-T) cells and their IL-15-enhanced derivative (^{Allo15}ESO-T cells) (Figure 1A). The culture system was designed to be feeder free, aligning with the clinical and commercial manufacturing standards.[49] In addition, two alternative feeder-dependent strategies—using either $ESO_{157–165}$ peptide-pulsed healthy donor PBMC or K562-based artificial antigen-presenting cells (aAPCs)—were also compatible with clinical translation.[47,49] The resulting cell products demonstrated high yield and purity: from a single cord blood donor (containing $\sim$1–5 $\times$ 10^6 HSPCs), the culture generated approximately 10^{12} mature ^{Allo}ESO-T and ^{Allo15}ESO-T cells (denoted as $^{Allo/15}$ESO-T cells), potentially enabling the preparation of 1,000–10,000 therapeutic doses, assuming standard dosing of $\sim10^8$–10^9 cells per treatment (Figure 1A).

To determine whether exogenous TCR is required, $CD34^+$ HSPCs were differentiated under identical culture conditions in the presence or absence of ESO-TCR transduction. HSPCs transduced with the ESO-specific TCR underwent robust expansion and differentiation, whereas non-transduced HSPCs exhibited minimal proliferation and failed to generate a comparable T cell population (Figure S1B). These findings demonstrate that exogenous TCR expression is required for efficient generation and expansion of our ^{Allo}ESO-T cell product.

The *in vitro* differentiation process followed the canonical T cell developmental trajectory, progressing from $CD4^-CD8^-$ double-negative (DN) to $CD4^+CD8^+$ double-positive (DP), and finally to $CD8^+$ single-positive (SP) or $CD4^-CD8^-$ DN stages (Figure 1D). Importantly, the final cell product lacked $CD4^+$ helper or regulatory T cells, a population normally found in endogenous T cells (Figure 1D). Given that CD8 SP and DN T cells are generally associated with proinflammatory and cytotoxic properties, this phenotype is particularly favorable for cancer immunotherapy applications.

A key advantage of our HSPC-based approach is the generation of a clonally pure, mono-specific T cell product. By introducing the ESO-TCR during the early stages of hematopoietic development, the culture system leverages allelic exclusion and positive selection mechanisms to ensure that nearly all developing T cells express only the transgenic TCR (Figure 1E).[50] As

(B) Schematics design of Lenti/1G4 and Lenti/1G4-IL-15.
(C) Intracellular expression of ESO TCR (TCR Vβ13.1) in HSPCs at 72 h after lentivector transduction.
(D) FACS monitoring of $^{Allo/15}$ESO-T cell (TCR $V\beta13.1^+CD3^+$) generation over the 6-week culture.
(E) scTCR-seq analysis of $^{Allo/15}$ESO-T cell products. The relative abundance of each unique TCR sequence among the total unique sequences is represented by a pie slice.
(F) FACS analyses showing that the $^{Allo/15}$ESO-T generation is independent of matching MHC molecule.
(G–I) Antigen responses of $^{Allo/15}$ESO-T cells. (G) Cell growth curve ($n = 3$). (H) ELISA analyses of IFN-γ and TNF-α production on day 7 ($n = 3$). (I) ELISA analyses of IL-15 production on day 7 ($n = 3$).
(J) FACS detection of surface markers, cytokines, and cytotoxic molecules in $^{Allo/15}$ESO-T cells.
Representative of 1 (E) and more than 5 (A–D and F–J) independent biological replicates.
Data are presented as the mean ± SEM. ns, not significant; *, $p < 0.05$; **, $p < 0.01$; ****, $p < 0.0001$, by one-way ANOVA (H and I).

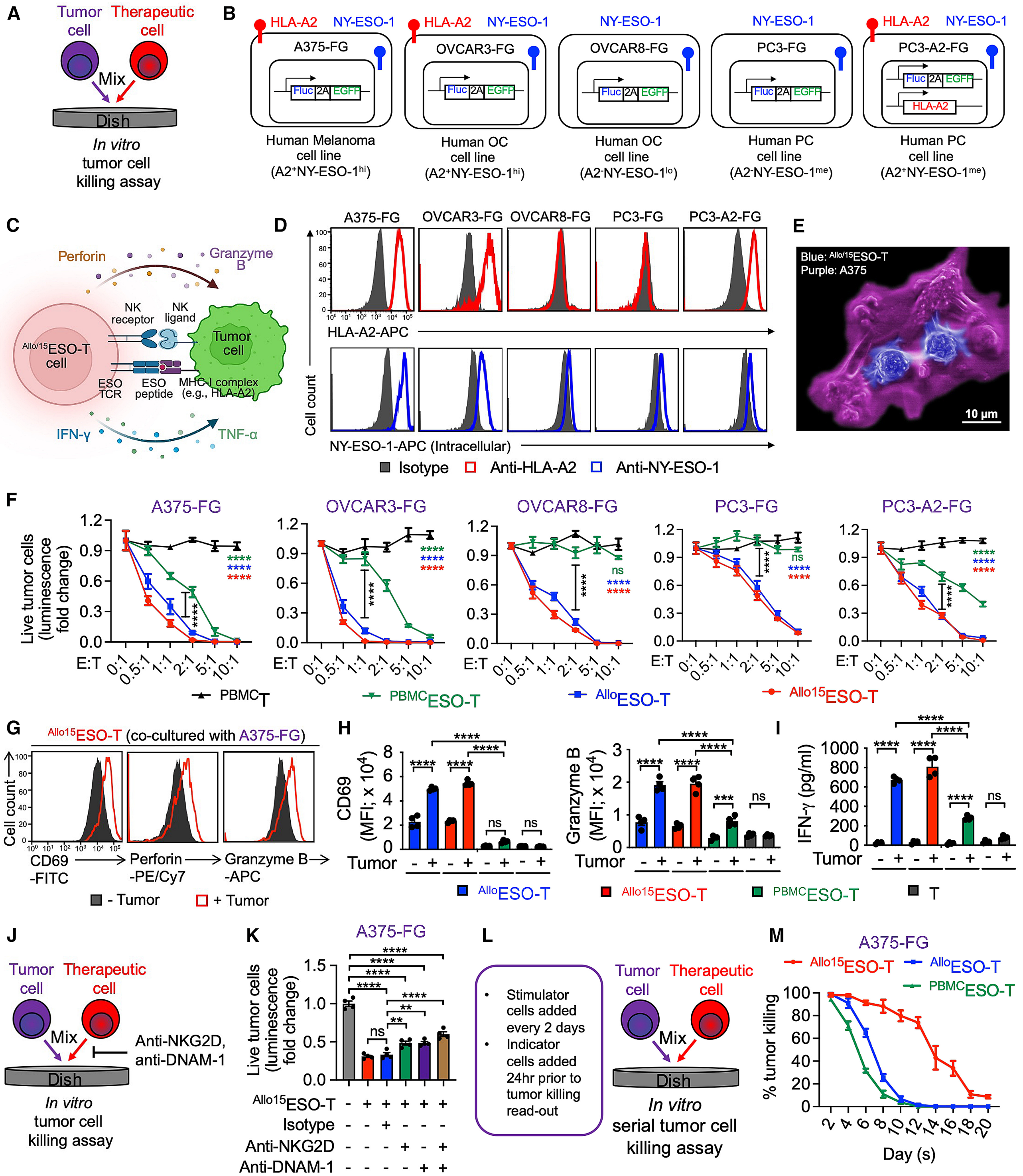

Figure 2. *In vitro* antitumor efficacy and MOA study of $^{Allo/15}$ESO-T cells

(A–F) Studying the *in vitro* antitumor efficacy of $^{Allo/15}$ESO-T cells against solid tumors. (A) Experimental design. (B) Schematics of human tumor cell lines. (C) Diagram showing the tumor-targeting mechanisms of $^{Allo/15}$ESO-T cells. (D) FACS detection of surface HLA-A2 and intracellular NY-ESO-1 expression on tumor cells. (E) Scanning electron microscope (SEM) image of an $^{Allo/15}$ESO-T cell (blue) attacking an A375 tumor cell (purple); scale bars, 10 μm. (F) Tumor cell killing data at 24 h (n = 4).

(G–I) Effector molecule expression in $^{Allo/15}$ESO-T cell cells co-cultured with A375-FG. (G) FACS detection of CD69 and intracellular perforin and granzyme B. (H) Quantification of G (n = 4). (I) ELISA of IFN-γ production (n = 4).

(legend continued on next page)

confirmed by single cell TCR sequencing (scTCR-seq), 100% of the resulting cells expressed the ESO TCR, with no detectable endogenous TCRs. This intrinsic mono-specificity eliminates the need for additional enrichment or purification steps and significantly reduces the risk of off-target immune responses or GvHD.[51] In contrast, PBMC-derived TCR-T (^{PBMC}ESO-T) cells retain endogenous TCR expression, creating risks of TCR mispairing,[33,52,53] HLA-dependent alloreactivity,[54–56] and increased manufacturing complexity associated with TCR knockout strategies (Figure 1E).[53,56] These limitations are inherently avoided in our HSPC-based system, which generated functional T cells irrespective of donor HLA type, enabling production from both HLA-A2-positive and HLA-A2-negative donors without compromising transgenic TCR function (Figure 1F). This HLA-independent developmental process supports universal donor flexibility and off-the-shelf manufacturing.

The platform was reproducible across eight independent cord blood donors (Figure S1A; Table S1). To further demonstrate its versatility, we generated NY-ESO-$1_{60–72}$/HLA-B7 complex-restricted ^{Allo}ESO-T (B7) cells, using an additional NY-ESO-1-specific TCR (clone 1E4), which exhibited comparable differentiation and phenotype to the HLA-A2 product while remaining independent of donor HLA type (Figures S1B–S1E).

Characterization of $^{Allo/15}$ESO-T cells

To evaluate the functionality of the transgenic ESO TCR, $^{Allo/15}$ESO-T cells were stimulated with the NY-ESO-$1_{157–165}$ peptide (Figures 1G–1I). The cells responded with robust proliferation (Figure 1G) and high levels of Th1-type cytokine secretion, including those of IFN-γ and TNF-α (Figure 1H), consistent with their CD8 SP and DN phenotype. Notably, ^{Allo15}ESO-T cells exhibited slightly greater proliferative activity than ^{Allo}ESO-T cells, which was associated with their robust endogenous IL-15 production (Figure 1I).

To further confirm the cytotoxic T cell identity of the $^{Allo/15}$ESO-T products, we performed comprehensive phenotypic characterization by flow cytometry. Compared with ^{PBMC}ESO-T cells, the $^{Allo/15}$ESO-T cells exhibited elevated expression of natural killer (NK) receptors (NKRs, e.g., NKG2D, DNAM-1, NKp30, and NKp44), activation markers such as CD69, and key effector molecules including IFN-γ, TNF-α, granzyme B, and perforin (Figure 1J). These features strongly support their cytotoxic potential and suitability for cancer immunotherapy. Importantly, HLA-B7-restricted ^{Allo}ESO-T (B7) cells displayed a comparable phenotype to their HLA-A2-restricted counterparts, further underscoring the consistency and adaptability of the platform (Figure S1F).

In vitro antitumor efficacy and MOA study of $^{Allo/15}$ESO-T cells

Given their cytotoxic and NK-like phenotype, we hypothesized that $^{Allo/15}$ESO-T cells recognize tumor cells through both TCR-dependent and NKR-mediated mechanisms. Specifically, $^{Allo/15}$ESO-T cells recognize NY-ESO-1 peptide presented by HLA-A2 while simultaneously engaging stress-induced ligands through activating NKRs, including NKG2D and DNAM-1 (Figure 2C).[57] This dual-targeting strategy may overcome tumor immune evasion that limits conventional TCR-mediated recognition.[58–61]

To evaluate this mechanism, we performed *in vitro* cytotoxicity assays, using five human solid tumor cell lines, including those of melanoma (A375-FG), ovarian cancer (OVCAR3-FG and OVCAR8-FG), and prostate cancer (PC3-FG- and HLA-A2-overexpressing PC3-A2-FG) (Figures 2A–2F). These cell lines were stably transduced to express a firefly luciferase and enhanced green fluorescent protein (EGFP), FG dual reporter, enabling precise quantification of tumor cell viability via bioluminescence and flow cytometry (Figure 2B). All cell lines expressed NY-ESO-1, while HLA-A2 expression varied among models (Figure 2D). PBMC-derived NY-ESO-1 tetramer$^+$ T cells were purified (>90% purity; Figure S2A) before co-culture to ensure comparable antigen-specific T cell frequencies. As expected, PBMC-derived ESO-T cells lysed only HLA-A2$^+$/NY-ESO-1$^+$ tumor cells, confirming strict MHC-restricted antigen recognition (Figure 2F).

In contrast, $^{Allo/15}$ESO-T cells efficiently lysed both HLA-A2$^+$ and HLA-A2$^-$ tumor cells (Figure 2F), indicating the presence of TCR-independent tumor recognition. Enhanced lysis of PC3-A2-FG (HLA-A2$^+$) compared to the parental PC3-FG (HLA-A2$^-$) cells further demonstrated the contribution of TCR signaling while supporting a dual-recognition mechanism. Scanning electron microscopy (SEM) imaging confirmed direct immune synapse formation between $^{Allo/15}$ESO-T cells and A375-FG tumor cells (Figure 2E).

Consistent with their superior cytotoxicity, $^{Allo/15}$ESO-T cells exhibited increased CD69 expression together with elevated production of perforin, granzyme B, and IFN-γ compared with ^{PBMC}ESO-T cells (Figures 2G–2I). To dissect the NKR-dependent component of tumor killing, we focused on NKG2D and DNAM-1, two well-characterized activating NKRs. Blocking these receptors significantly reduced killing of both HLA-A2$^+$ and HLA-A2$^-$ tumor cells (Figures 2J, 2K, and S2B–S2D), confirming their contribution to TCR-independent cytotoxicity.

We next evaluated the durability of antitumor activity. Serial tumor-killing assays demonstrated sustained cytotoxicity by ^{Allo15}ESO-T cells during repeated tumor challenge, with greater long-term activity than both ^{PBMC}ESO-T cells and ^{Allo}ESO-T cells lacking IL-15 (Figures 2L and 2M). These findings support a role for IL-15 in promoting the persistence and prolonged functional capacity of engineered T cells and led us to select ^{Allo15}ESO-T cells for subsequent *in vivo* studies.

To determine whether this dual-targeting mechanism extended across HLA contexts, we generated HLA-B7-restricted ^{Allo}ESO-T cells, using an alternative NY-ESO-1-specific TCR (clone 1E4). While ^{PBMC}ESO-T (B7) cells selectively

(J and K) Studying the tumor cell killing mechanisms of $^{Allo/15}$ESO-T cells mediated by NKG2D and DNAM-1. (J) Experimental design. (K) Tumor cell killing data at 24 h (E:T ratio = 0.5:1; $n = 4$).

(L and M) Studying the long-term antitumor efficacy of $^{Allo/15}$ESO-T cells. (L) Experimental design. (M) Tumor cell killing data measured over time ($n = 4$).

Representative of 3 independent biological replicates. Data are presented as the mean ± SEM. ns, not significant; **, $p < 0.01$; ***, $p < 0.001$; ****, $p < 0.0001$, by two-way ANOVA (F) or by one-way ANOVA (H, I, and K).

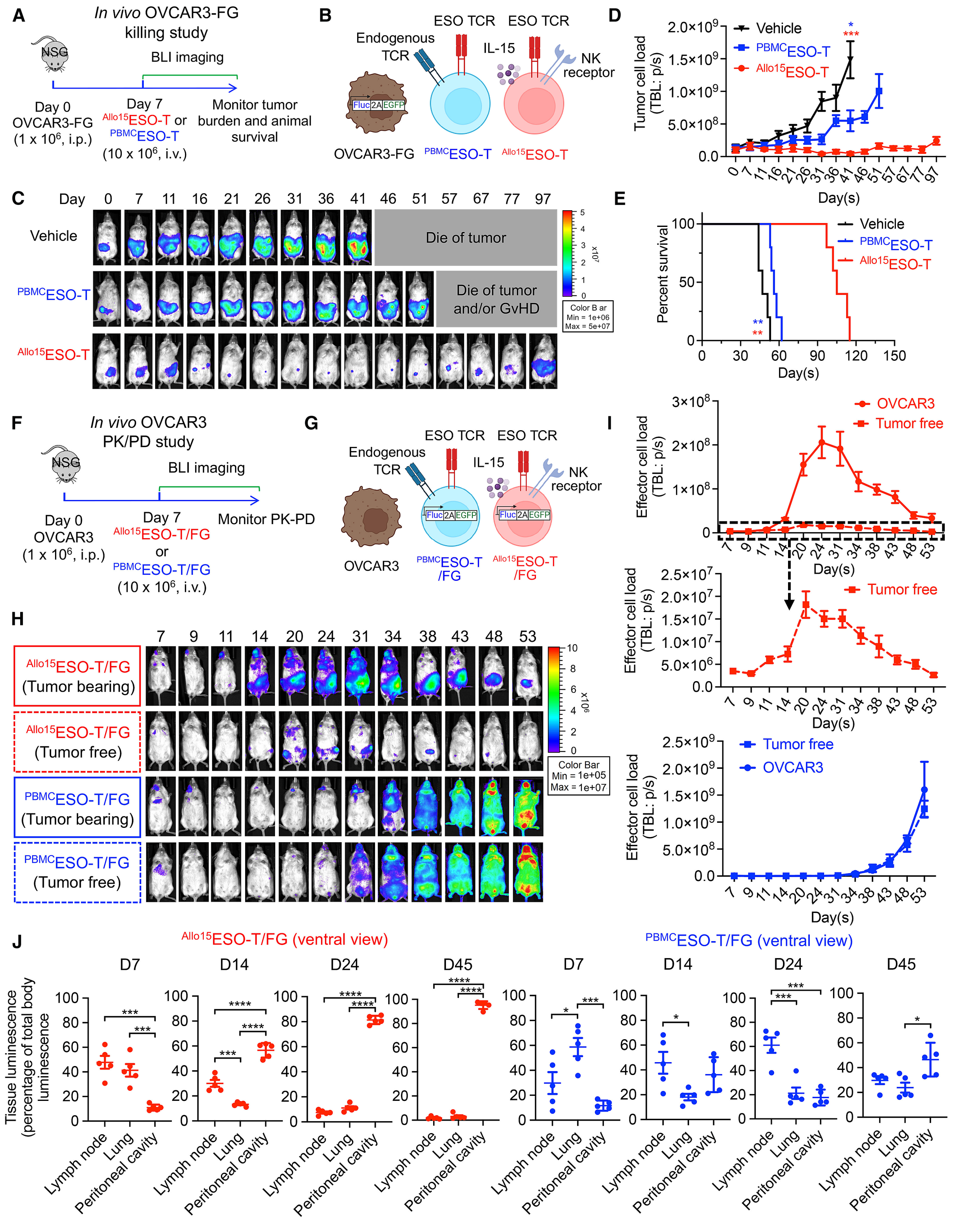

(legend on next page)

lysed HLA-B7$^+$/NY-ESO-1$^+$ targets, ^{Allo}ESO-T (B7) cells efficiently killed both HLA-B7$^+$ and HLA-B7$^-$ tumor cells, with enhanced activity against HLA-B7$^+$ targets (Figure S2E). These findings demonstrate that dual TCR- and NKR-mediated tumor recognition is maintained across distinct HLA-restricted TCRs.

In vivo antitumor efficacy and PK-PD study of ^{Allo15}ESO-T cells in an orthotopic OVCAR3 ovarian cancer xenograft mouse model

To investigate the *in vivo* antitumor efficacy and PK-PD characteristics of ^{Allo15}ESO-T cells, we conducted parallel mirror-image experiments, using a human orthotopic ovarian cancer xenograft model in NOD *scid* gamma (NSG) mice (Figure 3). Ovarian cancer was selected as the disease model due to the high prevalence of NY-ESO-1 expression, which is associated with poor prognosis and an aggressive clinical course.[20,62,63] Conventional ^{PBMC}ESO-T cells were included as a benchmark control.

In the efficacy study, HLA-A2$^+$ OVCAR3 tumor cells were engineered to express a dual firefly luciferase and EGFP reporter, FG reporter, enabling non-invasive tumor tracking (Figure 3B). In the PK-PD arm, ^{Allo15}ESO-T cells were similarly labeled with the FG reporter to visualize their *in vivo* expansion and biodistribution (Figure 3G). This design allowed for simultaneous monitoring of tumor burden and therapeutic cell dynamics throughout the treatment period.

Following intraperitoneal tumor implantation, a single dose of ^{Allo15}ESO-T cells resulted in stable tumor suppression and durable disease control, leading to long-term survival (>100 days) in all treated animals, though residual tumor signal was still detectable (Figures 3C–3E). In contrast, ^{PBMC}ESO-T cells achieved only partial tumor control and modest survival benefit, with all mice succumbing to disease progression and GvHD by approximately day 60 (Figures 3C–3E).

In the PK-PD study, ^{Allo15}ESO-T/FG cells underwent nearly 100-fold *in vivo* expansion, peaking between weeks 3 and 4 and followed by gradual contraction, with persistent presence detectable beyond 50 days (Figures 3H and 3I). Importantly, these cells exhibited strong and selective homing to the peritoneal cavity—the primary tumor site in this model—initiating migration by week 2 and continuing to expand and persist locally throughout the course of treatment (Figures 3H–3J). In tumor-free mice, ^{Allo15}ESO-T/FG cells exhibited a modest (~5-fold) expansion, also peaking around weeks 3–4, followed by a steady decline with prolonged persistence (Figures 3H and 3I). In this context, cells primarily homed to the bone marrow, a known site for HSPC-derived T cell residence.[38,64]

Conversely, ^{PBMC}ESO-T cells displayed continuous expansion in both tumor-bearing and non-tumor-bearing mice (Figures 3H and 3I), likely driven by either tumor-derived antigens or xenogeneic responses against murine tissues. This slow but aberrant expansion over time was associated with broad infiltration into major organs (Figure 3J), including the liver and lungs, and culminated in the onset of lethal GvHD. This phenotype reflects a progressive, systemic immune activation characterized by uncontrolled proliferation and widespread tissue damage.[65–67] Such long-term toxicity is a well-recognized limitation of PBMC-derived T cells, which retain diverse endogenous TCR repertoires capable of recognizing mouse antigens.[65–67] In contrast, ^{Allo15}ESO-T cells showed no evidence of GvHD or systemic infiltration into off-target tissues, even at peak expansion, supporting the safety of our mono-specific T cell products (Figures 3H–3J).

Notably, ^{Allo15}ESO-T cells demonstrated stable maintenance of the engineered TCR, with over 98% cells remaining TCR$^+$NY-ESO-1 tetramer$^+$ at day 63 post-infusion (Figure S4A). These findings indicate sustained antigen specificity, without evidence of endogenous TCR expression during prolonged *in vivo* persistence.

In vivo antitumor efficacy and PK-PD study of ^{Allo15}ESO-T cells in a metastatic A375 melanoma xenograft mouse model

To determine whether these findings generalized beyond localized disease, we evaluated ^{Allo15}ESO-T cells in a metastatic A375 melanoma xenograft model, using the same efficacy and PK-PD framework (Figure 4).

Following intravenous tumor injection, ^{Allo15}ESO-T cells administered as a single dose achieved durable tumor control and prolonged survival, with all treated animals surviving beyond 50 days (Figures 4C–4E). Although tumor relapse was eventually observed, the rate of progression was markedly delayed. In contrast, ^{PBMC}ESO-T cells conferred only transient control, with limited survival benefit; all animals in this group succumbed before day 40, primarily due to aggressive tumor progression and GvHD (Figures 4C–4E).

In the corresponding PK-PD study, ^{Allo15}ESO-T/FG cells demonstrated expansion kinetics comparable to those observed in the ovarian cancer model, with ~100-fold expansion and prolonged persistence (Figures 4H and 4I). Early in the response, therapeutic cells localized to the lungs—the initial tumor site (Figure 4C)—and effectively reduced tumor burden (Figure 4H). Additionally, ^{Allo15}ESO-T cells migrated to secondary metastatic sites of peritoneal cavity (Figure 4C), where they continued to expand and persist (Figures 4H–4J).

By contrast, ^{PBMC}ESO-T cells displayed dysregulated expansion in mice (Figures 4H and 4I). This was followed by extensive infiltration into major organs at day 25, culminating in rapid-onset GvHD (Figures 4H–4J). The accelerated kinetics of toxicity in this model—relative to the ovarian cancer—may be attributable to

Figure 3. *In vivo* antitumor efficacy and PK-PD study of ^{Allo15}ESO-T cells in an orthotopic ovarian cancer tumor model
(A–E) The *in vivo* antitumor efficacy of ^{Allo15}ESO-T cells in an OVCAR3-FG human ovarian cancer xenograft NSG mouse model. (A) Experimental design. (B) Schematics of tumor cells and therapeutic cells. (C) Bioluminescence imaging (BLI) for measuring tumor loads over time. (D) Quantification of data in (C); $n = 5$. (E) Kaplan-Meier survival curves ($n = 5$).
(F–J) Studying the *in vivo* PK-PD of ^{Allo15}ESO-T cells. (F) Experimental design. (G) Schematics of tumor cells and therapeutic cells. (H) BLI images showing the presence of therapeutic cells in experimental mice over time. (I) Quantification of data in (H); $n = 5$. (J) Quantification of therapeutic cell distribution over time ($n = 5$). Representative of 3 independent biological replicates. Data are presented as the mean ± SEM. *$p < 0.05$, **$p < 0.01$, ***$p < 0.001$, and ****$p < 0.0001$, by one-way ANOVA (D and J) or by log rank (Mantel-Cox) test adjusted for multiple comparisons (E).

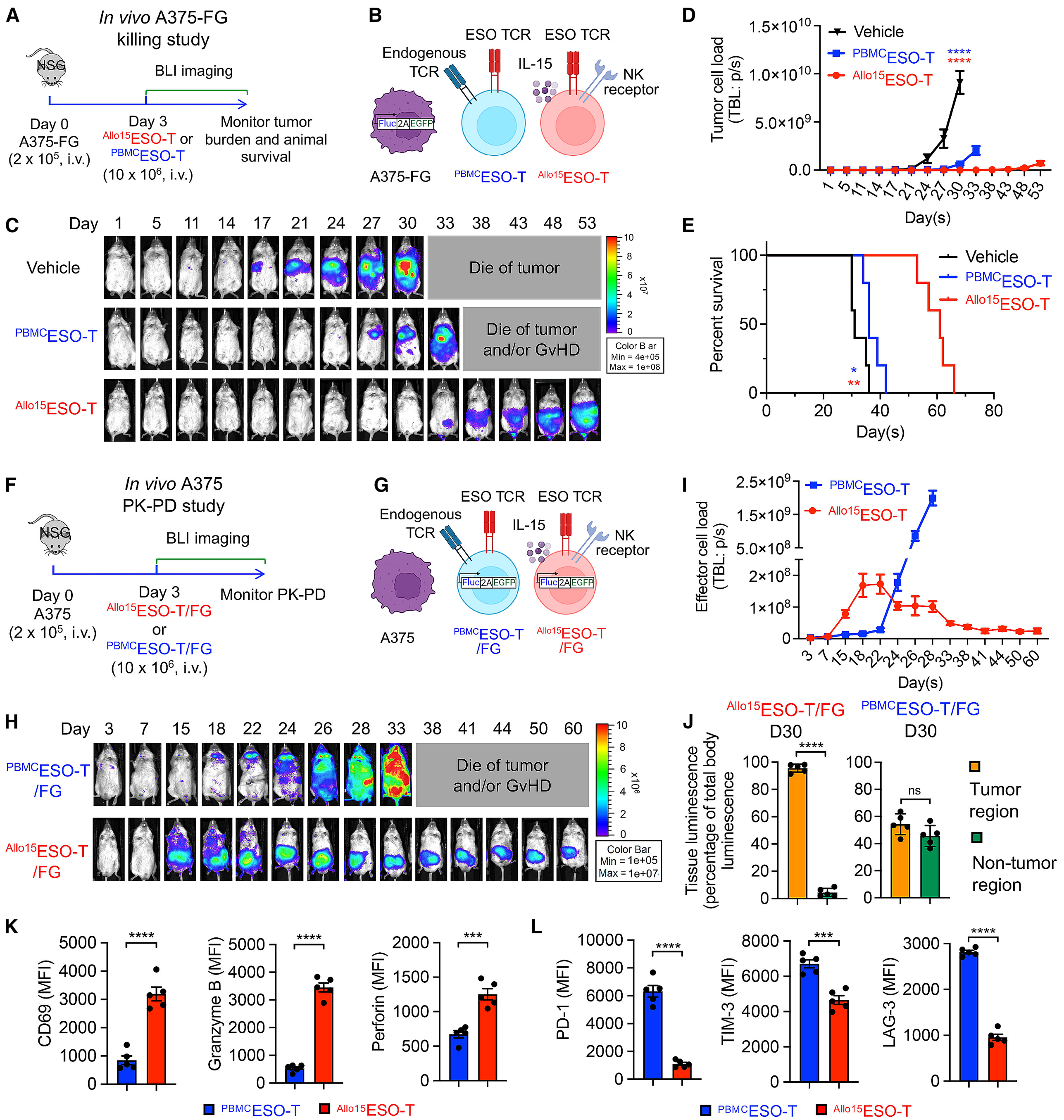

Figure 4. *In vivo* antitumor efficacy and PK-PD study of Allo15ESO-T cells in a metastatic melanoma tumor model

(A–E) Studying the *in vivo* antitumor efficacy of Allo15ESO-T cells in an A375-FG human melanoma xenograft NSG mouse model. (A) Experimental design. (B) Schematics of tumor cells and therapeutic cells. (C) BLI images measuring tumor load over time. (D) Quantification of data in (C); $n = 5$. (E) Kaplan-Meier survival curves ($n = 5$).

(F–J) Studying the *in vivo* PK-PD of Allo15ESO-T cells in an A375 human melanoma xenograft NSG mouse model. (F) Experimental design. (G) Schematics of tumor cells and therapeutic cells. (H) BLI images showing the presence of therapeutic cells over time. (I) Quantification of data in (H); $n = 5$. (J) FACS analyses of therapeutic cell distribution ($n = 5$).

(K and L) FACS analyses of (K) T cell activation marker and cytotoxic molecules ($n = 5$) and (L) immune checkpoint molecules ($n = 5$).

Representative of 3 independent biological replicates. Data are presented as the mean ± SEM. ns, not significant; *, $p < 0.05$; **, $p < 0.01$; ***, $p < 0.001$; ****, $p < 0.0001$, by Student's *t* test (J and K), one-way ANOVA (D), or log rank (Mantel-Cox) test adjusted for multiple comparisons (E).

CellPress
OPEN ACCESS

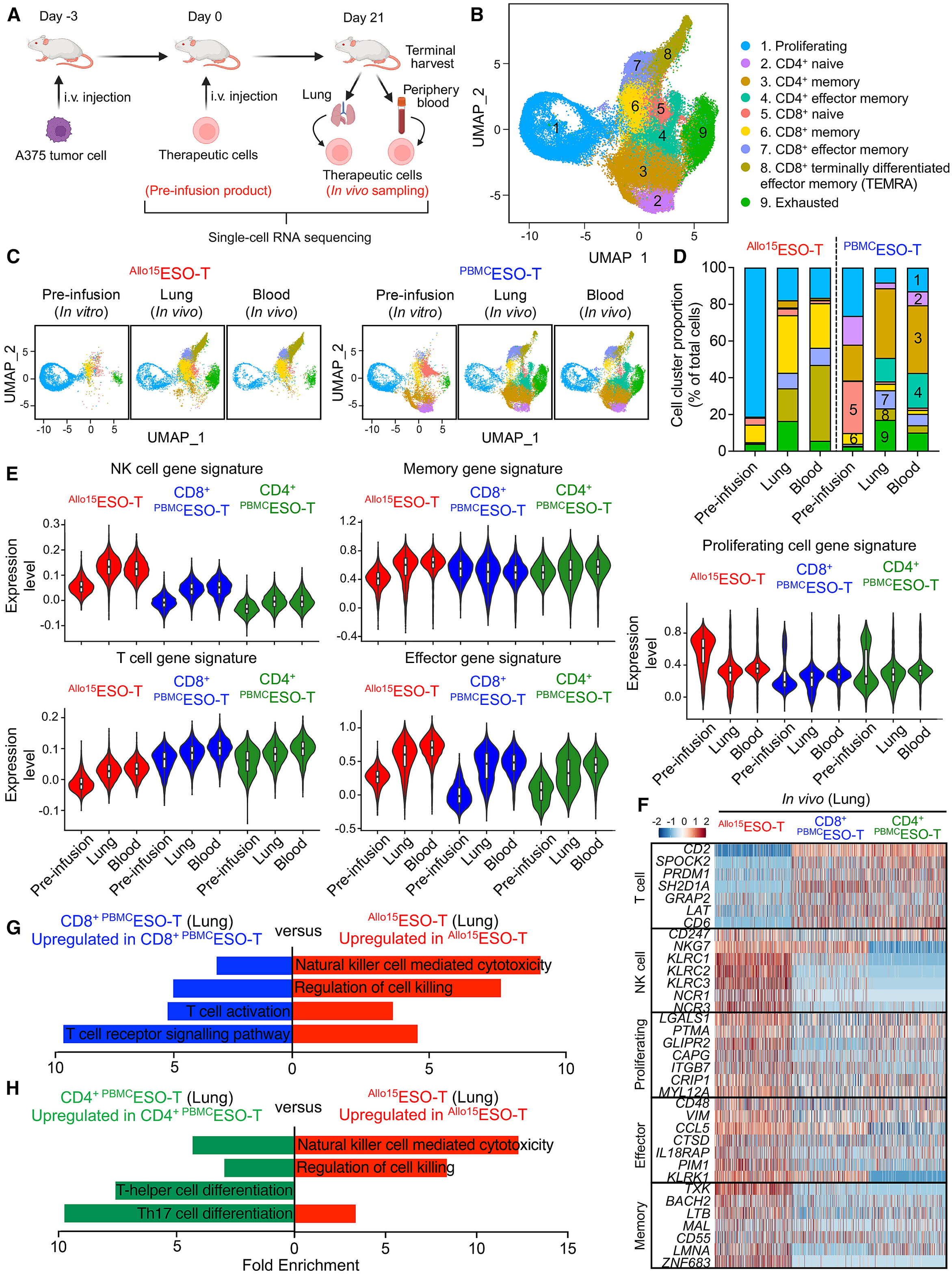

(legend on next page)

the lung-based tumor burden, which is the earliest site of ^{PBMC}ESO-T cell activation (Figures 4C and 4H–4J). ^{Allo15}ESO-T cells again showed no evidence of GvHD (Figures 4H–4J), consistent with their tumor-restricted localization and favorable safety profile.

To dissect the functional contributions of IL-15, we compared ^{Allo}ESO-T cells lacking IL-15 and ^{PBMC15}ESO-T cells expressing IL-15 (Figure S3A). ^{Allo}ESO-T cells exhibited limited *in vivo* persistence and failed to sustain tumor control (Figures S3B–S3D). In contrast, ^{PBMC15}ESO-T cells achieved robust and durable tumor suppression but developed severe GvHD, leading to mortality at approximately 1 month post-infusion (Figures S3B–S3D). These results highlight a critical role of IL-15 in promoting persistence and sustained antitumor efficacy, while also underscoring the importance of the allogeneic HSPC-derived platform in mitigating GvHD.

To characterize the functionality of therapeutic cells *in vivo*, we recovered ^{Allo15}ESO-T and ^{PBMC}ESO-T cells from treated animals around day 30 and analyzed their activation and exhaustion phenotypes (Figure 4K). ^{Allo15}ESO-T cells exhibited higher expression of the activation marker CD69 and elevated levels of cytotoxic effectors, including perforin and granzyme B. In contrast, ^{PBMC}ESO-T cells displayed increased expression of inhibitory immune checkpoint receptors PD-1, TIM-3, and LAG-3, suggesting early signs of exhaustion (Figure 4L).

Taken together, these findings demonstrate that the ^{Allo15}ESO-T cells maintain potent, tumor-specific effector function, a favorable activation profile, and minimal exhaustion across both orthotopic and metastatic models. Their ability to home dynamically to diverse tumor environments, expand in a controlled manner, and persist without systemic toxicity underscores their promise as a safe and effective off-the-shelf TCR-engineered cellular therapy.

In vivo gene profiling study of ^{Allo15}ESO-T cells

To elucidate the transcriptional programs underlying the *in vivo* antitumor function of ^{Allo15}ESO-T cells, we performed single-cell RNA sequencing (scRNA-seq) analysis using cells recovered from a human A375 melanoma xenograft mouse model in NSG mice (Figure 5A). ^{PBMC}ESO-T cells were included as a benchmark comparator. Therapeutic T cells—including ^{Allo15}ESO-T and ^{PBMC}ESO-T cells—were collected at day 0 (pre-infusion) and day 21 post-infusion from either the primary tumor site (lung) or peripheral blood (Figure 5A), representing baseline and *in vivo* tumor-responding states, respectively.

Uniform manifold approximation and projection (UMAP) of the combined dataset revealed nine major transcriptionally distinct clusters (Figure 5B). Based on canonical gene signatures, we identified cluster 1 cells as proliferating cells, cluster 2 cells as $CD4^+$ naive cells, cluster 3 cells as $CD4^+$ memory cells, cluster 4 cells as $CD4^+$ effector memory cells, cluster 5 cells as $CD8^+$ naive cells, cluster 6 cells as $CD8^+$ memory cells, cluster 7 cells as $CD8^+$ effector memory cells, cluster 8 cells as $CD8^+$ terminally differentiated effector memory (TEMRA) cells, and cluster 9 cells as exhausted cells (Figures 5B and S5A–S5C).[38,68–70]

Comparison of day 0 and day 21 samples revealed substantial transcriptional remodeling in both cell products post-infusion (Figures 5C and 5D), consistent with the notion that *in vivo* functionality cannot be fully predicted by the pre-infusion product.[71,72] Notably, day 21 corresponds to the contraction phase of ^{Allo15}ESO-T cell expansion and the early expansion phase of ^{PBMC}ESO-T cells, likely reflecting GvHD-related activation (Figures 4H and 4I).

Consistent with their engineered cytotoxic identity, ^{Allo15}ESO-T cells remained predominantly $CD8^+$ or $CD4^-CD8^-$ (DN), whereas ^{PBMC}ESO-T cells included both $CD4^+$ and $CD8^+$ subsets (Figures 5D and 1J). ^{PBMC}ESO-T cells also displayed greater heterogeneity across UMAP clusters, reflecting their polyclonal origin. At baseline, ^{Allo15}ESO-T cells were primarily composed of proliferating cells, while ^{PBMC}ESO-T cells contained a mix of naive and proliferative subsets (Figures 5C and 5D). By day 21, proliferating populations were diminished in both products, with ^{Allo15}ESO-T cells transitioning into dominant $CD8^+$ memory and $CD8^+$ TEMRA phenotypes across both lung and blood (Figures 5C and 5D). In contrast, ^{PBMC}ESO-T cells at day 21 exhibited a composition shift toward $CD4^+$ memory, $CD4^+$ effector memory, and $CD8^+$ effector memory states (Figures 5C and 5D). TEMRA cells, which were enriched in ^{Allo15}ESO-T populations, were described as a clonally expanded, tumor-reactive subset of $CD8^+$ tumor-infiltrating lymphocytes (TILs) that correlate with enhanced cytotoxicity in tumors and serve as predictive biomarkers of immunotherapy responsiveness.[68,73–76] The elevated representation of TEMRA cells in ^{Allo15}ESO-T populations is, thus, consistent with their observed functional potency *in vivo*.

Additionally, gene set enrichment analyses revealed fundamental differences between the two therapeutic platforms (Figures S5D–S5G). Compared with ^{PBMC}ESO-T cells, ^{Allo15}ESO-T cells showed upregulation of cytotoxicity-related pathways, including NK-mediated killing and cell death programs, in both lung and blood compartments (Figures S5D and S5E). In contrast, ^{PBMC}ESO-T cells were enriched for general T cell activation and differentiation pathways, reflecting broader immune engagement (Figures S5D and S5E). In the tumor site (lung), both therapeutic cells exhibited enrichment in metabolic

Figure 5. *In vivo* gene profiling study of ^{Allo15}ESO-T cells
(A) Schematic showing the experimental design to study the gene profiling of ^{Allo15}ESO-T cells using scRNA-seq.
(B) Combined UMAP plot showing the formation of nine major cell clusters. Total cells combined from all samples are included. Each dot represents a single cell and is colored according to its cell cluster assignment.
(C) Individual UMAP plots showing cell cluster composition of the indicated samples.
(D) Bar graphs showing the cell cluster proportions.
(E) Violin plots showing the expression distribution of NK, T, memory, effector, and proliferating gene signatures in the indicated groups.
(F) Heatmap showing the expression of representative signature genes. Each column indicates an individual cell. Each row indicates an individual gene.
(G and H) Pathway analyses of differentially expressed genes comparing lung-derived ^{Allo15}ESO-T cells with $CD8^+$ (G) and $CD4^+$ (H) ^{PBMC}ESO-T cells.
The experiment was performed once; cells isolated from five mice of each experimental group were combined for analyses.

CellPress
OPEN ACCESS

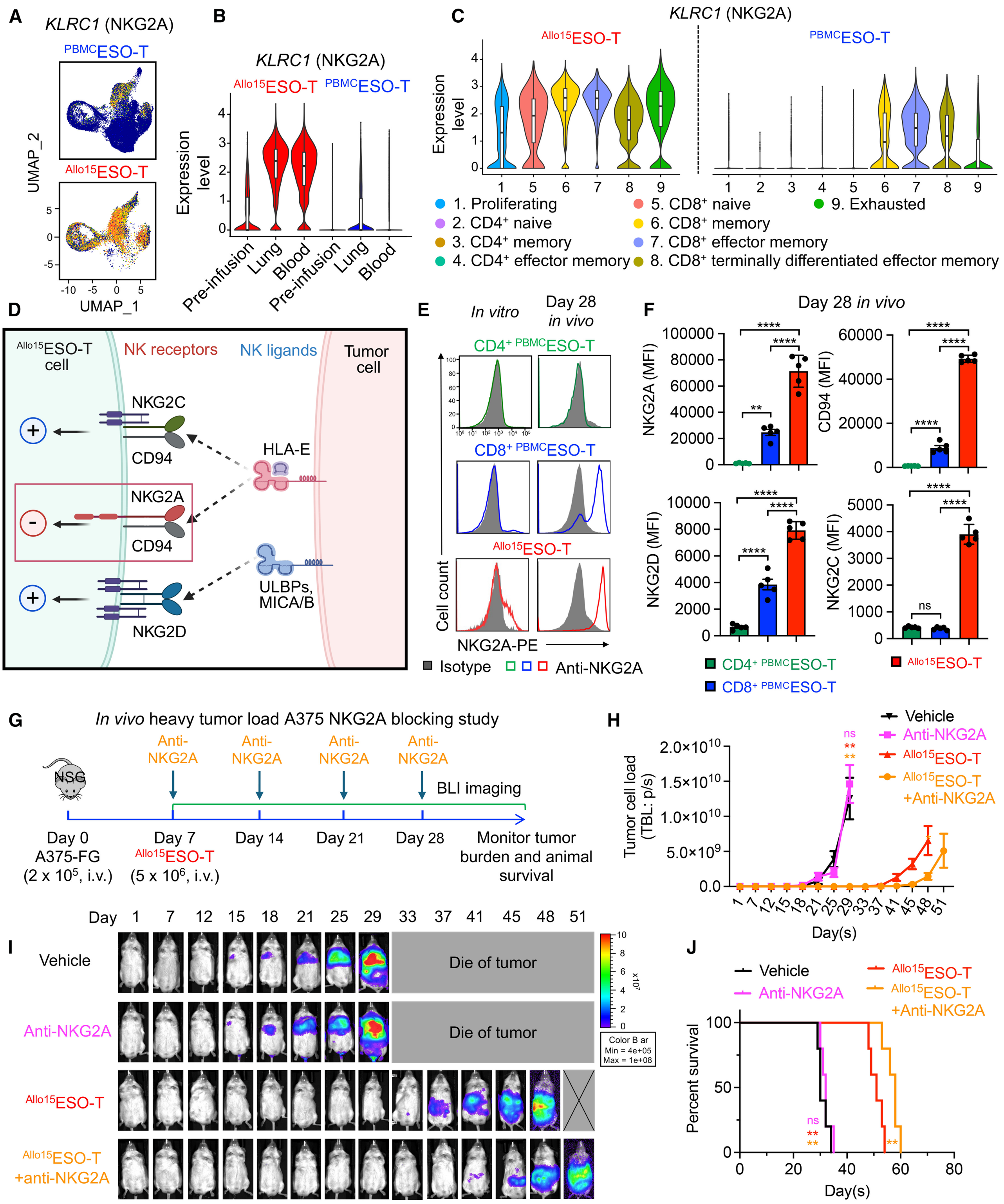

Figure 6. *In vivo* NKG2A immune checkpoint blockade study of Allo15ESO-T cells

(A–C) Studying the immune checkpoint molecule NKG2A (*KLRC1*) expression on Allo15ESO-T cells using scRNA-seq. (A) UMAP plots showing *KLRC1* expression in the indicated groups. (B) Violin plots showing *KLRC1* expression in the indicated samples. (C) Violin plots showing *KLRC1* expression in the indicated cell types. (D) Schematics showing the interplay between therapeutic cells and tumor cells mediated by the NKG2 immune regulatory network.

(legend continued on next page)

and biosynthesis processes (Figures S5F and S5G), suggesting heightened effector function and sustained activity.

To further dissect these differences, we stratified ^{PBMC}ESO-T cells into CD4^{+} and CD8^{+} SP populations and compared their transcriptomic profiles with those of ^{Allo15}ESO-T cells (Figure S5H). ^{Allo15}ESO-T cells exhibited stronger effector, memory, and proliferation-associated gene signatures than either CD4^{+} or CD8^{+} ^{PBMC}ESO-T subsets (Figure 5E), in line with their superior tumor control (Figures 4C–4E). Moreover, ^{Allo15}ESO-T cells expressed markedly fewer T cell markers and displayed elevated NK-like gene signatures—particularly at day 21 *in vivo*—consistent with their engineered cytotoxic profile (Figure 5E). Notably, CD8^{+} ^{PBMC}ESO-T cells exhibited more NK-like features than CD4^{+} ^{PBMC}ESO-T cells, though still lower than ^{Allo15}ESO-T cells (Figure 5E).

Given that these comparisons were performed at a single post-infusion time point, we next sought to determine whether the observed transcriptional differences could be influenced by differences in proliferation state or sampling timing. To address this, we performed additional phenotypic analyses across multiple stages, including pre-infusion, peak expansion, and contraction phases (Figures S4B–S4K). Consistent with the scRNA-seq findings, TEMRA-associated markers, including *PRF1*, *GZMB*, and *KLRD1*, were enriched in ^{Allo15}ESO-T cells at baseline and remained elevated following *in vivo* tumor challenge (Figures S4F–S4K). Notably, this enrichment was maintained and further increased at both peak expansion and contraction time points, indicating a sustained cytotoxic differentiation trajectory. In contrast, exhaustion marker expression (PD-1, TIM-3, and LAG-3) was comparable across cell products prior to infusion but diverged following *in vivo* activation (Figures S4B–S4E). ^{PBMC}ESO-cells exhibited increased exhaustion marker expression after tumor challenge, whereas ^{Allo15}ESO-T cells consistently maintained lower levels at both peak and contraction phases (Figures S4B–S4K).

Together, these results reveal that ^{Allo15}ESO-T cells adopt a transcriptionally distinct, functionally superior *in vivo* state marked by enhanced memory, effector, and NK-like profiles. Their monospecific nature contrasts with the heterogeneity of ^{PBMC}ESO-T cells, reinforcing the potential of ^{Allo15}ESO-T cells as a more effective allogeneic TCR-engineered cell therapy platform.

In vivo NKG2A immune checkpoint blockade study of ^{Allo15}ESO-T cells

Building upon our transcriptomic profiling of ^{Allo15}ESO-T cells, which revealed a strong NK-like cytotoxic program (Figure 5E), we next investigated the expression of immune checkpoint receptors that may limit their function within the TME (Figure 6).[77,78] Among canonical T cell and NK cell-associated inhibitory receptors, we identified NKG2A as a prominent checkpoint candidate expressed by ^{Allo15}ESO-T cells (Figure 6A). NKG2A (encoded by *KLRC1*) is an inhibitory receptor that forms a heterodimer with CD94 and transmits suppressive signals upon binding to HLA-E, antagonizing TCR and NKG2 receptor pathways.[79] Clinically, it has emerged as a promising immunotherapeutic target and is under investigation in combination with NK cell- and T cell-based adoptive therapies.[79–84]

Consistent with their NK-like transcriptional identity, ^{Allo15}ESO-T cells exhibited significantly higher *KLRC1* (NKG2A) mRNA expression than ^{PBMC}ESO-T cells, particularly in day 21 *in vivo* samples following tumor exposure, indicating activation-induced checkpoint upregulation (Figures 6A and 6B). Stratified cluster analysis revealed widespread *KLRC1* expression across ^{Allo15}ESO-T cells, while within ^{PBMC}ESO-T cells, *KLRC1* was primarily enriched in CD8^{+} subsets (Figure 6C).

To validate these findings at the protein level, we profiled surface expression of NKG2 family receptors (e.g., NKG2A, NKG2C, NKG2D, and CD94; Figures 6D–6F). In line with the transcriptomic data, NKG2A levels were low at baseline in both ^{Allo15}ESO-T and CD8^{+} ^{PBMC}ESO-T cells but increased substantially following *in vivo* activation (Figures 6D and 6E). Importantly, ^{Allo15}ESO-T cells also expressed high levels of CD94 and the activating receptors NKG2D and NKG2C (Figure 6F). Although NKG2A and NKG2C share HLA-E as a common ligand, they transmit opposing functional signals: NKG2A delivers inhibitory signals, whereas NKG2C promotes cytotoxic activity.[79,80]

The co-expression of inhibitory and activating NKG2 receptors suggests a regulatory balance in ^{Allo15}ESO-T cells, which can be therapeutically shifted by targeting NKG2A to relieve inhibitory signaling and enhance antitumor activity. To test this, we used the A375-FG human melanoma xenograft model with increased tumor burden to mimic a more immunosuppressive, exhaustion-prone microenvironment (Figure 6G). Mice received ^{Allo15}ESO-T cells with or without NKG2A blockade via administration of monalizumab, a clinically tested anti-NKG2A monoclonal antibody.[85] Notably, NKG2A blockade enhanced the antitumor activity of ^{Allo15}ESO-T cells, as evidenced by reduced tumor burden and prolonged survival (Figures 6H–6J). These findings highlight the potential of targeting NK-associated immune checkpoints, particularly NKG2A, to further enhance the efficacy of our TCR-engineered cytotoxic T cells.

Safety and immunogenicity study of ^{Allo15}ESO-T cells

A key challenge in allogeneic cell therapy is balancing efficacy with safety, particularly with respect to GvHD, host-mediated allorejection, and cytokine release syndrome (CRS). GvHD arises when infused allogeneic T cells recognize and attack host tissues, leading to systemic inflammation and organ damage.[86–89] Conversely, allorejection—mediated by host CD4^{+}, CD8^{+}, and NK cells—can compromise therapeutic persistence and efficacy.[90,91] CRS, a severe inflammatory response commonly associated with T cell therapy in solid tumors, also remains a significant safety concern.[92,93]

(E and F) FACS detection of NKG2A (E) and NKG2 (F) family proteins expressed in ^{Allo15}ESO-T cells ($n = 5$).
(G–J) Studying the impact of NKG2A blockade on antitumor efficacy of ^{Allo15}ESO-T cells in an A375-FG xenograft mouse model. (G) Experimental design. (H) BLI images measuring tumor load over time. (I) Quantification of data in (H); $n = 5$. (J) Kaplan-Meier survival curves.
Representative of 1 (A–C) or 3 (E–J) independent biological replicates. Data are presented as the mean ± SEM. ns, not significant; **, $p < 0.01$; and ****, $p < 0.0001$, by one-way ANOVA (F and H) or by log rank (Mantel-Cox) test adjusted for multiple comparisons (J).

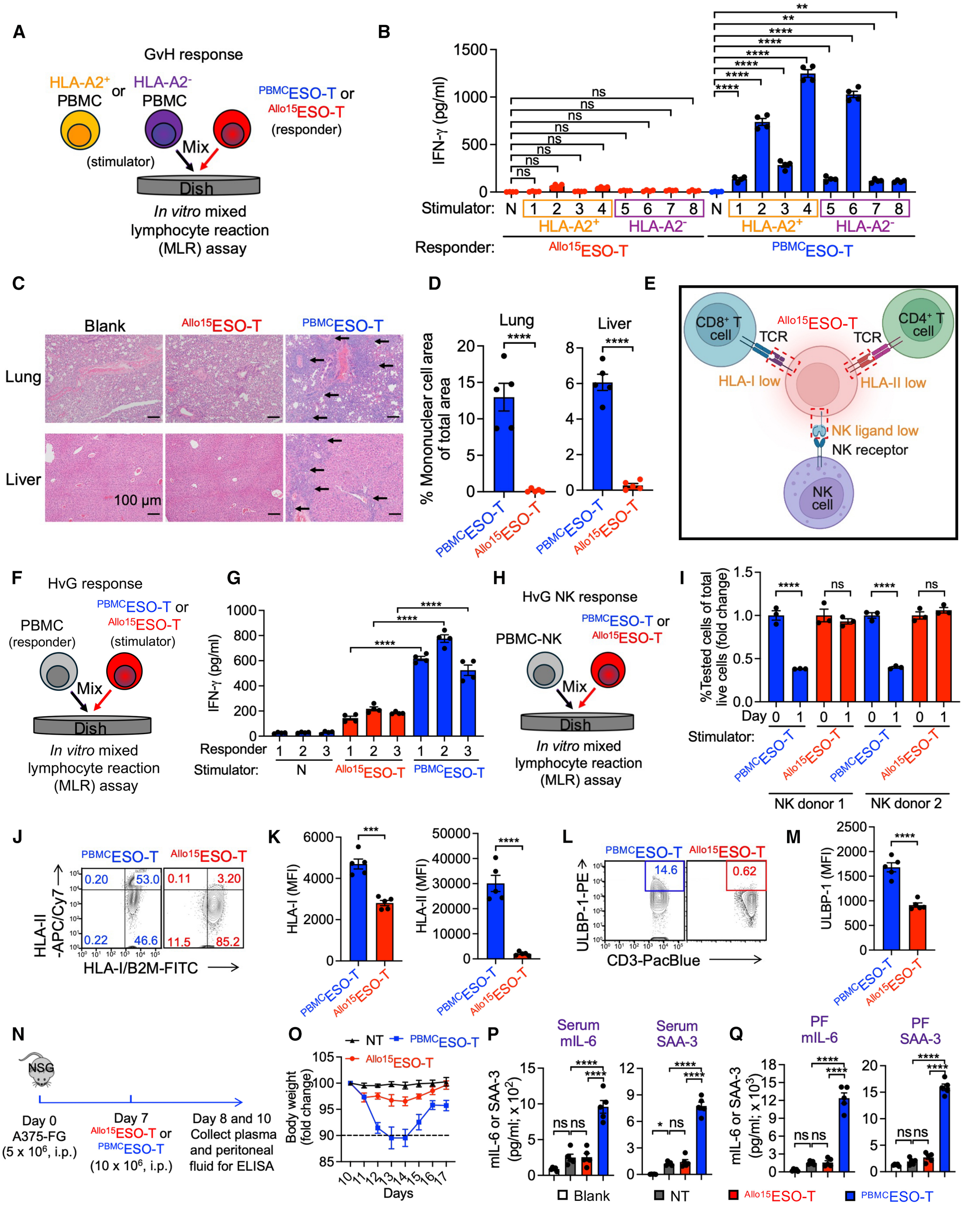

Figure 7. Safety and immunogenicity study of ^{Allo15}ESO-T cells

(A and B) Studying the GvH response of ^{Allo15}ESO-T cells using an *in vitro* MLR assay. Stimulator PBMCs from both HLA-A2^{+} and HLA-A2^{-} donors were analyzed. (A) Experimental design. (B) ELISA of IFN-γ production on day 4. N, no addition of stimulator PBMCs (n = 4).

(legend continued on next page)

We first evaluated the GvHD risk of ^{Allo15}ESO-T cells relative to ^{PBMC}ESO-T cells (Figures 7A–7D). ^{Allo15}ESO-T cells are engineered to express a mono-specific TCR targeting NY-ESO-1 presented by HLA-A2 and, thus, are not expected to elicit GvHD, as NY-ESO-1 is not expressed on healthy PBMCs. This hypothesis was tested using both an *in vitro* mixed lymphocyte reaction (MLR) and an *in vivo* xenograft model (Figures 7A–7D). In the MLR assay, ^{Allo15}ESO-T cells were co-cultured with irradiated PBMCs from 8 unmatched donors (4 HLA-A2$^+$ and 4 HLA-A2$^-$) (Figure 7A). Minimal IFN-γ production was observed in all cases, in stark contrast to the robust IFN-γ secretion observed from ^{PBMC}ESO-T cells, which likely reflects activation via their diverse endogenous TCRs (Figure 7B).

Infusion of ^{PBMC}ESO-T cells into tumor-bearing NSG mice resulted in severe GvHD, with associated mortality and extensive immune infiltration into vital organs such as the liver, lung, kidney, spleen, and heart (Figures 7C, 7D, S6C, and S6D). In contrast, ^{Allo15}ESO-T-treated mice showed no signs of GvHD, with long-term survival and absence of immune infiltration (Figures 7C and 7D). To further assess tissue distribution, we performed immunohistochemical analyses of major organs in both tumor-bearing and non-tumor-bearing mice following ^{Allo15}ESO-T infusion. Minimal infiltration of ^{Allo15}ESO-T cells was observed across these organs at late time points (Figure S6B). Additionally, tissues collected after ^{Allo15}ESO-T infusion were assessed by blinded pathological scoring across multiple organs. No appreciable abnormalities were observed, supporting the absence of delayed tissue toxicity.

We next evaluated the susceptibility of ^{Allo15}ESO-T cells to host allorejection, mediated by T cell recognition of mismatched HLA molecules[94] and NK cell responses triggered by "missing-self" and stress-induced ligands[95–97] (Figures 7E–7M). To assess rejection driven by host T cells, we performed an MLR in which irradiated ^{Allo15}ESO-T or ^{PBMC}ESO-T cells were co-cultured with allogeneic PBMCs from three HLA-mismatched donors (Figure 7F). Compared with ^{PBMC}ESO-T cells, ^{Allo15}ESO-T cells elicited markedly reduced IFN-γ secretion, indicative of diminished allo-stimulatory potential (Figure 7G). To evaluate NK cell-mediated rejection, we co-cultured ^{Allo15}ESO-T and ^{PBMC}ESO-T cells with PBMC-derived NK cells from two unmatched donors (Figure 7H). ^{Allo15}ESO-T cells demonstrated significantly enhanced survival relative to ^{PBMC}ESO-T cells (Figure 7I), suggesting reduced sensitivity to innate immune rejection.

To elucidate the molecular basis of this hypoimmunogenic phenotype, we profiled cell surface molecules associated with immune recognition (Figures 7J–7M). ^{Allo15}ESO-T cells expressed markedly lower levels of HLA class I, HLA class II, and NK ligand ULBP-1 than ^{PBMC}ESO-T cells (Figures 7J–7M and S6A), and this expression profile likely contributes to the evasion of ^{Allo15}ESO-T cells from host T and NK cell-mediated allorejection.[98] We further evaluated CRS risk by using a previously established preclinical model.[93] ^{Allo15}ESO-T cell-treated mice exhibited less weight loss and significantly lower levels of CRS-associated inflammatory biomarkers, including IL-6 and SAA-3, in both serum and peritoneal fluid (Figures S7N–S7Q), relative to ^{PBMC}ESO-T cells.

Together, these findings demonstrate that our HSPC-engineered ^{Allo15}ESO-T cells exhibit a hypoimmunogenic and non-alloreactive phenotype, characterized by low GvHD risk, reduced susceptibility to host immune rejection, and attenuated CRS toxicity. This safety profile appears intrinsic to their mono-specific TCR identity and reduced expression of alloreactivity-associated molecules, supporting their potential as a next-generation allogeneic T cell product.

Development of HLA-ablated NY-ESO-1 specific universal T cells

To further advance ^{Allo15}ESO-T cells as a clinically viable allogeneic cytotoxic T cell platform for solid tumors, we sought to enhance both their safety and persistence. Although ^{Allo15}ESO-T cells exhibit an HLA-I/II^{low} phenotype (Figures 7J and 7K), residual HLA expression may still render them susceptible to host T cell-mediated allorejection. To address this, we developed a "universal" cytotoxic product (^{U}ESO-T) by genetically ablating surface expression of both HLA-I and HLA-II molecules (Figure S7).

Complete ablation of HLA surface expression can be achieved by targeting two key regulators: B2M, encoding β2-microglobulin essential for HLA class I assembly and surface expression, and CIITA, the master transcriptional activator of HLA class II genes.[67,99] Using a CRISPR-Cas9 system with guide RNAs against *B2M* and *CIITA*, we performed gene editing in CD34$^+$ HSPCs, in parallel with lentiviral transduction of a 1G4 ESO-TCR. This vector also co-expressed HLA-E, an NK cell-inhibiting ligand, and a suicide gene (*sr39TK*), providing both immunoevasion and a safety switch for clinical translation (Figure S7A).[99,100]

The resulting ^{U}ESO-T cells demonstrated high efficiency of gene engineering and robust HLA-E expression (Figure S7B).

(C and D) Studying the GvHD risk of ^{Allo15}ESO-T cells using a human A375 xenograft mouse model. (C) Hematoxylin and eosin-stained tissue sections. Tissues were collected on day 35. Scale bars, 100 μm.
(D) Quantification of data in (C); $n = 5$.
(E) Working model illustration of ^{Allo15}ESO-T cell hypoimmunogenecity.
(F and G) Studying the T cell-mediated allorejection of ^{Allo15}ESO-T cells. (F) Experimental design. (G) ELISA of IFN-γ production on day 4 ($n = 4$).
(H and I) Studying the NK cell-mediated allorejection of ^{Allo15}ESO-T cells. (H) Experimental design. (I) FACS analyses of the indicated live cells ($n = 3$).
(J and K) Studying HLA-I/II expression in ^{Allo15}ESO-T cells. (J) FACS detection of HLA-I and HLA-II expressions. (K) Quantification of data in (J); $n = 5$.
(L and M) Studying NK ligand expression in ^{Allo15}ESO-T cells. (L) FACS detection of ULBP-1 expression. (M) Quantification of data in (L); $n = 5$.
(N–Q) Studying CRS induced by ^{Allo15}ESO-T cells using an *in vivo* human A375 xenograft mouse model. (N) Experimental design. (O) Body weight measured over time; $n = 4$. (P and Q) ELISA analyses of mouse IL-6 and SAA-3 in serum (P) or peritoneal fluid (Q); $n = 4$. SAA-3, serum amyloid A-3; NT, samples collected from tumor-bearing mice receiving no therapeutic treatment.
Representative of 3 independent biological replicates. Data are presented as the mean ± SEM. ns, not significant; *, $p < 0.05$; **, $p < 0.01$; ***, $p < 0.001$; and ****, $p < 0.0001$, by Student's *t* test (D, K, and M) or by one-way ANOVA (B, G, L, P, and Q).

CellPress
OPEN ACCESS

These cells developed along trajectories comparable to ^{Allo}ESO-T cells and exhibited no defects in HSPC differentiation or lineage specification (Figures 1D and S7D). CRISPR-Cas9 editing resulted in a final ^{U}ESO-T cell product comprising approximately 50% HLA class I/II double-knockout cells (Figure S7E); if necessary, this population can be further enriched by magnetic-activated cell sorting using B2M and HLA-II antibodies.

Despite complete HLA ablation, ^{U}ESO-T cells displayed a typical cytotoxic T cell phenotypes and exhibited potent antitumor activity comparable to parental ^{Allo}ESO-T cells (Figures S7F and S7G). In immunogenicity assays, ^{U}ESO-T cells elicited minimal alloreactive responses from allogeneic PBMCs (Figures S7H and S7I) and remained resistant to NK cell-mediated rejection (Figures S7J and S7K). Furthermore, the incorporated *sr39TK* gene conferred robust sensitivity to ganciclovir, enabling conditional elimination of ^{U}ESO-T cells *in vitro* (Figures S7L and S7M).

Collectively, these findings highlight the versatility of our scalable HSPC-engineered mono-specific cytotoxic T cell platform, demonstrating its capacity to accommodate further gene modifications—such as HLA ablation and NK evasion—for enhanced safety and persistence in allogeneic solid tumor immunotherapy.

DISCUSSION

In this study, we report the development and characterization of an allogeneic platform for generating ^{Allo}ESO-T cells from HSPCs. Using an *ex vivo*, feeder-free T cell differentiation system, we achieved efficient production of mono-specific cytotoxic T cells with high yield, purity, and antitumor activity. These engineered cells demonstrated dual-targeting capacity, reduced GvH and host-versus-graft (HvG) responses, and compatibility with downstream genetic modifications such as CRISPR-Cas9-mediated gene editing. Together, these results establish the feasibility of using HSPCs to generate allogeneic, antigen-specific T cells as a scalable and modular platform for cancer immunotherapy.

The field of ACT is rapidly moving toward allogeneic "off-the-shelf" products. Current approaches primarily rely on PBMC-derived αβ T cells, NK cells,[90,94,101] or iPSC-based systems.[40,102,103] PBMC-derived αβ T cells require extensive gene editing to eliminate endogenous TCR expression and prevent GvHD,[104–107] whereas NK cells generally exhibit limited expansion and persistence.[101] iPSC-based platforms enable standardized, renewable manufacturing[108–112] but typically involve multi-step differentiation, additional engineering, and prolonged production timelines.[39,40,103,113] These limitations highlight the need for simpler, scalable allogeneic T cell manufacturing strategies.

Our feeder-free HSPC differentiation system addresses several of these limitations by facilitating the generation of antigen-specific T cells without endogenous TCR rearrangement (Figure 1E), and it achieves ∼10^6-fold expansion within 5 weeks and consistently produces a uniform mono-specific product across multiple cord blood donors despite donor-to-donor variability (Figures 1A and S1A). Unlike conventional PBMC-derived approaches, this platform eliminates the need for endogenous TCR knockout while remaining readily adaptable to alternative tumor-specific TCRs. From a single cord blood donor (∼5 × 10^6 HSPCs), our system is projected to generate ∼5 × 10^{12} ^{Allo}ESO-T cells, sufficient for 1,000–10,000 therapeutic doses (Figure S1). This scalability is enabled by TCR-guided differentiation through allelic exclusion, which produces highly pure T cell products with minimal conventional αβ T cell contamination.[50,114,115] Moreover, the system can generate T cells expressing diverse MHC-restricted TCRs independent of donor HLA type (Figures 1D and S1C). This flexibility provides a practical framework for developing libraries of off-the-shelf TCR-T products targeting shared tumor antigens across multiple HLA restrictions.[40,103]

^{Allo}ESO-T cells demonstrated potent antitumor efficacy *in vitro* and *in vivo*, outperforming PBMC-derived TCR-T cells. These cells displayed a cytotoxic phenotype, with elevated expression of proinflammatory cytokines, effector molecules, and NKR (e.g., NKG2D and DNAM-1), supporting a dual tumor-targeting mechanism through both TCR- and NKR-mediated cytotoxicity (Figures 1J and 2K). In ovarian cancer and melanoma models, ^{Allo}ESO-T cells exhibited superior tumor control, together with robust tumor-specific expansion (Figures 3 and 4). Notably, ^{Allo}ESO-T cells preferentially homed to tumor sites rather than peripheral organs. scRNA-seq analysis further revealed enrichment of TEMRA cells and upregulation of effector/memory gene signatures, corroborating their cytotoxic phenotype and enhanced functionality (Figure 5).

In addition to their potent antitumor activity, ^{Allo}ESO-T cells exhibited a favorable safety profile. The absence of endogenous TCRs eliminated detectable GvH reactivity *in vitro* and *in vivo* without requiring endogenous TCR knockout, providing a key safety advantage over conventional allogeneic T cell products (Figures 7A and 7B). Furthermore, ^{Allo}ESO-T cells exhibited reduced CRS-associated toxicity (Figures 7N–7Q) and resisted host immune rejection mediated by both T cells and NK cells (Figures 7F–7I), likely through reduced expression of HLA class I/II molecules and NK-activating ligands (Figures 7L and 7M). These features collectively promote prolonged persistence of allogeneic T cells in the host without the need for extensive lymphodepleting preconditioning.

Finally, our platform demonstrated strong compatibility with additional engineering strategies, including CRISPR-Cas9-mediated gene editing and safety switches (Figure S7). To further enhance safety, we incorporated CRISPR-Cas9-mediated knockout of HLA class I and II molecules (Figure S7E), together with a suicide switch (sr39TK/GCV), enabling efficient pharmacologic elimination of engineered cells (Figures S7L–S7M). Importantly, these modifications were introduced at the HSPC stage without impairing T cell differentiation or antitumor function, highlighting the flexibility of the platform for engineering. These strategies may further reduce HvG rejection and lessen the need for lymphodepleting preconditioning.[104,105] Beyond improving safety, we also explored immune checkpoint modulation to further enhance therapeutic efficacy. Transcriptomic and functional analyses identified NKG2A as a key inhibitory receptor limiting ^{Allo}ESO-T cell cytotoxicity (Figures 6A–6F). Consistent with this finding, NKG2A blockade significantly enhanced antitumor responses *in vivo* (Figures 6G–6J), identifying NKG2A as a promising target for combination therapy with allogeneic TCR-T cells.

Collectively, these findings highlight the modularity of our platform, which readily accommodates additional engineering strategies—including alternative targeting receptors, cytokine support, and checkpoint modulation—to further enhance persistence, overcome tumor immunosuppression, and optimize next-generation off-the-shelf TCR-T therapies for solid tumors.

Limitations of the study

Despite its promise, the off-the-shelf ^{Allo}ESO-T platform has several limitations that warrant refinement. First, reliance on NY-ESO-1 targeting constrains patient coverage and risks antigen escape; evaluating additional clinically validated TCRs (e.g., MAGE-A4 and PRAME) within the same feeder-free HSPC differentiation workflow should broaden applicability without introducing major manufacturing changes. Second, while IL-15 supports persistence, alternative or combinatorial cytokine modules (e.g., IL-12, IL-18, or IL-21) merit systematic testing to optimize fitness in immunosuppressive TMEs.[116] Third, the observed benefit of NKG2A blockade was modest; unbiased analysis of other inhibitory nodes (e.g., PD-1/PD-L1, TIGIT, TIM-3, LAG-3, and CTLA-4) and rational multiplex editing may yield larger gains.[117] Finally, safety, comparability, and CMC robustness—including on-/off-target risks, cytokine-related toxicities such as CRS, release criteria, and dose scaling—require prospective validation in clinical settings. Although a low CRS profile was observed in the NSG mouse model, these systems lack key human immune components and may underestimate cytokine-mediated toxicities. Therefore, further evaluation in more clinically relevant models and early phase trials will be necessary to fully assess CRS risk and establish a safe therapeutic window.

RESOURCE AVAILABILITY

Lead contact

Requests for further information and new reagents generated in this study may be directed to and will be fulfilled by the lead contact, Lili Yang (liliyang@ucla.edu).

Materials availability

This study did not generate new unique materials.

Data and code availability

- Data: The scRNA-seq data generated in this study have been deposited in the public repository Gene Expression Omnibus (GEO) database (RRID: SCR_005012) and are accessible at GEO: GSE301248.
- Code: This paper does not report original code.
- Any additional information required to reanalyze the data reported in this paper is available from the lead contact upon request.

ACKNOWLEDGMENTS

We thank the UCLA animal facility for providing animal support; the UCLA Translational Pathology Core Laboratory for providing histology support; the UCLA Technology Centre for Genomics & Bioinformatics facility for providing RNA-seq services; the UCLA CFAR Virology Core for providing human cells; and the NIH tetramer core facility for provision of MHC-peptide complexes. This work was supported by Partnering Opportunity for Discovery Stage Research Projects Awards and Translational Research Projects Awards from the California Institute for Regenerative Medicine (DISC2-11157, DISC2-13015, TRAN1-12250, and TRAN1-16050 to L.Y.). Y. Zhu is a predoctoral fellow supported by a Whitcome predoctoral fellowship in molecular biology. Y.-R.L. is a postdoctoral fellow supported by a UCLA Chancellor's Award for Postdoctoral Research and a Goodman-Luskin Microbiome Center Collaborative Research Fellowship award.

AUTHOR CONTRIBUTIONS

Y. Zhu, J.Y., Y-R.L., and L.Y. designed the experiments, analyzed the data, and wrote the manuscript; L.Y. and Y-R.L. conceived and oversaw the study, with assistance from Y. Zhu and J.Y.; Y. Zhang and J.Y. performed all experiments, with assistance from Y.J.K, Y.T., Z. Li, Y.C., Z. Lyu, A.S.Z., N.M., C.Z., A.K., M.W, R.H., Y-C.W., X.S., Z.H., and Y. Zhang; E.Z. helped with scanning electron microscope imaging; S.L. and A.W. helped with analysis of RNA-seq data.

DECLARATION OF INTERESTS

Y.-R.L., J.Y., Y.J.K., Z. Li, and L.Y. are inventors on patents relating to this study filed by UCLA; J.Y. is currently an employee of Legend Biotech. Y.J.K. is currently an employee of Nkarta; Z. Li is currently an employee of Allogene; L.Y. is a scientific advisor to AlzChem and Amberstone Biosciences. None of the declared companies contributed to or directed any of the research reported in this article.

STAR★METHODS

Detailed methods are provided in the online version of this paper and include the following:

- KEY RESOURCES TABLE
- EXPERIMENTAL MODEL AND STUDY PARTICIPANT DETAILS
  - Mice
  - Cell lines
  - Human CD34^{+} HSPCs and PBMCs
- METHOD DETAILS
  - Media and reagents
  - Lentiviral vectors
  - Antibodies and flow cytometry
  - Enzyme-linked immunosorbent cytokine assays (ELISA)
  - Generation of allogeneic HSPC-engineered ESO-T (^{Allo}ESO-T) cells and their IL-15-armed derivatives (denoted as $^{allo/15}$ESO-T cells)
  - Generation and analysis of allogeneic HSPC-engineered HLA-ablated universal T (^{U}ESO-T) cells
  - Generation of PBMC-derived NK cells and PBMC-derived ESO-T (^{PBMC}ESO-T) cells
  - *In vitro* tumor cell killing assay
  - *In vitro* mixed lymphocyte reaction (MLR) for studying GvH response
  - *In vitro* MLR assay for studying T cell-mediated allorejection
  - *In vitro* MLR assay for studying NK cell-mediated allorejection
  - *In vitro* ganciclovir (GCV) treatment assay
  - *In vivo* bioluminescence imaging (BLI)
  - *In vivo* antitumor efficacy study of ^{Allo15}ESO-T cells: Orthotopic human OVCAR3 xenograft NSG mouse model
  - *In vivo* antitumor efficacy study of ^{Allo15}ESO-T cells: Metastatic human A375 xenograft NSG mouse model
  - *In vivo* PK-PD study of ^{Allo15}ESO-T/FG cells: Orthotopic human OVCAR3 xenograft NSG mouse model
  - *In vivo* PK-PD study of ^{Allo15}ESO-T/FG cells: Metastatic human A375 xenograft NSG mouse model
  - *In vivo* NKG2A immune checkpoint blockade study of ^{Allo15}ESO-T cells: Heavy tumor load human A375 xenograft NSG mouse model
  - *In vivo* CRS study
  - Single-cell TCR sequencing
  - Single-cell RNA sequencing (scRNA-seq)
  - Histology
- QUANTIFICATION AND STATISTICAL ANALYSIS

CellPress
OPEN ACCESS

SUPPLEMENTAL INFORMATION

Supplemental information can be found online at https://doi.org/10.1016/j.xcrm.2026.102998.

Received: December 2, 2025
Revised: April 20, 2026
Accepted: July 24, 2026
Published: August 19, 2026

REFERENCES

1. June, C.H. (2007). Adoptive T cell therapy for cancer in the clinic. J. Clin. Investig. *117*, 1466–1476. https://doi.org/10.1172/jci32446.
2. Ellis, G.I., Sheppard, N.C., and Riley, J.L. (2021). Genetic engineering of T cells for immunotherapy. Nat. Rev. Genet. *22*, 427–447. https://doi.org/10.1038/s41576-021-00329-9.
3. Fesnak, A.D., June, C.H., and Levine, B.L. (2016). Engineered T cells: the promise and challenges of cancer immunotherapy. Nat. Rev. Cancer *16*, 566–581. https://doi.org/10.1038/nrc.2016.97.
4. Baulu, E., Gardet, C., Chuvin, N., and Depil, S. (2023). TCR-engineered T cell therapy in solid tumors: State of the art and perspectives. Sci. Adv. *9*, eadf3700. https://doi.org/10.1126/sciadv.adf3700.
5. Van Der Merwe, P.A., and Dushek, O. (2011). Mechanisms for T cell receptor triggering. Nat. Rev. Immunol. *11*, 47–55. https://doi.org/10.1038/nri2887.
6. Gjerstorff, M.F., Andersen, M.H., and Ditzel, H.J. (2015). Oncogenic cancer/testis antigens: prime candidates for immunotherapy. Oncotarget *6*, 15772–15787. https://doi.org/10.18632/oncotarget.4694.
7. Fratta, E., Coral, S., Covre, A., Parisi, G., Colizzi, F., Danielli, R., Marie Nicolay, H.J., Sigalotti, L., and Maio, M. (2011). The biology of cancer testis antigens: putative function, regulation and therapeutic potential. Mol. Oncol. *5*, 164–182. https://doi.org/10.1016/j.molonc.2011.02.001.
8. Kulkarni, P., Shiraishi, T., Rajagopalan, K., Kim, R., Mooney, S.M., and Getzenberg, R.H. (2012). Cancer/testis antigens and urological malignancies. Nat. Rev. Urol. *9*, 386–396. https://doi.org/10.1038/nrurol.2012.117.
9. Mullard, A. (2024). FDA approves first TCR-engineered T cell therapy, for rare soft-tissue cancer. Nat. Rev. Drug Discov. *23*, 731.
10. Thomas, R., Al-Khadairi, G., Roelands, J., Hendrickx, W., Dermime, S., Bedognetti, D., and Decock, J. (2018). NY-ESO-1 based immunotherapy of cancer: current perspectives. Front. Immunol. *9*, 947. https://doi.org/10.3389/fimmu.2018.00947.
11. Goydos, J.S., Patel, M., and Shih, W. (2001). NY-ESO-1 and CTp11 expression may correlate with stage of progression in melanoma. J. Surg. Res. *98*, 76–80. https://doi.org/10.1006/jsre.2001.6148.
12. Aung, P.P., Liu, Y.-C., Ballester, L.Y., Robbins, P.F., Rosenberg, S.A., and Lee, C.-C.R. (2014). Expression of New York esophageal squamous cell carcinoma-1 in primary and metastatic melanoma. Hum. Pathol. *45*, 259–267. https://doi.org/10.1016/j.humpath.2013.05.029.
13. Sharma, P., Gnjatic, S., Jungbluth, A.A., Williamson, B., Herr, H., Stockert, E., Dalbagni, G., Donat, S.M., Reuter, V.E., Santiago, D., et al. (2003). Frequency of NY-ESO-1 and LAGE-1 expression in bladder cancer and evidence of a new NY-ESO-1 T-cell epitope in a patient with bladder cancer. Cancer Immun. *3*, 19.
14. Raza, A., Merhi, M., Inchakalody, V.P., Krishnankutty, R., Relecom, A., Uddin, S., and Dermime, S. (2020). Unleashing the immune response to NY-ESO-1 cancer testis antigen as a potential target for cancer immunotherapy. J. Transl. Med. *18*, 140. https://doi.org/10.1186/s12967-020-02306-y.
15. Zhao, R.Y., Mifsud, N.A., Xiao, K., Chan, K.-F., Oveissi, S., Jackson, H.M., Dimopoulos, N., Guillaume, P., Knights, A.J., and Lowen, T. (2012). A Novel HLA-B18 Restricted CD8+ T Cell Epitope Is Efficiently Cross-Presented by Dendritic Cells from Soluble Tumor Antigen. PLoS One *7*, e44707. https://doi.org/10.1371/journal.pone.0044707.
16. Ademuyiwa, F.O., Bshara, W., Attwood, K., Morrison, C., Edge, S.B., Karpf, A.R., O'Connor, T.L., Ambrosone, C.B., O'Connor, T.L., Levine, E.G., et al. (2012). NY-ESO-1 cancer testis antigen demonstrates high immunogenicity in triple negative breast cancer. PLoS One *7*, e38783. https://doi.org/10.1371/journal.pone.0038783.
17. Bethune, M.T., Li, X.-H., Yu, J., McLaughlin, J., Cheng, D., Mathis, C., Moreno, B.H., Woods, K., Knights, A.J., Garcia-Diaz, A., et al. (2018). Isolation and characterization of NY-ESO-1-specific T cell receptors restricted on various MHC molecules. Proc. Natl. Acad. Sci. USA *115*, E10702–E10711. https://doi.org/10.1073/pnas.1810653115.
18. Kawai, A., Ishihara, M., Nakamura, T., Kitano, S., Iwata, S., Takada, K., Emori, M., Kato, K., Endo, M., Matsumoto, Y., et al. (2023). Safety and efficacy of NY-ESO-1 antigen-specific T-cell receptor gene-transduced T lymphocytes in patients with synovial sarcoma: a phase I/II clinical trial. Clin. Cancer Res. *29*, 5069–5078. https://doi.org/10.1158/1078-0432.CCR-23-1456.
19. Pan, Q., Weng, D., Liu, J., Han, Z., Ou, Y., Xu, B., Peng, R., Que, Y., Wen, X., Yang, J., et al. (2023). Phase 1 clinical trial to assess safety and efficacy of NY-ESO-1-specific TCR T cells in HLA-A* 02: 01 patients with advanced soft tissue sarcoma. Cell Rep. Med. *4*, 101133. https://doi.org/10.1016/j.xcrm.2023.101133.
20. Odunsi, K., Cristea, M.C., Dorigo, O., Jazaeri, A.A., Slomovitz, B.M., Chagin, K., Van Winkle, E., Kari, G., Iyengar, M., and Norry, E. (2017). A Phase I/IIa, Open Label, Clinical Trial Evaluating the Safety and Efficacy of Autologous T Cells Expressing Enhanced TCRs Specific for NY-ESO-1 in Patients with Recurrent or Treatment Refractory Ovarian Cancer (NCT01567891) (American Society of Clinical Oncology).
21. Rapoport, A.P., Stadtmauer, E.A., Binder-Scholl, G.K., Goloubeva, O., Vogl, D.T., Lacey, S.F., Badros, A.Z., Garfall, A., Weiss, B., Finklestein, J., et al. (2015). NY-ESO-1–specific TCR–engineered T cells mediate sustained antigen-specific antitumor effects in myeloma. Nat. Med. *21*, 914–921. https://doi.org/10.1038/nm.3910.
22. Robbins, P.F., Morgan, R.A., Feldman, S.A., Yang, J.C., Sherry, R.M., Dudley, M.E., Wunderlich, J.R., Nahvi, A.V., Helman, L.J., Mackall, C.L., et al. (2011). Tumor regression in patients with metastatic synovial cell sarcoma and melanoma using genetically engineered lymphocytes reactive with NY-ESO-1. J. Clin. Oncol. *29*, 917–924. https://doi.org/10.1200/JCO.2010.32.2537.
23. Giraldo, N.A., Sanchez-Salas, R., Peske, J.D., Vano, Y., Becht, E., Petitprez, F., Validire, P., Ingels, A., Cathelineau, X., Fridman, W.H., and Sautès-Fridman, C. (2019). The clinical role of the TME in solid cancer. Br. J. Cancer *120*, 45–53. https://doi.org/10.1038/s41416-018-0327-z.
24. Zhu, Y., Kang, E., Wilson, M., Basso, T., Chen, E., Yu, Y., and Li, Y.-R. (2022). 3D tumor spheroid and organoid to model tumor microenvironment for cancer immunotherapy. Organoids *1*, 149–167. https://doi.org/10.3390/organoids1020012.
25. Klebanoff, C.A., Rosenberg, S.A., and Restifo, N.P. (2016). Prospects for gene-engineered T cell immunotherapy for solid cancers. Nat. Med. *22*, 26–36. https://doi.org/10.1038/nm.4015.
26. Tahmasebi, S., Elahi, R., and Esmaeilzadeh, A. (2019). Solid tumors challenges and new insights of CAR T cell engineering. Stem Cell Rev. Rep. *15*, 619–636. https://doi.org/10.1007/s12015-019-09901-7.
27. Kunert, A., Straetemans, T., Govers, C., Lamers, C., Mathijssen, R., Sleijfer, S., and Debets, R. (2013). TCR-engineered T cells meet new challenges to treat solid tumors: choice of antigen, T cell fitness, and sensitization of tumor milieu. Front. Immunol. *4*, 363. https://doi.org/10.3389/fimmu.2013.00363.
28. Belli, C., Trapani, D., Viale, G., D'Amico, P., Duso, B.A., Della Vigna, P., Orsi, F., and Curigliano, G. (2018). Targeting the microenvironment in solid tumors. Cancer Treat Rev. *65*, 22–32. https://doi.org/10.1016/j.ctrv.2018.02.004.

29. Trédan, O., Galmarini, C.M., Patel, K., and Tannock, I.F. (2007). Drug resistance and the solid tumor microenvironment. JNCI Journal of the National Cancer Institute *99*, 1441–1454. https://doi.org/10.1093/jnci/djm135.

30. Aftab, B.T., Sasu, B., Krishnamurthy, J., Gschweng, E., Alcazer, V., and Depil, S. (2020). Toward "off-the-shelf" allogeneic CAR T cells. Adv. Cell Gene Ther. *3*, e86. https://doi.org/10.1002/acg2.86.

31. Diorio, C., Teachey, D.T., and Grupp, S.A. (2025). Allogeneic chimeric antigen receptor cell therapies for cancer: progress made and remaining roadblocks. Nat. Rev. Clin. Oncol. *22*, 10–27. https://doi.org/10.1038/s41571-024-00959-y.

32. Depil, S., Duchateau, P., Grupp, S.A., Mufti, G., and Poirot, L. (2020). 'Off-the-shelf'allogeneic CAR T cells: development and challenges. Nat. Rev. Drug Discov. *19*, 185–199. https://doi.org/10.1038/s41573-019-0051-2.

33. Ishihara, M., Kitano, S., Kageyama, S., Miyahara, Y., Yamamoto, N., Kato, H., Mishima, H., Hattori, H., Funakoshi, T., Kojima, T., et al. (2022). NY-ESO-1-specific redirected T cells with endogenous TCR knockdown mediate tumor response and cytokine release syndrome. J. Immunother. Cancer *10*, e003811. https://doi.org/10.1136/jitc-2021-003811.

34. Bailey, S.R., and Maus, M.V. (2019). Gene editing for immune cell therapies. Nat. Biotechnol. *37*, 1425–1434. https://doi.org/10.1038/s41587-019-0137-8.

35. Wang, X., and Rivière, I. (2016). Clinical manufacturing of CAR T cells: foundation of a promising therapy. Mol. Ther. Oncolytics *3*, 16015. https://doi.org/10.1038/mto.2016.15.

36. Zhang, X., Jin, X., Sun, R., Zhang, M., Lu, W., and Zhao, M. (2022). Gene knockout in cellular immunotherapy: Application and limitations. Cancer Lett. *540*, 215736. https://doi.org/10.1016/j.canlet.2022.215736.

37. Abou-el-Enein, M., Elsallab, M., Feldman, S.A., Fesnak, A.D., Heslop, H.E., Marks, P., Till, B.G., Bauer, G., and Savoldo, B. (2021). Scalable manufacturing of CAR T cells for cancer immunotherapy. Blood Cancer Discov. *2*, 408–422. https://doi.org/10.1158/2643-3230.BCD-21-0084.

38. Li, Y.-R., Zhou, Y., Yu, J., Kim, Y.J., Li, M., Lee, D., Zhou, K., Chen, Y., Zhu, Y., Wang, Y.-C., et al. (2025). Generation of allogeneic CAR-NKT cells from hematopoietic stem and progenitor cells using a clinically guided culture method. Nat. Biotechnol. *43*, 329–344. https://doi.org/10.1038/s41587-024-02226-y.

39. Nishimura, T., Kaneko, S., Kawana-Tachikawa, A., Tajima, Y., Goto, H., Zhu, D., Nakayama-Hosoya, K., Iriguchi, S., Uemura, Y., Shimizu, T., et al. (2013). Generation of rejuvenated antigen-specific T cells by reprogramming to pluripotency and redifferentiation. Cell Stem Cell *12*, 114–126. https://doi.org/10.1016/j.stem.2012.11.002.

40. Themeli, M., Kloss, C.C., Ciriello, G., Fedorov, V.D., Perna, F., Gonen, M., and Sadelain, M. (2013). Generation of tumor-targeted human T lymphocytes from induced pluripotent stem cells for cancer therapy. Nat. Biotechnol. *31*, 928–933. https://doi.org/10.1038/nbt.2678.

41. Seet, C.S., He, C., Bethune, M.T., Li, S., Chick, B., Gschweng, E.H., Zhu, Y., Kim, K., Kohn, D.B., Baltimore, D., et al. (2017). Generation of mature T cells from human hematopoietic stem and progenitor cells in artificial thymic organoids. Nat. Methods *14*, 521–530. https://doi.org/10.1038/nmeth.4237.

42. Cichocki, F., Bjordahl, R., Gaidarova, S., Mahmood, S., Abujarour, R., Wang, H., Tuininga, K., Felices, M., Davis, Z.B., Bendzick, L., et al. (2020). iPSC-derived NK cells maintain high cytotoxicity and enhance in vivo tumor control in concert with T cells and anti–PD-1 therapy. Sci. Transl. Med. *12*, eaaz5618. https://doi.org/10.1126/scitranslmed.aaz5618.

43. Krenciute, G., Prinzing, B.L., Yi, Z., Wu, M.-F., Liu, H., Dotti, G., Balyasnikova, I.V., and Gottschalk, S. (2017). Transgenic expression of IL15 improves antiglioma activity of IL13Rα2-CAR T cells but results in antigen loss variants. Cancer Immunol. Res. *5*, 571–581. https://doi.org/10.1158/2326-6066.cir-16-0376.

44. Hurton, L.V., Singh, H., Najjar, A.M., Switzer, K.C., Mi, T., Maiti, S., Olivares, S., Rabinovich, B., Huls, H., Forget, M.-A., et al. (2016). Tethered IL-15 augments antitumor activity and promotes a stem-cell memory subset in tumor-specific T cells. Proc. Natl. Acad. Sci. USA *113*, E7788–E7797. https://doi.org/10.1073/pnas.1610544113.

45. Xu, X., Huang, W., Heczey, A., Liu, D., Guo, L., Wood, M., Jin, J., Courtney, A.N., Liu, B., and Di Pierro, E.J. (2019). NKT cells coexpressing a GD2-specific chimeric antigen receptor and IL15 show enhancedin vivo persistence and antitumor activity against neuroblastoma. Clin. Cancer Res. 25, 7126.

46. Steffin, D., Ghatwai, N., Montalbano, A., Rathi, P., Courtney, A.N., Arnett, A.B., Fleurence, J., Sweidan, R., Wang, T., Zhang, H., et al. (2025). Interleukin-15-armoured GPC3 CAR T cells for patients with solid cancers. Nature *637*, 940–946. https://doi.org/10.1038/s41586-024-08261-8.

47. Heczey, A., Courtney, A.N., Montalbano, A., Robinson, S., Liu, K., Li, M., Ghatwai, N., Dakhova, O., Liu, B., Raveh-Sadka, T., et al. (2020). Anti-GD2 CAR-NKT cells in patients with relapsed or refractory neuroblastoma: an interim analysis. Nat. Med. *26*, 1686–1690. https://doi.org/10.1038/s41591-020-1074-2.

48. Li, Y.-R., Zhu, Y., and Yang, L. (2025). IL-15 in CAR engineering: striking an efficacy–safety balance. Trends Mol. Med. *31*, 977–981. https://doi.org/10.1016/j.molmed.2025.05.014.

49. Li, Y.-R., Zhou, Y., Kramer, A., and Yang, L. (2021). Engineering stem cells for cancer immunotherapy. Trends Cancer *7*, 1059–1073. https://doi.org/10.1016/j.trecan.2021.08.004.

50. Giannoni, F., Hardee, C.L., Wherley, J., Gschweng, E., Senadheera, S., Kaufman, M.L., Chan, R., Bahner, I., Gersuk, V., Wang, X., et al. (2013). Allelic exclusion and peripheral reconstitution by TCR transgenic T cells arising from transduced human hematopoietic stem/progenitor cells. Mol. Ther. *21*, 1044–1054. https://doi.org/10.1038/mt.2013.8.

51. Li, Y.-R., Dunn, Z.S., Yu, Y., Li, M., Wang, P., and Yang, L. (2023). Advancing cell-based cancer immunotherapy through stem cell engineering. Cell Stem Cell *30*, 592–610. https://doi.org/10.1016/j.stem.2023.02.009.

52. Chen, Y., Zhu, Y., Kramer, A., Fang, Y., Wilson, M., Li, Y.-R., and Yang, L. (2023). Genetic engineering strategies to enhance antitumor reactivity and reduce alloreactivity for allogeneic cell-based cancer therapy. Front. Med. *10*, 1135468. https://doi.org/10.3389/fmed.2023.1135468.

53. Liu, Y., Yan, X., Zhang, F., Zhang, X., Tang, F., Han, Z., and Li, Y. (2021). TCR-T immunotherapy: the challenges and solutions. Front. Oncol. *11*, 794183. https://doi.org/10.3389/fonc.2021.794183.

54. Bendle, G.M., Linnemann, C., Hooijkaas, A.I., Bies, L., de Witte, M.A., Jorritsma, A., Kaiser, A.D.M., Pouw, N., Debets, R., Kieback, E., et al. (2010). Lethal graft-versus-host disease in mouse models of T cell receptor gene therapy. Nat. Med. *16*, 565–570. https://doi.org/10.1038/nm.2128.

55. Provasi, E., Genovese, P., Lombardo, A., Magnani, Z., Liu, P.-Q., Reik, A., Chu, V., Paschon, D.E., Zhang, L., Kuball, J., et al. (2012). Editing T cell specificity towards leukemia by zinc finger nucleases and lentiviral gene transfer. Nat. Med. *18*, 807–815. https://doi.org/10.1038/nm.2700.

56. Bonini, C., Chapuis, A.G., Hudecek, M., Guedan, S., Magnani, C., Qasim, W., ESGCT, I., and of, C.A.R.T.C.S.C. (2023). Genome editing in engineered T cells for cancer immunotherapy. Hum. Gene Ther. *34*, 853–869. https://doi.org/10.1089/hum.2023.128.

57. Wang, W., Liu, Y., He, Z., Li, L., Liu, S., Jiang, M., Zhao, B., Deng, M., Wang, W., Mi, X., et al. (2024). Breakthrough of solid tumor treatment: CAR-NK immunotherapy. Cell Death Discov. *10*, 40. https://doi.org/10.1038/s41420-024-01815-9.

58. Li, Y.-R., Halladay, T., and Yang, L. (2024). Immune evasion in cell-based immunotherapy: unraveling challenges and novel strategies. J. Biomed. Sci. *31*, 5. https://doi.org/10.1186/s12929-024-00998-8.

59. Martinez, M., and Moon, E.K. (2019). CAR T cells for solid tumors: new strategies for finding, infiltrating, and surviving in the tumor microenvironment. Front. Immunol. *10*, 128. https://doi.org/10.3389/fimmu.2019.00128.

60. Hou, A.J., Chen, L.C., and Chen, Y.Y. (2021). Navigating CAR-T cells through the solid-tumour microenvironment. Nat. Rev. Drug Discov. *20*, 531–550. https://doi.org/10.1038/s41573-021-00189-2.

61. Morton, L.T., Wachsmann, T.L.A., Meeuwsen, M.H., Wouters, A.K., Remst, D.F.G., van Loenen, M.M., Falkenburg, J.H.F., and Heemskerk, M.H.M. (2022). T cell receptor engineering of primary NK cells to therapeutically target tumors and tumor immune evasion. J. Immunother. Cancer *10*, e003715. https://doi.org/10.1136/jitc-2021-003715.

62. Matsuzaki, J., Gnjatic, S., Mhawech-Fauceglia, P., Beck, A., Miller, A., Tsuji, T., Eppolito, C., Qian, F., Lele, S., Shrikant, P., et al. (2010). Tumor-infiltrating NY-ESO-1–specific CD8+ T cells are negatively regulated by LAG-3 and PD-1 in human ovarian cancer. Proc. Natl. Acad. Sci. USA *107*, 7875–7880. https://doi.org/10.1073/pnas.1003345107.

63. Szender, J.B., Papanicolau-Sengos, A., Eng, K.H., Miliotto, A.J., Lugade, A.A., Gnjatic, S., Matsuzaki, J., Morrison, C.D., and Odunsi, K. (2017). NY-ESO-1 expression predicts an aggressive phenotype of ovarian cancer. Gynecol. Oncol. *145*, 420–425. https://doi.org/10.1016/j.ygyno.2017.03.509.

64. Li, Y.-R., Fang, Y., Niu, S., Zhu, Y., Chen, Y., Lyu, Z., Zhu, E., Tian, Y., Huang, J., Rezek, V., et al. (2025). Allogeneic CD33-directed CAR-NKT cells for the treatment of bone marrow-resident myeloid malignancies. Nat. Commun. *16*, 1248. https://doi.org/10.1038/s41467-025-56270-6.

65. Li, Y.-R., Zeng, S., Dunn, Z.S., Zhou, Y., Li, Z., Yu, J., Wang, Y.-C., Ku, J., Cook, N., Kramer, A., and Yang, L. (2022). Off-the-shelf third-party HSC-engineered iNKT cells for ameliorating GvHD while preserving GvL effect in the treatment of blood cancers. iScience *25*, 104859. https://doi.org/10.1016/j.isci.2022.104859.

66. Sommer, C., Boldajipour, B., Kuo, T.C., Bentley, T., Sutton, J., Chen, A., Geng, T., Dong, H., Galetto, R., Valton, J., et al. (2019). Preclinical evaluation of allogeneic CAR T cells targeting BCMA for the treatment of multiple myeloma. Mol. Ther. *27*, 1126–1138. https://doi.org/10.1016/j.ymthe.2019.04.001.

67. Li, Y.-R., Zhou, Y., Kim, Y.J., Zhu, Y., Ma, F., Yu, J., Wang, Y.-C., Chen, X., Li, Z., Zeng, S., et al. (2021). Development of allogeneic HSC-engineered iNKT cells for off-the-shelf cancer immunotherapy. Cell Rep. Med. *2*, 100449. https://doi.org/10.1016/j.xcrm.2021.100449.

68. Zheng, L., Qin, S., Si, W., Wang, A., Xing, B., Gao, R., Ren, X., Wang, L., Wu, X., Zhang, J., et al. (2021). Pan-cancer single-cell landscape of tumor-infiltrating T cells. Science *374*, abe6474. https://doi.org/10.1126/science.abe6474.

69. Li, H., van der Leun, A.M., Yofe, I., Lubling, Y., Gelbard-Solodkin, D., van Akkooi, A.C.J., van den Braber, M., Rozeman, E.A., Haanen, J.B.A.G., and Blank, C.U. (2019). Dysfunctional CD8 T cells form a proliferative, dynamically regulated compartment within human melanoma. Cell *176*, 775–789.

70. Bai, Z., Feng, B., McClory, S.E., de Oliveira, B.C., Diorio, C., Gregoire, C., Tao, B., Yang, L., Zhao, Z., Peng, L., et al. (2024). Single-cell CAR T atlas reveals type 2 function in 8-year leukaemia remission. Nature *634*, 702–711. https://doi.org/10.1038/s41586-024-07762-w.

71. Good, C.R., Aznar, M.A., Kuramitsu, S., Samareh, P., Agarwal, S., Donahue, G., Ishiyama, K., Wellhausen, N., Rennels, A.K., Ma, Y., et al. (2021). An NK-like CAR T cell transition in CAR T cell dysfunction. Cell *184*, 6081–6100.e26. https://doi.org/10.1016/j.cell.2021.11.016.

72. Haradhvala, N.J., Leick, M.B., Maurer, K., Gohil, S.H., Larson, R.C., Yao, N., Gallagher, K.M.E., Katsis, K., Frigault, M.J., Southard, J., et al. (2022). Distinct cellular dynamics associated with response to CAR-T therapy for refractory B cell lymphoma. Nat. Med. *28*, 1848–1859. https://doi.org/10.1038/s41591-022-01959-0.

73. Lee, S.-W., Choi, H.Y., Lee, G.-W., Kim, T., Cho, H.-J., Oh, I.-J., Song, S.Y., Yang, D.H., and Cho, J.-H. (2021). CD8+ TILs in NSCLC differentiate into TEMRA via a bifurcated trajectory: deciphering immunogenicity of tumor antigens. J. Immunother. Cancer *9*, e002709. https://doi.org/10.1136/jitc-2021-002709.

74. Iwahori, K., Shintani, Y., Funaki, S., Yamamoto, Y., Matsumoto, M., Yoshida, T., Morimoto-Okazawa, A., Kawashima, A., Sato, E., Gottschalk, S., et al. (2019). Peripheral T cell cytotoxicity predicts T cell function in the tumor microenvironment. Sci. Rep. *9*, 2636. https://doi.org/10.1038/s41598-019-39345-5.

75. Rahim, M.K., Okholm, T.L.H., Jones, K.B., McCarthy, E.E., Liu, C.C., Yee, J.L., Tamaki, S.J., Marquez, D.M., Tenvooren, I., Wai, K., et al. (2023). Dynamic CD8+ T cell responses to cancer immunotherapy in human regional lymph nodes are disrupted in metastatic lymph nodes. Cell *186*, 1127–1143.e18. https://doi.org/10.1016/j.cell.2023.02.021.

76. Zhang, L., Yu, X., Zheng, L., Zhang, Y., Li, Y., Fang, Q., Gao, R., Kang, B., Zhang, Q., Huang, J.Y., et al. (2018). Lineage tracking reveals dynamic relationships of T cells in colorectal cancer. Nature *564*, 268–272. https://doi.org/10.1038/s41586-018-0694-x.

77. Uslu, U., Castelli, S., and June, C.H. (2024). CAR T cell combination therapies to treat cancer. Cancer Cell *42*, 1319–1325. https://doi.org/10.1016/j.ccell.2024.07.002.

78. Lv, Y., Luo, X., Xie, Z., Qiu, J., Yang, J., Deng, Y., Long, R., Tang, G., Zhang, C., and Zuo, J. (2024). Prospects and challenges of CAR-T cell therapy combined with ICIs. Front. Oncol. *14*, 1368732. https://doi.org/10.3389/fonc.2024.1368732.

79. Creelan, B.C., and Antonia, S.J. (2019). The NKG2A immune checkpoint—a new direction in cancer immunotherapy. Nat. Rev. Clin. Oncol. *16*, 277–278. https://doi.org/10.1038/s41571-019-0182-8.

80. Wang, X., Xiong, H., and Ning, Z. (2022). Implications of NKG2A in immunity and immune-mediated diseases. Front. Immunol. *13*, 960852. https://doi.org/10.3389/fimmu.2022.960852.

81. van Montfoort, N., Borst, L., Korrer, M.J., Sluijter, M., Marijt, K.A., Santegoets, S.J., van Ham, V.J., Ehsan, I., Charoentong, P., André, P., et al. (2018). NKG2A blockade potentiates CD8 T cell immunity induced by cancer vaccines. Cell *175*, 1744–1755.e15. https://doi.org/10.1016/j.cell.2018.10.028.

82. Kamiya, T., Seow, S.V., Wong, D., Robinson, M., and Campana, D. (2019). Blocking expression of inhibitory receptor NKG2A overcomes tumor resistance to NK cells. J. Clin. Investig. *129*, 2094–2106. https://doi.org/10.1172/JCI123955.

83. André, P., Denis, C., Soulas, C., Bourbon-Caillet, C., Lopez, J., Arnoux, T., Bléry, M., Bonnafous, C., Gauthier, L., Morel, A., et al. (2018). Anti-NKG2A mAb is a checkpoint inhibitor that promotes anti-tumor immunity by unleashing both T and NK cells. Cell *175*, 1731–1743.e13. https://doi.org/10.1016/j.cell.2018.10.014.

84. Melero, I., Ochoa, M.C., Molina, C., Sanchez-Gregorio, S., Garasa, S., Luri-Rey, C., Hervas-Stubbs, S., Casares, N., Elizalde, E., Gomis, G., et al. (2023). Intratumoral co-injection of NK cells and NKG2A-neutralizing monoclonal antibodies. EMBO Mol. Med. *15*, e17804. https://doi.org/10.15252/emmm.202317804.

85. Van Hall, T., André, P., Horowitz, A., Ruan, D.F., Borst, L., Zerbib, R., Narni-Mancinelli, E., van Der Burg, S.H., and Vivier, E. (2019). Monalizumab: inhibiting the novel immune checkpoint NKG2A. J. Immunother. Cancer *7*, 263–268. https://doi.org/10.1186/s40425-019-0761-3.

86. Chang, Y.-J., Zhao, X.-Y., and Huang, X.-J. (2018). Strategies for enhancing and preserving anti-leukemia effects without aggravating graft-versus-host disease. Front. Immunol. *9*, 3041. https://doi.org/10.3389/fimmu.2018.03041.

87. Shlomchik, W.D. (2007). Graft-versus-host disease. Nat. Rev. Immunol. *7*, 340–352. https://doi.org/10.1038/nri2000.

88. Tugues, S., Amorim, A., Spath, S., Martin-Blondel, G., Schreiner, B., De Feo, D., Lutz, M., Guscetti, F., Apostolova, P., Haftmann, C., et al. (2018). Graft-versus-host disease, but not graft-versus-leukemia immunity, is mediated by GM-CSF–licensed myeloid cells. Sci. Transl. Med. *10*, eaat8410. https://doi.org/10.1126/scitranslmed.aat8410.

89. Braud, V.M., Allan, D.S.J., O'Callaghan, C.A., Söderström, K., D'Andrea, A., Ogg, G.S., Lazetic, S., Young, N.T., Bell, J.I., Phillips, J.H., et al.

(1998). HLA-E binds to natural killer cell receptors CD94/NKG2A, B and C. Nature *391*, 795–799. https://doi.org/10.1038/35869.

90. Lanza, R., Russell, D.W., and Nagy, A. (2019). Engineering universal cells that evade immune detection. Nat. Rev. Immunol. *19*, 723–733. https://doi.org/10.1038/s41577-019-0200-1.

91. Wagner, D.L., Fritsche, E., Pulsipher, M.A., Ahmed, N., Hamieh, M., Hegde, M., Ruella, M., Savoldo, B., Shah, N.N., Turtle, C.J., et al. (2021). Immunogenicity of CAR T cells in cancer therapy. Nat. Rev. Clin. Oncol. *18*, 379–393. https://doi.org/10.1038/s41571-021-00476-2.

92. Norelli, M., Camisa, B., Barbiera, G., Falcone, L., Purevdorj, A., Genua, M., Sanvito, F., Ponzoni, M., Doglioni, C., Cristofori, P., et al. (2018). Monocyte-derived IL-1 and IL-6 are differentially required for cytokine-release syndrome and neurotoxicity due to CAR T cells. Nat. Med. *24*, 739–748. https://doi.org/10.1038/s41591-018-0036-4.

93. Giavridis, T., van der Stegen, S.J.C., Eyquem, J., Hamieh, M., Piersigilli, A., and Sadelain, M. (2018). CAR T cell–induced cytokine release syndrome is mediated by macrophages and abated by IL-1 blockade. Nat. Med. *24*, 731–738. https://doi.org/10.1038/s41591-018-0041-7.

94. Ren, J., Liu, X., Fang, C., Jiang, S., June, C.H., and Zhao, Y. (2017). Multiplex genome editing to generate universal CAR T cells resistant to PD1 inhibition. Clin. Cancer Res. *23*, 2255–2266. https://doi.org/10.1158/1078-0432.CCR-16-1300.

95. Paul, S., and Lal, G. (2017). The molecular mechanism of natural killer cells function and its importance in cancer immunotherapy. Front. Immunol. *8*, 1124. https://doi.org/10.3389/fimmu.2017.01124.

96. Torikai, H., Reik, A., Soldner, F., Warren, E.H., Yuen, C., Zhou, Y., Crossland, D.L., Huls, H., Littman, N., Zhang, Z., et al. (2013). Toward eliminating HLA class I expression to generate universal cells from allogeneic donors. Blood *122*, 1341–1349. https://doi.org/10.1182/blood-2013-03-478255.

97. Hill, G.R., Betts, B.C., Tkachev, V., Kean, L.S., and Blazar, B.R. (2021). Current concepts and advances in graft-versus-host disease immunology. Annu. Rev. Immunol. *39*, 19–49. https://doi.org/10.1146/annurev-immunol-102119-073227.

98. Zemmour, D., Zilionis, R., Kiner, E., Klein, A.M., Mathis, D., and Benoist, C. (2018). Single-cell gene expression reveals a landscape of regulatory T cell phenotypes shaped by the TCR. Nat. Immunol. *19*, 291–301. https://doi.org/10.1038/s41590-018-0051-0.

99. Li, Y.-R., Zhou, Y., Yu, J., Zhu, Y., Lee, D., Zhu, E., Li, Z., Kim, Y.J., Zhou, K., Fang, Y., et al. (2024). Engineering allorejection-resistant CAR-NKT cells from hematopoietic stem cells for off-the-shelf cancer immunotherapy. Mol. Ther. *32*, 1849–1874. https://doi.org/10.1016/j.ymthe.2024.04.005.

100. Gschweng, E.H., McCracken, M.N., Kaufman, M.L., Ho, M., Hollis, R.P., Wang, X., Saini, N., Koya, R.C., Chodon, T., Ribas, A., et al. (2014). HSV-sr39TK positron emission tomography and suicide gene elimination of human hematopoietic stem cells and their progeny in humanized mice. Cancer Res. *74*, 5173–5183. https://doi.org/10.1158/0008-5472.CAN-14-0376.

101. Basar, R., Daher, M., and Rezvani, K. (2020). Next-generation cell therapies: the emerging role of CAR-NK cells. Hematology, 570–570578. https://doi.org/10.1182/hematology.2020002547.

102. Li, Y., Hermanson, D.L., Moriarity, B.S., and Kaufman, D.S. (2018). Human iPSC-derived natural killer cells engineered with chimeric antigen receptors enhance anti-tumor activity. Cell Stem Cell *23*, 181–192.e5. https://doi.org/10.1016/j.stem.2018.06.002.

103. Iriguchi, S., Yasui, Y., Kawai, Y., Arima, S., Kunitomo, M., Sato, T., Ueda, T., Minagawa, A., Mishima, Y., Yanagawa, N., et al. (2021). A clinically applicable and scalable method to regenerate T-cells from iPSCs for off-the-shelf T-cell immunotherapy. Nat. Commun. *12*, 430. https://doi.org/10.1038/s41467-020-20658-3.

104. Benjamin, R., Graham, C., Yallop, D., Jozwik, A., Ciocarlie, O., Jain, N., Jabbour, E.J., Maus, M.V., Frigault, M., Boissel, N., et al. (2018). Preliminary data on safety, cellular kinetics and anti-leukemic activity of UCART19, an allogeneic anti-CD19 CAR T-cell product, in a pool of adult and pediatric patients with high-risk CD19+ relapsed/refractory B-cell acute lymphoblastic leukemia. Blood *132*, 896. https://doi.org/10.1182/blood-2018-99-111356.

105. Benjamin, R., Graham, C., Yallop, D., Jozwik, A., Mirci-Danicar, O.C., Lucchini, G., Pinner, D., Jain, N., Kantarjian, H., Boissel, N., et al. (2020). Genome-edited, donor-derived allogeneic anti-CD19 chimeric antigen receptor T cells in paediatric and adult B-cell acute lymphoblastic leukaemia: results of two phase 1 studies. Lancet *396*, 1885–1894. https://doi.org/10.1016/S0140-6736(20)32334-5.

106. Stadtmauer, E.A., Fraietta, J.A., Davis, M.M., Cohen, A.D., Weber, K.L., Lancaster, E., Mangan, P.A., Kulikovskaya, I., Gupta, M., Chen, F., et al. (2020). CRISPR-engineered T cells in patients with refractory cancer. Science *367*, eaba7365. https://doi.org/10.1126/science.aba7365.

107. Qasim, W., Zhan, H., Samarasinghe, S., Adams, S., Amrolia, P., Stafford, S., Butler, K., Rivat, C., Wright, G., Somana, K., et al. (2017). Molecular remission of infant B-ALL after infusion of universal TALEN gene-edited CAR T cells. Sci. Transl. Med. *9*, eaaj2013. https://doi.org/10.1126/scitranslmed.aaj2013.

108. Themeli, M., Rivière, I., and Sadelain, M. (2015). New Cell Sources for T Cell Engineering and Adoptive Immunotherapy. Cell Stem Cell *16*, 357–366. https://doi.org/10.1016/j.stem.2015.03.011.

109. Hosking, M.P., Shirinbak, S., Omilusik, K., Chandra, S., Kaneko, M.K., Gentile, A., Yamamoto, S., Shrestha, B., Grant, J., Boyett, M., et al. (2025). Preferential tumor targeting of HER2 by iPSC-derived CAR T cells engineered to overcome multiple barriers to solid tumor efficacy. Cell Stem Cell *32*, 1087–1101.e4. https://doi.org/10.1016/j.stem.2025.05.007.

110. Jing, R., Falchetti, M., Han, T., Najia, M., Hensch, L., Meader, E., Kononov, M., Wang, S., Kubaczka, C., Lummertz Da Rocha, E., et al. (2024). Generation of Functional iPSC-Derived CAR-T Cells for Cancer Immunotherapy Via G9a/GLP Inhibition. Blood *144*, 2043. https://doi.org/10.1182/blood-2024-208634.

111. Niizuma, K., Nishimura, T., Villanueva, J., Amaya, L., Fowler, J.L., Isobe, T., Nakauchi, Y., Saavedra, B., Xu, H., Nakanishi, M., et al. (2025). Development of iPSC-derived T cells targeting EGFR neoantigens in non-small cell lung cancer. Mol. Ther. Methods Clin. Dev. *33*, 101517. https://doi.org/10.1016/j.omtm.2025.101517.

112. Nianias, A., and Themeli, M. (2019). Induced Pluripotent Stem Cell (iPSC)–Derived Lymphocytes for Adoptive Cell Immunotherapy: Recent Advances and Challenges. Curr. Hematol. Malig. Rep. *14*, 261–268. https://doi.org/10.1007/s11899-019-00528-6.

113. Vizcardo, R., Masuda, K., Yamada, D., Ikawa, T., Shimizu, K., Fujii, S.i., Koseki, H., and Kawamoto, H. (2013). Regeneration of human tumor antigen-specific T cells from iPSCs derived from mature CD8+ T cells. Cell Stem Cell *12*, 31–36. https://doi.org/10.1016/j.stem.2012.12.006.

114. Zhu, Y., Smith, D.J., Zhou, Y., Li, Y.-R., Yu, J., Lee, D., Wang, Y.-C., Di Biase, S., Wang, X., Hardoy, C., et al. (2019). Development of hematopoietic stem cell-engineered invariant natural killer T cell therapy for cancer. Cell Stem Cell *25*, 542–557.e9. https://doi.org/10.1016/j.stem.2019.08.004.

115. Smith, D.J., Liu, S., Ji, S., Li, B., McLaughlin, J., Cheng, D., Witte, O.N., and Yang, L. (2015). Genetic engineering of hematopoietic stem cells to generate invariant natural killer T cells. Proc. Natl. Acad. Sci. USA *112*, 1523–1528. https://doi.org/10.1073/pnas.1424877112.

116. Li, Y.-R., Zhu, Y., and Yang, L. (2025). IL-18 revives dysfunctional CAR-T cells. Trends Cancer *11*, 923–926. https://doi.org/10.1016/j.trecan.2025.06.008.

117. Li, Y.-R., Li, M., Chen, Y., Leng, H., Zhu, Y., Shen, X., Croll, D., Zhu, E., Huang, J., Wang, Y.-C., et al. (2026). Spatiotemporal profiling reveals distinct dynamics and checkpoint regulations of CAR-T and CAR-NKT cells against solid tumors. Signal Transduct. Target. Ther. *11*, 92. https://doi.org/10.1038/s41392-026-02602-x.

STAR★METHODS

KEY RESOURCES TABLE

REAGENT or RESOURCE	SOURCE	IDENTIFIER
Antibodies		
Anti-human TCRVβ13.1 (Clone H131)	BioLegend	CAT#362408, RRID: AB_2728349
Anti-human CD3 (clone HIT3a)	BioLegend	CAT#300329, RRID: AB_10551436
Anti-human CD4 (Clone OKT4)	BioLegend	CAT#317414, RRID: AB_571959
Anti-human CD8 (Clone SK1)	BioLegend	CAT#344714, RRID: AB_2044005
Anti-human CD34 (Clone 581)	BD Biosciences	CAT#555822, RRID: AB_396151
Anti-human CD45 (Clone HI30)	BioLegend	CAT#304026, RRID: AB_893341
Anti-human CD69 (Clone FN50)	BioLegend	CAT#310906, RRID: AB_314841
Anti-human CD94 (Clone DX22)	BioLegend	CAT#981102, RRID: AB_2133129
Anti-human NKG2A (Clone S19004C)	BioLegend	CAT#375103, RRID: AB_2888861
Anti-human NKG2C (Clone S19005E)	BioLegend	CAT#375003, RRID: AB_2888871
Anti-human NKG2D (Clone 1D11)	BioLegend	CAT#320812, RRID: AB_2234394
Anti-human DNAM-1 (Clone 11A8)	BioLegend	CAT#338312, RRID: AB_2561952
Anti-human NKp30 (Clone P30-15)	BioLegend	CAT#325207, RRID: AB_756112
Anti-human NKp44 (Clone P44-8)	BioLegend	CAT#325107, RRID: AB_756100
Anti-human IFN-γ (Clone B27)	BioLegend	CAT#506518, RRID: AB_2123321
Anti-human Granzyme B (Clone QA16A02)	BioLegend	CAT#372204, RRID: AB_2687028
Anti-human Perforin (Clone dG9)	BioLegend	CAT#308126, RRID: AB_2572049
Anti-human TNF-α (Clone MAb11)	BioLegend	CAT#502912, RRID: AB_315265
Anti-human IL-2 (Clone MQ1-17H12)	BioLegend	CAT#500341, RRID: AB_2562855
Anti-human β2-microglobulin (B2M) (Clone 2M2)	BioLegend	CAT#316304, RRID: AB_492837
Anti-human CTAG1B (Clone W19067B)	BioLegend	CAT#382302, RRID: AB_2922615
Anti-human PD-1 (Clone A17188A)	BioLegend	CAT#379210, RRID: AB_2922607
Anti-human TIM-3 (Clone A18087E)	BioLegend	CAT#364804, RRID: AB_2910409
Anti-human LAG-3 (Clone 7H2C65)	BioLegend	CAT#369208, RRID: AB_2629835
Purified mouse IgG2b, κ isotype control antibody (Clone MG2b-57)	BioLegend	CAT#401202, RRID: AB_2744505
Anti-human ULBP-1 (Clone 170818)	R&D Systems	CAT#FAB1380P, RRID: AB_2687471
HLA-A*02:01–NY-ESO-1 157–165 tetramer	NIH Tetramer Core Facility	RRID: SCR_026557
HLA-B*07:02–NY-ESO-1 60–72 tetramer	NIH Tetramer Core Facility	RRID: SCR_026557
Mouse Fc Block (anti-mouse CD16/32)	BD Biosciences	CAT#553142, RRID: AB_394656
Anti-human IFN-γ (ELISA, capture; Clone NIB42)	BD Biosciences	CAT#551221, RRID: AB_394099
Anti-human IFN-γ (ELISA, detection; Clone 4S.B3)	BD Biosciences	CAT#554550, RRID: AB_395472
Anti-human TNF-α (ELISA, capture; Clone MAb1)	BD Biosciences	CAT#551220, RRID: AB_394098
Anti-human TNF-α (ELISA, detection; Clone MAb11)	BD Biosciences	CAT#554511, RRID: AB_395442
Anti-mouse IL-6 (ELISA, capture; Clone MP5-20F3)	BD Biosciences	CAT#554400; RRID: AB_315336
Anti-mouse IL-6 (ELISA, detection; Clone MP5-32C11)	BD Biosciences	CAT#554402; RRID: AB_2127458

(Continued on next page)

Continued

REAGENT or RESOURCE	SOURCE	IDENTIFIER
Ultra-LEAF^{TM} Purified Anti-Human CD3 Antibody (Clone OKT3)	BioLegend	CAT#317347
Ultra-LEAF^{TM} Purified Anti-Human CD28 Antibody (Clone CD28.2)	BioLegend	CAT#302943
Ultra-LEAF^{TM} Purified Mouse IgG2b, κ Isotype Ctrl Antibody	BioLegend	CAT#401215
Ultra-LEAF^{TM} Purified anti-human CD226 (DNAM-1) Antibody	BioLegend	CAT#338302
Ultra-LEAF^{TM} Purified anti-human CD314 (NKG2D) Antibody (Clone 1D11)	Biolegend	CAT#320813
Monalizumab (anti-NKG2A)	MCE	CAT#HY-P99032
Bacterial and virus strains		
Lenti/1G4	This paper	N/A
Lenti/1G4-IL15	This paper	N/A
Lenti/1G5-HLAE-TK	This paper	N/A
Lenti/1E4	This paper	N/A
Lenti/HLA-A2	This paper	N/A
Lenti/FG	This paper	N/A
Biological samples		
Human peripheral blood mononuclear cells (PBMCs)	UCLA/CFAR Virology Core Laboratory	N/A
Cord blood CD34^{+} stem/progenitor cells	HemaCare	N/A
Chemicals, peptides, and recombinant proteins		
NY-ESO-1 peptide	IBA Lifesciences	CAT#6-7013-901
Streptavidin-HRP conjugate	Invitrogen	CAT#SA10001
Human IFN-γ (ELISA, standard)	eBioscience	CAT#29-8319-65
Human TNF-α (ELISA, standard)	eBioscience	CAT#29-8329-65
Mouse IL-6 (ELISA, standard)	eBioscience	CAT#29-8061-65
Tetramethylbenzidine (TMB)	KPL	CAT#5120-0053
Recombinant human IL-2	PeproTech	CAT#200-02
Recombinant human IL-3	PeproTech	CAT#200-03
Recombinant human IL-7	PeproTech	CAT#200-07
Recombinant human IL-15	PeproTech	CAT#200-15
Recombinant human Flt-3 ligand	PeproTech	CAT#300-19
Recombinant human SCF	PeproTech	CAT#300-07
Recombinant human TPO	PeproTech	CAT#300-18
Cas9-NLS purified protein	UC Berkeley	N/A
X-VIVO 15 Serum-free Hematopoietic Cell Medium	Lonza Bioscience	CAT#04-418Q
RPMI1640 cell culture medium	Thermo Fisher Scientific	CAT# MT10040CV
DMEM cell culture medium	Thermo Fisher Scientific	CAT# MT10013CV
Fetal Bovine Serum (FBS)	Sigma-Aldrich	CAT#F2442
beta-mercaptoethanol (β-ME)	Sigma-Aldrich	CAT#1610710
Ganciclovir (GCV)	Sigma-Aldrich	CAT#ADV465749843
MACS BSA stock solution	Miltenyi	CAT#130-091-376
30% BSA	Gemini	CAT#700-110-100
Penicillin-Streptomycin-Glutamine (P/S/G)	Gibco	CAT#10-378-016
MEM non-essential amino acids (NEAA)	Gibco	CAT#11140050
HEPES Buffer Solution	Gibco	CAT#15630080

(*Continued on next page*)

CellPress
OPEN ACCESS

Continued

REAGENT or RESOURCE	SOURCE	IDENTIFIER
Sodium Pyruvate	Gibco	CAT#11360070
Normocin	InvivoGen	CAT#ant-nr-2
Fixable Viability Dye eFluor506	eBioscience	CAT#65-0866-14
Cell Fixation/Permeabilization Kit	BD Biosciences	CAT#554714
10% neutral-buffered formalin	Richard-Allan Scientific	CAT#5705
D-Luciferin Potassium Salt Bioluminescent Substrate	Revivi	CAT#122799-100MG
Fluriso (Isoflurane)	Zoetis	CAT#50019100
Phosphate Buffered Saline (PBS) pH 7.4 (1X)	Gibco	CAT#10010-023
Poloxamer Synperonic F 108	MilliporeSigma	CAT#07579-250G-F
Prostaglandin E2 (PGE2)	Cayman Chemical	CAT#14010
CryoStor ® Cell Cryopreservation Media CS10	MilliporeSigma	CAT#C2874
Critical commercial assays		
Human NK Cell Isolation Kit	Miltenyi Biotec	CAT#130-092-657
Human CD34 MicroBeads Kit	Miltenyi Biotec	CAT#130-046-703
Fixation/Permeabilization Solution Kit	BD Sciences	CAT#55474
Dynabeads™ Human T-Activator CD3/CD28	Thermo Fisher Scientific	CAT#11131D
StemSpan™ T cell Generation Kit	STEMCELL Technologies	CAT#09940
CTS™ OpTmizer™ T cell Expansion SFM, no phenol red, bottle format	Thermo Fisher Scientific	CAT#A3705001
TransIT-Lenti Transfection Reagent	Mirus Bio	CAT#MIR 6600
Amicon Ultra-15 Centrifugal Filter Unit	MilliporeSigma	CAT#UFC910024
Human IL-15 Quantikine ELISA Kit	R&D Systems	CAT#D1500
Mouse SAA-3 ELISA Kit	MilliporeSigma	CAT#EZMSAA3-12K
Deposited data		
Single cell RNA sequencing	This paper	Gene Expression Omnibus (GEO) Database: GSE301248
Experimental models: Cell lines		
Human ovarian cancer cell line OCVAR3	ATCC	CAT#HTB-161, RRID: CVCL_0465
Human melanoma cell line A375	ATCC	CAT#CRL-1619, RRID: CVCL_0132
Human prostate cancer cell line PC3	ATCC	CAT#CRL_1435, RRID: CVCL_0035
Human ovarian cancer cell line OVCAR8	NIH	RRID: CVCL_1629
Human artificial APC cell line	This paper	N/A
Human ovarian cancer cell line OCVAR3-FG	This paper	N/A
Human melanoma cell line A375-FG	This paper	N/A
Human prostate cancer cell line PC3-FG	This paper	N/A
Human prostate cancer cell line PC3-A2-FG	This paper	N.A
Human ovarian cancer cell line OVCAR8-FG	This paper	N/A
Experimental models: Organisms/strains		
NOD.Cg-*Prkdc*^{SCID} *Il2rg*^{tm1Wjl}/SzJ (NSG)	The Jackson Laboratory	Strain #005557
Oligonucleotides		
gRNA (*B2M*): CGCGAGCACAGCUAAGGCCA	Synthego	N/A
gRNA (*CIITA*): GAUAUUGGCAUAAGCCUCCC	Synthego	N/A

(*Continued on next page*)

Continued		
REAGENT or RESOURCE	SOURCE	IDENTIFIER
Recombinant DNA		
Vector: parental lentivector pMNDW	N/A	N/A
Software and algorithms		
FlowJo Software	FlowJo	https://www.flowjo.com/solutions/flowjo/downloads
AURA imaging software	Spectral Instruments Imaging	https://spectralinvivo.com/software/
Optronics PictureFrame software	AU Optronics	https://paperzz.com/doc/8301032/optronics–pictureframe
ImageJ	ImageJ	https://imagej.net/Downloads
Prism 9	Graphpad	https://www.graphpad.com/scientific-software/prism/
MATLAB	The MathWorks, Inc	http://www.mathworks.com/products/matlab.html
R	R	http://www.R-project.org/
RStudio	RStudio	https://posit.co

EXPERIMENTAL MODEL AND STUDY PARTICIPANT DETAILS

Mice

NOD.Cg-Prkdc^{SCID}Il2rg^{tm1Wjl}/SzJ (NOD/SCID/IL-2R$\gamma^{-/-}$, NSG; RRID: IMSR_JAX:005557) mice were maintained in the animal facilities at the UCLA. Male or female mice aged 6–10 weeks were used for all experiments unless otherwise specified. Sex was not considered in the study design and analysis, as no significant differences were observed in the human NSG mouse models used. All procedures involving animals were approved by the Institutional Animal Care and Use Committee (IACUC) of UCLA. Mice were bred and maintained under specific pathogen-free conditions, and all experimental protocols adhered to the guidelines established by the Division of Laboratory Animal Medicine (DLAM) at UCLA (ARC protocol: ARC-2013-054). Experimental mice were randomly assigned to treatment groups to avoid statistically significant differences in the baseline tumor burden.

Cell lines

Human ovarian cancer cell lines OVCAR3 (cat. no. HTB-161; RRID: CVCL_0465), melanoma cell line A375 (cat. no. CRL-1619; RRID: CVCL_0132), and prostate cancer cell line PC3 (cat. no. CRL_1435; RRID: CVCL_0035) were obtained from the American Type Culture Collection (ATCC). The human ovarian cancer cell line OVCAR8 was obtained from the National Institutes of Health (NIH; RRID: CVCL_1629). All tumor cell lines used in this study were tested mycoplasma-free and frequently STR verified during experiments.

To generate stable tumor cell lines expressing FG dual reporters (comprising firefly luciferase (Fluc) and enhanced green fluorescent protein (EGFP)), parental cell lines were transduced with the Lenti/FlucGFP vector. 72 h post-transduction, cells were sorted by flow cytometry to isolate gene-engineered populations and establish stable lines, including OVCAR3-FG, OVCAR8-FG, A375-FG, and PC3-FG. The PC3-A2-FG line was generated by further transducing PC3-FG cells with the Lenti/A2 vector, followed by the same transduction and sorting procedure.

Human CD34$^+$ HSPCs and PBMCs

Purified cord-blood-derived human CD34$^+$ cells were purchased from HemaCare. Healthy donor PBMCs were obtained from the UCLA/CFAR Virology Core Laboratory without identification information under federal and state regulations. CD34$^+$ HSPCs and PBMCs were cryopreserved in Cryostor CS10 (Sigma St. Louis, MO, USA) using CoolCell (BioCision, Larkspur, CA, USA), and were frozen in liquid nitrogen for storage and to supply all experiments.

METHOD DETAILS

Media and reagents

X-VIVO 15 Serum-Free Hematopoietic Cell Medium (cat. no. 04-418Q) was obtained from Lonza. The StemSpan T cell Generation Kit (cat. no. 09940), which includes StemSpan SFEM II Medium, StemSpan Lymphoid Progenitor Expansion Supplement, StemSpan T cell Progenitor Maturation Supplement, StemSpan Lymphoid Progenitor Differentiation Coating Material, and ImmunoCult Human CD3/CD28/CD2 T cell Activator, was purchased from StemCell Technologies. CTS OpTmizer T cell Expansion SFM (no phenol red, bottle format, cat. no. A3705001), Roswell Park Memorial Institute (RPMI) 1640 medium (cat. no. MT10040CV), and Dulbecco's Modified Eagle Medium (DMEM, cat. no. MT10013CV) were purchased from Thermo Fisher Scientific. CryoStor CS10 Cell Cryopreservation Medium (cat. no. C2874) was obtained from MilliporeSigma.

NY-ESO-1 peptide (cat. no. 6-7013-901) was purchased from IBA Lifesciences. Recombinant human cytokines IL-2 (cat. no. 200-02), IL-3 (cat. no. 200-03), IL-7 (cat. no. 200-07), IL-15 (cat. no. 200-15), Flt3 ligand (Flt3L, cat. no. 300-19), stem cell factor (SCF, cat. no. 300-07), and thrombopoietin (TPO, cat. no. 300-18) were obtained from PeproTech. Ganciclovir (GCV), fetal bovine serum (FBS), and beta-mercaptoethanol (β-ME) (cat. no. 1610710) were purchased from Sigma-Aldrich. Penicillin-Streptomycin-Glutamine (P/S/G, cat. no. 10-378-016), MEM non-essential amino acids (NEAA, cat. no. 11140050), HEPES buffer solution (cat. no. 15630080), and sodium pyruvate (cat. no. 11360070) were obtained from Gibco. Normocin (cat. no. ant-nr-2) was purchased from InvivoGen.

Custom media were prepared as follows: C10 medium was made of RPMI 1640 supplemented with 10% (vol/vol) FBS, 1% (vol/vol) P/S/G, 1% (vol/vol) MEM non-essential amino acids, 10 mM HEPES, 1 mM sodium pyruvate, 0.05 mM β-ME, and 0.1 mg/mL normocin. D10 medium was made of DMEM supplemented with 10% (vol/vol) FBS, 1% (vol/vol) P/S/G, and 0.1 mg/mL normocin. R10 medium was made of RPMI 1640 supplemented with 10% (vol/vol) FBS, 1% (vol/vol) PSG, and 0.1 mg/mL normocin.

Lentiviral vectors

All lentiviral vectors used in this study were derived from the parental lentivector pMNDW.[50,115] To enable co-expression of multiple genes, self-cleaving 2A peptide sequences from foot-and-mouth disease virus (F2A), porcine teschovirus-1 (P2A), and thosea asigna virus (T2A) were utilized to link transgene components.

The Lenti/1G4 vector was constructed by inserting a synthetic bicistronic gene encoding human ESO TCRα-F2A-TCRβ (NY-ESO-1-specific ($ESO_{157-165}$) HLA-A2.01-restricted TCR Gene) into the pMNDW backbone. The Lenti/1G4-IL-15 vector was constructed by inserting a synthetic tricistronic gene encoding human ESO TCRα-F2A-TCRβ-P2A-IL15 into pMNDW. The Lenti/1E4 vector was constructed by inserting a synthetic bicistronic gene encoding human ESO TCRα-F2A-TCRβ (NY-ESO-1-specific (ESO_{60-72}) HLA-B7.02-restricted TCR Gene) into pMNDW. The Lenti/1G4-HLAE-TK was constructed by inserting a synthetic tetracistronic gene encoding human ESO TCRα-F2A-TCRβ-P2A-HLAE-T2A-sr39TK (NY-ESO-1-specific ($ESO_{157-165}$) HLA-A2.01-restricted TCR Gene) into pMNDW. The Lenti/FlucGFP (FG) vector was constructed by inserting into pMNDW a synthetic bicistronic gene encoding Fluc-P2A-EGFP. The Lenti/HLA-A2 vector was constructed by inserting a synthetic gene encoding human HLA-A2 into pMNDW.

All synthetic gene fragments were purchased from GenScript and Integrated DNA Technologies (IDT). Lentiviral particles were produced in human embryonic kidney 293T (HEK293T) cells (American Type Culture Collection, ATCC; RRID: CVCL_0063) using the *Trans*-IT-Lenti Transfection Reagent (Mirus Bio), following the manufacturer's protocol. Viral supernatants were centrifugally concentrated using Amicon Ultra Centrifugal Filter Units (MilliporeSigma) according to the manufacturer's instructions.

Antibodies and flow cytometry

Fluorochrome-conjugated antibodies specific for human CD45 (Clone HI30, cat. no. 304026, 1:500 dilution; RRID: AB_893341), CD3 (Clone HIT3a, cat. no. 300329, 1:500 dilution; RRID: AB_10551436), CD4 (Clone OKT4, cat. no. 317414, 1:400 dilution; RRID: AB_571959), CD8 (Clone SK1, cat. no. 344714, 1:300 dilution; RRID: AB_2044005), CD69 (Clone FN50, cat. no. 310906, 1:50 dilution, RRID: AB_314841), CD94 (Clone DX22, cat. No. 981102, 1:50 dilution; RRID: AB_2133129), NKG2A (Clone S19004C, cat. no. 375103, 1:50 dilution; RRID: AB_2888861), NKG2C (Clone S19005E, cat. no. 375003, 1:50 dilution; RRID: AB_2888871), NKG2D (Clone 1D11, cat. no. 320812, 1:50 dilution; RRID: AB_2234394), DNAM-1 (Clone 11A8, cat. no. 338312, 1:50 dilution; AB_2561952), NKp30 (Clone P30-15, cat. no. 325207, 1:50 dilution; RRID: AB_756112), NKp44 (Clone P44-8, cat. no. 325107, 1:50 dilution; RRID: AB_756100), IFN-γ (Clone B27, cat. no. 506518, 1:50 dilution; RRID: AB_2123321), Granzyme B (Clone QA16A02, cat. no. 372204, 1:2,000 dilution; RRID: AB_2687028), Perforin (Clone dG9, cat. no. 308126, 1:20 dilution; RRID: AB_2572049), TNF-α (Clone MAb11, cat. no. 502912, 1:500 dilution; RRID: AB_315265), IL-2 (Clone MQ1-17H12, cat. no. 500341, 1:20 dilution; RRID: AB_2562855), β2-microglobulin (B2M) (Clone 2M2, cat. no. 316304, 1:2000 dilution; RRID: AB_492837), TCRVβ13.1 (Clone HI31, cat. no. 362408, 1:1:100 dilution; RRID: AB_2728349), CTAG1B (Clone W19067B, cat. No. 382302, 1:500 dilution; AB_2922615), PD-1 (Clone A17188A, cat. no. 379210, 1:50 dilution; RRID: AB_2922607), TIM-3 (Clone A18087E, cat. no. 364804, 1:50 dilution; AB_2910409), and LAG-3 (Clone 7H2C65, cat. no. 369208, 1:50 dilution; RRID: AB_2629835) were purchased from BioLegend. Mouse lgG2b κ, Isotype control antibody (Clone MG2B-57, cat. no. 401202, 1:50 dilution; RRID: AB_2744505) was purchased from BioLegend. Fluorochrome-conjugated antibody specific for human CD34 (Clone 581, cat. no. 555822, 1:500 dilution; RRID: AB_396151) was purchased from BD Biosciences. Fluorochrome-conjugated antibodies specific for human ULBP-1 (Clone 170818, cat. no. FAB1380P, 1:50 dilution; RRID: AB_2687471) was purchased from R&D Systems. Fixable Viability Dye eFluor506 (e506, cat. no. 65-0866-14, 1:500 dilution) was purchased from Affymetrix eBioscience; mouse Fc Block (antimouse CD16/32, cat. no. 553142, 1:50 dilution; RRID: AB_394656) was purchased from BD Biosciences. HLA-A*02:01-NY-ESO-1 (157–165) tetramer and HLA-B*07:02–NY-ESO-1 (60–72) tetramer were prepared and provided by NIH Tetramer Core Facility at Emory University (RRID:SCR_026557). All flow cytometry staining was performed following standard protocols, as well as specific instructions provided by the manufacturer of a particular antibody. Stained cells were analyzed using a MACSQuant Analyzer 10 flow cytometer (Miltenyi Biotech), following the manufacturer's instructions. FlowJo software v.9 (BD Biosciences; RRID:SCR_008520) was used for data analysis.

Enzyme-linked immunosorbent cytokine assays (ELISA)

Supernatants from cell culture assays were collected to quantify cytokine levels, including human IFN-γ, TNF-α, and IL-15, as well as mouse IL-6 and SAA-3. For human IFN-γ and TNF-α detection, capture and biotinylated antibody pairs were purchased from BD

Biosciences, streptavidin–HRP conjugate was purchased from Invitrogen, human cytokine standards were purchansed from eBioscience, and tetramethylbenzidine (TMB) substrate was purchased from KPL. Human IL-15 levels were measured using the Human IL-15 Quantikine ELISA Kit (R&D Systems, cat. no. D1500), following the manufacturer's protocol. Mouse serum was collected to quantify mouse IL-6 and SAA-3. Mouse IL-6 was quantified using purified anti-mouse IL-6 (Clone MP5-20F3, cat. no. 504501, Biolegend) and biotin-conjugated anti-mouse IL-6 (Clone MP5-32C11, cat. no. 504601, Biolegend) antibodies. Mouse SAA-3 levels were measured using the Mouse SAA-3 ELISA Kit (MilliporeSigma, cat. no. EZMSAA3-12K), according to the manufacturer's instructions. Absorbance at 450 nm was measured using an Infinite M1000 microplate reader (Tecan).

Generation of allogeneic HSPC-engineered ESO-T (^{Allo}ESO-T) cells and their IL-15-armed derivatives (denoted as $^{allo/15}$ESO-T cells)

$^{Allo/15}$ESO-T cells were generated by differentiating gene-engineered cord-blood CD34$^+$ HSPCs in a five-stage *ex vivo* HSPC-derived T cell culture.[38] ^{Allo}ESO-T cells were differentiated from HSPCs engineered to overexpress a transgenic human ESO TCR (either 1G4 or 1E4 in this study), and ^{Allo15}ESO-T cells were differentiated from HSPCs engineered to overexpress a human transgenic ESO TCR together with the secreted form of human IL-15. At stage 0, 1×10^4 frozen-thawed human CD34$^+$ HSPCs were revived and cultured in 500 μL of X-VIVO 15 Serum-Free Hematopoietic Stem Cell Medium supplemented with 50 ng/mL Flt3L, 50 ng/mL SCF, 50 ng/mL TPO and 20 ng/mL IL-3 in non-tissue culture treated 24-well plate for 24 h, then transduced with Lenti/1G4-(IL15) or Lenti/1E4 viral vectors for another 24 h following an established protocol.[38] Briefly, concentrated lentivirus supernatant was mixed with 1:100 Poloxamer Synperonic F108 and 1:1,000 Prostaglandin E2 (PGE2; CAYMAN), and then gently added to the HSPCs culture.

At stage 1, gene-engineered HSPCs collected from stage 0 were cultured in the StemSpan SFEM II Medium supplemented with StemSpan Lymphoid Progenitor Expansion Supplement for 2 weeks. CELLSTAR 24-well Cell Culture Non-treated Multiwell Plates (VWR, cat. no. 82050-892) were used. The plates were coated with 500 μL per well StemSpan Lymphoid Differentiation Coating Material for 2 h at room temperature or alternatively, overnight at 4°C. Transduced CD34$^+$ HSPCs were suspended at 2×10^4 cells/mL and 500 μL of cell suspension was added into each precoated well. Twice per week, half of the medium from each well was removed and replaced with fresh medium.

At stage 2, cells collected from the stage 1 were cultured in the StemSpan SFEM II Medium supplemented with StemSpan T cell Progenitor Maturation Supplement for 1 week. Non-Treated Falcon Polystyrene six-well Microplates (Thermo Fisher Scientific, cat. no. 140675) were coated with 1 mL per well of StemSpan Lymphoid Differentiation Coating Material. The stage 1 cells were collected and resuspended at 1×10^5 cells/mL; 2 mL of cell suspension was added into each precoated well. Cells were passaged 1–2 times per week to maintain a cell density at 0.5–1 $\times 10^6$ cells/mL; fresh medium was added at every passage.

At stage 3, cells collected from the stage 2 were cultured in the StemSpan SFEM II Medium supplemented with StemSpan T cell Progenitor Maturation Supplement, CD3/CD28/CD2 T cell Activator and 20 ng/mL human recombinant IL-15 for 1 week. Cells were resuspended at 5×10^5 cells/mL; 2 mL cell suspension was added into Non-Treated Falcon Polystyrene six-well Microplates (Thermo Fisher Scientific) precoated with 1 mL per well of StemSpan Lymphoid Differentiation Coating Material. Cells were passaged 2–3 times per week to maintain a cell density at 0.5–1 $\times 10^6$ cells/mL; fresh medium was added at every passage.

At stage 4, cells collected from the stage 3, now mature $^{Allo/15}$ESO-T cells were expanded using various expansion approaches: (1) an αCD3/αCD28 expansion approach, (2) an NY-ESO-1 peptide-loaded PBMC expansion approach or (3) an aAPC expansion approach. The expansion stage lasted for 2 weeks. At stage 4, cells could be cultured in 150 mm cell culture dishes (Thermo Fisher Scientific) or G-Rex 6M Well Plates (Wilson Wolf). The expansion can happen in a feeder-free, serum-free CTS OpTmizer T cell Expansion SFM (Thermo Fisher Scientific) or a homemade C10 medium. The resulting $^{Allo/15}$ESO-T cells were aliquoted and cryopreserved in CryoStor Cell Cryopreservation Media CS10 using a Thermo Scientific CryoMed Controlled-Rate Freezer 7450 (Thermo Scientific) for future use, following the manufacturer's instructions.

(1) The αCD3/αCD28 antibody expansion approach used 150 mm cell culture dishes (Thermo Fisher Scientific), which were coated with 1 μg/mL (500 μL per well) of Ultra-LEAF Purified Anti-Human CD3 Antibody (Clone OKT3; BioLegend) for 2 h at room temperature or, alternatively, overnight at 4°C. Mature $^{Allo/15}$ESO-T cells collected from the stage 3 culture were resuspended in the expansion medium supplemented with 10 ng/mL IL-7, 10 ng/mL IL-15 and 1 μg/mL Ultra-LEAF Purified Anti-Human CD28 antibody (Clone CD28.2; BioLegend) at 5×10^5 cells/mL; 30 mL cell suspension was added into each plate. After 3 days of culture, cells were collect ed and resuspended in fresh expansion medium supplemented with 10 ng/mL IL-7 and IL-15, at 0.5–1 $\times 10^6$ cells/mL. Cells were passaged 2–3 times per week to maintain a cell density at 0.5–1 $\times 10^6$ cells/mL; fresh medium was added at every passage. As the cell population reaches a high number during the later stages, these cells can be transferred and cultured in G-Rex 6M Well Plates (Wilson Wolf).

(2) The NY-ESO-1 PBMC expansion approach. Healthy donor PBMCs were loaded with NY-ESO-1 peptide at 5 μM in C10 medium for 1 h. The resulting NY-ESO-1 peptide-loaded PBMCs (NY-ESO-1 PBMCs) were then irradiated at 6,000 rads using a Rad Source RS-2000 X-ray Irradiator (Rad Source Technologies). Mature $^{Allo/15}$ESO-T cells and derivatives collected from the stage 3 culture were mixed with the irradiated NY-ESO-1–PBMCs at a 1:5 ratio, resuspended in expansion medium supplemented with 10 ng/mL IL-7 and IL-15 at 0.5–1 $\times 10^6$ cells/mL and seeded into the 150 mm cell culture dishes (Thermo Fisher

Scientific) at 30 mL per plate. Cells were passaged 2–3 times per week to maintain a cell density at 0.5–1 × 10^6 cells/mL; fresh medium was added at every passage. As the cell population reaches a high number during the later stages, these cells can be transferred and cultured in G-Rex 6M Well Plates (Wilson Wolf).

(3) The aAPC expansion approach. aAPCs were irradiated at 10,000 rads using a Rad Source RS-2000 X-ray Irradiator (Rad Source Technologies). Mature $^{Allo/15}$ESO-T cells collected from the stage 3 culture were mixed with the irradiated aAPCs at a 1:1 ratio, resuspended in expansion medium supplemented with 10 ng/mL IL-7 and IL-15 at 0.5–1 × 10^6 cells/mL, and seeded into the 150 mm cell culture dishes (Thermo Fisher Scientific) at 30 mL per plate. Cells were passaged 2–3 times per week to maintain a cell density at 0.5–1 × 10^6 cells/mL; fresh medium was added at every passage. As the cell population reaches a high number, cells can be transferred to and cultured in G-Rex 6M Well Plates (Wilson Wolf).

Generation and analysis of allogeneic HSPC-engineered HLA-ablated universal T (^{U}ESO-T) cells

The generation and analysis of ^{U}ESO-T cells is shown in Figure S4. Briefly, CD34^{+} HSPCs were thawed, cultured, and transduced with the Lenti/1G4-HLAE-TK vector. One day post-transduction, CRISPR-Cas9 electroporation was performed to knock out the *B2M* and *CIITA* genes as previously described.[67,114] Single-guide RNAs targeting *B2M* (CGCGAGCACAGCUAAGGCCA) and *CIITA* (GAUAUUGGCAUAAGCCUCCC) were purchased from Synthego. Engineered cells were then subjected to a four-stage, six-week expansion and maturation protocol, as described in the previous section, to generate HLA-I/II-deficient ^{U}ESO-T cells. The phenotypes of ^{U}ESO-T cells were characterized by flow cytometry analysis.

Generation of PBMC-derived NK cells and PBMC-derived ESO-T (^{PBMC}ESO-T) cells

Healthy donor PBMCs were used to generate the PBMC-derived conventional ESO-T and NK cells (denoted as ^{PBMC}ESO-T and PBMC-NK cells, respectively). To generate PBMCESO-T cells, peripheral blood mononuclear cells (PBMCs) were first activated using Dynabeads Human T-Activator CD3/CD28 (Thermo Fisher Scientific) according to the manufacturer's protocol. Cells were then transduced with either Lenti/1G4 or Lenti/1E4 vectors for 24 h, followed by culture in C10 medium supplemented with 30 ng/mL IL-2 for 2–3 weeks. To generate PBMC-NK cells, PBMCs were sorted with MACS using a Human NK Cell Isolation Kit (Miltenyi Biotec), following the manufacturers' instructions.

In vitro tumor cell killing assay

FG-labeled tumor cells (1 × 10^4 cells per well) were co-cultured with therapeutic cells at different effector-to-target (E:T) ratios specified in the figures. For ^{PBMC}ESO-T cell products, NY-ESO-1 tetramer^{+} cells were purified by tetramer-based sorting prior to co-culture. Cultures were set up in Corning 96-well clear-bottom black plates using C10 medium and incubated for 24 h. To quantify viable tumor cells, D-luciferin (150 μg/mL; Caliper Life Sciences) was added directly to the wells, and luminescence was measured at 560 nm using an Infinite M1000 microplate reader (Tecan).

Additional tumor killing experiments were conducted using A375-FG tumor cells co-cultured with ^{Allo15}ESO-T cells. To study the NK activating receptor-mediated tumor cell killing mechanism, 10 μg/mL of Ultra-LEAF purified anti-human NKG2D (clone 1D11, BioLegend), anti-human DNAM-1 (clone 11A8, BioLegend), or Ultra-LEAF purified mouse IgG2b, κ isotype control (clone MG2B-57, BioLegend) were added to co-cultures. For serial tumor killing assays, unlabeled stimulator tumor cells were added every 2 days over a total duration of 20 days. FG-labeled indicator cells were introduced 24 h before each killing readout to assess cytotoxic activity. Tumor killing was measured every 2 days using the same luciferase-based luminescence assay.

In vitro mixed lymphocyte reaction (MLR) for studying GvH response

HLA-A2^{+} or HLA-A2^{-} PBMCs of multiple random healthy donors were irradiated at 3,000 rads and used as stimulators to study the GvH response of ^{Allo15}ESO-T cells as responders. ^{PBMC}ESO-T cells were included as a responder control. Stimulators (5 × 10^5 cells per well) and responders (2 × 10^4 cells per well) were cocultured in 96-well round-bottom plates in C10 medium for 4 days; the cell culture supernatants were then collected to measure IFN-γ production using ELISA.

In vitro MLR assay for studying T cell-mediated allorejection

PBMCs of multiple healthy donors were used as responders to study the T cell-mediated allorejection of ^{Allo15}ESO-T cells as stimulators (irradiated at 3,000 rads). ^{PBMC}ESO-T cells were included as a stimulator control. Stimulators (5 × 10^5 cells per well) and responders (2 × 10^4 cells per well) were cocultured in 96-well round-bottom plates in C10 medium for 4 days; the cell culture supernatants were then collected to measure IFN-γ production using ELISA.

In vitro MLR assay for studying NK cell-mediated allorejection

PBMC-derived NK cells obtained from multiple healthy donors were employed to investigate the NK cell-mediated allorejection of ^{Allo15}ESO-T cells. ^{PBMC}ESO-T cells were included as an allogeneic subject control. PBMC-NK cells (2 × 10^4 cells per well) and the corresponding allogeneic subject cells (2 × 10^4 cells per well) were cocultured in 96-well round-bottom plates with C10 medium for 24 h. Subsequently, the cell cultures were collected to quantify live cells via flow cytometry.

In vitro ganciclovir (GCV) treatment assay

^{U}ESO-T cells were cultured *in vitro* with GCV of gradient concentrations (specified in figure legends) in C10 medium for 3 days. Live cells were quantified via FACS analysis.

In vivo bioluminescence imaging (BLI)

BLI was conducted using the Spectral Advanced Molecular Imaging (AMI) HTX system (Spectral Instrument Imaging). Live animal images were acquired 5 min after intraperitoneal (i.p.) injection of D-luciferin to capture whole-body bioluminescence signals. To monitor tumor cells, 1 mg of D-luciferin was administered per mouse. To monitor therapeutic cells, 3 mg of D-luciferin was administered per mouse. Imaging data were processed and analyzed using AURA imaging software (Spectral Instrument Imaging, version 3.2.0).

In vivo antitumor efficacy study of ^{Allo15}ESO-T cells: Orthotopic human OVCAR3 xenograft NSG mouse model

Experimental design is showed in Figure 3A. On Day 0, NSG mice received i.p. inoculation of OVCAR3-FG human ovarian cancer cells (1×10^6 cells per mouse). On day 7, the experimental mice received intravenous (i.v.) injection of vehicle (100 μL PBS per mouse), ^{Allo15}ESO-T cells (10×10^6 cells in 100 μL PBS per mouse), or control ^{PBMC}ESO-T cells (10×10^6 cells in 100 μL PBS per mouse). Over the experiment, mice were monitored for survival and their tumor loads were measured using BLI.

In vivo antitumor efficacy study of ^{Allo15}ESO-T cells: Metastatic human A375 xenograft NSG mouse model

Experimental design is showed in Figure 4A. On Day 0, NSG mice received i.v. inoculation of A375-FG human melanoma cells (2×10^5 cells per mouse). On day 3, the experimental mice received i.v. injection of vehicle (100 μL PBS per mouse), ^{Allo15}ESO-T cells (10×10^6 cells in 100 μL PBS per mouse), or control ^{PBMC}ESO-T cells (10×10^6 cells in 100 μL PBS per mouse). Over the experiment, mice were monitored for survival and their tumor loads were measured using BLI.

In vivo PK-PD study of ^{Allo15}ESO-T/FG cells: Orthotopic human OVCAR3 xenograft NSG mouse model

Experimental design is shown in Figure 3F. On day 0, NSG mice received i.p. inoculation of OVCAR3 cells (1×10^6 cells per mouse). On day 7, the OVCAR3-bearing mice or tumor-free mice received i.v. injection of ^{Allo15}ESO-T/FG cells (10×10^6 cells in 100 μL of PBS per mouse) or control ^{PBMC}ESO-T/FG cells (10×10^6 cells in 100 μL of PBS per mouse). Over the experiment, mice were monitored for survival and their therapeutic cells were measured using BLI. Note in this study, therapeutic immune cells, but not the tumor cells, were labeled with FG.

In vivo PK-PD study of ^{Allo15}ESO-T/FG cells: Metastatic human A375 xenograft NSG mouse model

Experimental design is shown in Figure 4F. On day 0, NSG mice received i.v. inoculation of A375 cells (2×10^5 cells per mouse). On day 3, the A375-bearing mice received i.v. injection of ^{Allo15}ESO-T/FG cells (10×10^6 cells in 100 μL of PBS per mouse) or control ^{PBMC}ESO-T/FG cells (10×10^6 cells in 100 μL of PBS per mouse). Over the experiment, mice were monitored for survival and their therapeutic cells were measured using BLI.

In vivo NKG2A immune checkpoint blockade study of ^{Allo15}ESO-T cells: Heavy tumor load human A375 xenograft NSG mouse model

Experimental design is shown in Figure 6G. On Day 0, NSG mice were intravenously inoculated with A375-FG cells (2×10^5 cells per mouse). On Day 7, mice received one of the following intravenous treatments: vehicle control (100 μL PBS per mouse), ^{Allo15}ESO-T cells (5×10^6 cells in 100 μL PBS per mouse), anti-NKG2A antibody (Monalizumab; MCE, cat. no. HY-P99032;10 mg/kg), or a combination of ^{Allo15}ESO-T cells (5×10^6 cells in 100 μL PBS per mouse) with anti-NKG2A antibody (10 mg/kg). For groups receiving anti-NKG2A treatment, the antibody was administered every 7 days throughout the duration of the experiment. Mice were monitored for survival, and tumor burden was assessed using BLI.

In vivo CRS study

Experimental design is shown in Figure 7N. On day 0, NSG mice received i.p. inoculation of A375-FG cells (5×10^6 cells per mouse). On day 7, the OVCAR3-bearing mice received i.p. injection of ^{Allo15}ESO-T cells (10×10^6 cells in 100 μL of PBS per mouse) or control ^{PBMC}ESO-T cells (10×10^6 cells in 100 μL of PBS per mouse). On days 8 and 10, blood samples were collected from the experimental mice, and their serum IL-6 and SAA-3 were measured using ELISA.

Single-cell TCR sequencing

^{PBMC}ESO-T, ^{Allo}ESO-T, and ^{Allo15}ESO-T cells were sorted using a FACSAria III sorter (BD Biosciences) and immediately submitted to the UCLA Technology Center for Genomics and Bioinformatics (TCGB) Core for single-cell TCR sequencing. Sequencing was performed using the 10X Genomics Chromium Controller Single Cell System, following the manufacturer's instruction and TCGB Core's standard protocols. Libraries were constructed with the Illumina TruSeq RNA Sample Prep Kit and sequenced on an Illumina

NovaSeq 6000 using 150 bp paired-end reads at a depth of 5,000 reads per cell. Sequencing reads were aligned to the human TCR reference genome (hg38) using Cell Ranger VDJ (10X Genomics), and the frequencies of TCR α and β chain recombination were analyzed and visualized.

Single-cell RNA sequencing (scRNA-seq)

scRNA-seq was performed to profile *in vivo* gene expression in ^{Allo15}ESO-T cells before and after infusion into A375 tumor-bearing NSG mice. The experimental timeline is illustrated in Figure 5A. On day −3, A375 cells were injected intravenously into NSG mice. On day 0, mice received intravenous injections of ^{Allo15}ESO-T cells or control ^{PBMC}ESO-T cells. Pre-infusion samples were collected prior to cell infusion. On day 21, therapeutic cells were isolated from lung tissue and peripheral blood, pooled from 10 mice in each experimental group, and used as post-infusion samples for scRNA-seq. Freshly collected samples were immediately delivered to the UCLA TCGB Core for library construction and scRNA-seq.

Cells were quantified using a Cell Countess II automated cell counter (Invitrogen/Thermo Fisher Scientific). A total of 10,000 cells from each experimental group were loaded on the Chromium platform (10X Genomics), and libraries were constructed using the Chromium Next GEM Single Cell 3′ Kit v3.1 and the Chromium Next GEM Chip G Single Cell Kit (10X Genomics), according to the manufacturer's instructions. Library quality was assessed using the D1000 ScreenTape on a 4200 TapeStation System (Agilent Technologies). Libraries were sequenced on an Illumina NovaSeq using the NovaSeq S4 Reagent Kit (100 cycles; Illumina). For cell clustering and annotation, the merged digital expression matrix generated by Cell Ranger was analyzed using an R package Seurat (v.4.0.0) following the guidelines. Briefly, after filtering the low-quality cells, the expression matrix was normalized using NormalizeData function, followed by selecting variable features across datasets using FindVariableFeatures and SelectIntegrationFeatures functions. To correct the batch effect, FindIntegrrationAnchors and IntegrateData functions were used based on the selected feature genes. The corrected dataset was subjected to standard Seurat workflow for dimension reduction and clustering. In this study, clusters of therapeutic cells were manually merged and annotated based on gene signatures reported from Human Protein Atlas (proteinatlas.org) and previous studies.[38,68,70] AddModuleScore was used to calculate module scores of each list of gene signatures, and FeaturePlot function was used to visualize the expression of each signature in the UMAP plots.

Histology

Liver and lung tissues were collected on day 35 from A375-inoculated NSG mice treated with ^{Allo15}ESO-T cells, ^{PBMC}ESO-T cells, or left untreated (blank control). Liver, lung, heart, spleen, kidney and brain tissues were collected on day 55 from OVCAR3-inoculated NSG mice treated with ^{Allo15}ESO-T cells. Tissues were fixed in 10% Neutral Buffered Formalin for up to 36 h, then embedded in paraffin and sectioned at 5 μm thickness. Hematoxylin and eosin (H&E) and Immunohistology (IHC) staining was performed by the UCLA Translational Pathology Core Laboratory according to standard protocols. Stained sections were imaged using an Olympus BX51 upright microscope equipped with an Optronics Macrofire CCD camera (AU Optronics), and image analysis was conducted using Optronics PictureFrame software (AU Optronics). Additionally, tissues were analyzed for inflammation (Inf), hematopoietic neoplasm (HN), and nonhematopoietic neoplasm (NHN). Data were presented as pathologist's scores of individual mouse tissues ($n = 5$). 0, no abnormal findings; 1, mild; 2, moderate; 3, severe.

QUANTIFICATION AND STATISTICAL ANALYSIS

Graphpad Prism v.8 software (Graphpad; RRID:SCR_002798) was used for statistical data analysis. Student's two-tailed *t* test was used for pairwise comparisons. Ordinary one-way analysis of variance (ANOVA) followed by Tukey's or Dunnett's multiple comparisons test was used for multiple comparisons. A log rank (Mantel-Cox) test adjusted for multiple comparisons was used for Meier survival curves analysis. Data are presented as the mean ± s.e.m., unless otherwise indicated. In all figures and figure legends, n represents the number of samples or animals used in the indicated experiments. Statistical significance was defined as follows: ns, not significant ($p \geq 0.05$); $p < 0.05$ (*); $p < 0.01$ (**); $p < 0.001$ (***); and $p < 0.0001$ (****). The statistical test used for each experiment is indicated in the corresponding figure legend.

Cell Reports Medicine, Volume *7*

Supplemental information

Scalable generation of hematopoietic stem cell-engineered off-the-shelf mono-specific cytotoxic T cells targeting solid tumors

Yichen Zhu, Jiaji Yu, Yu Jeong Kim, Yanxin Tian, Zhe Li, Yuning Chen, Zibai Lyu, Enbo Zhu, Annabel S. Zhao, Nathan Ma, Catherine Zhang, Adam Kramer, Matthew Wilson, Ryan Hon, Yu-Chen Wang, Siyu Lin, Xinyuan Shen, Zoe Hahn, Yuchong Zhang, Aijun Wang, Yan-Ruide Li, and Lili Yang

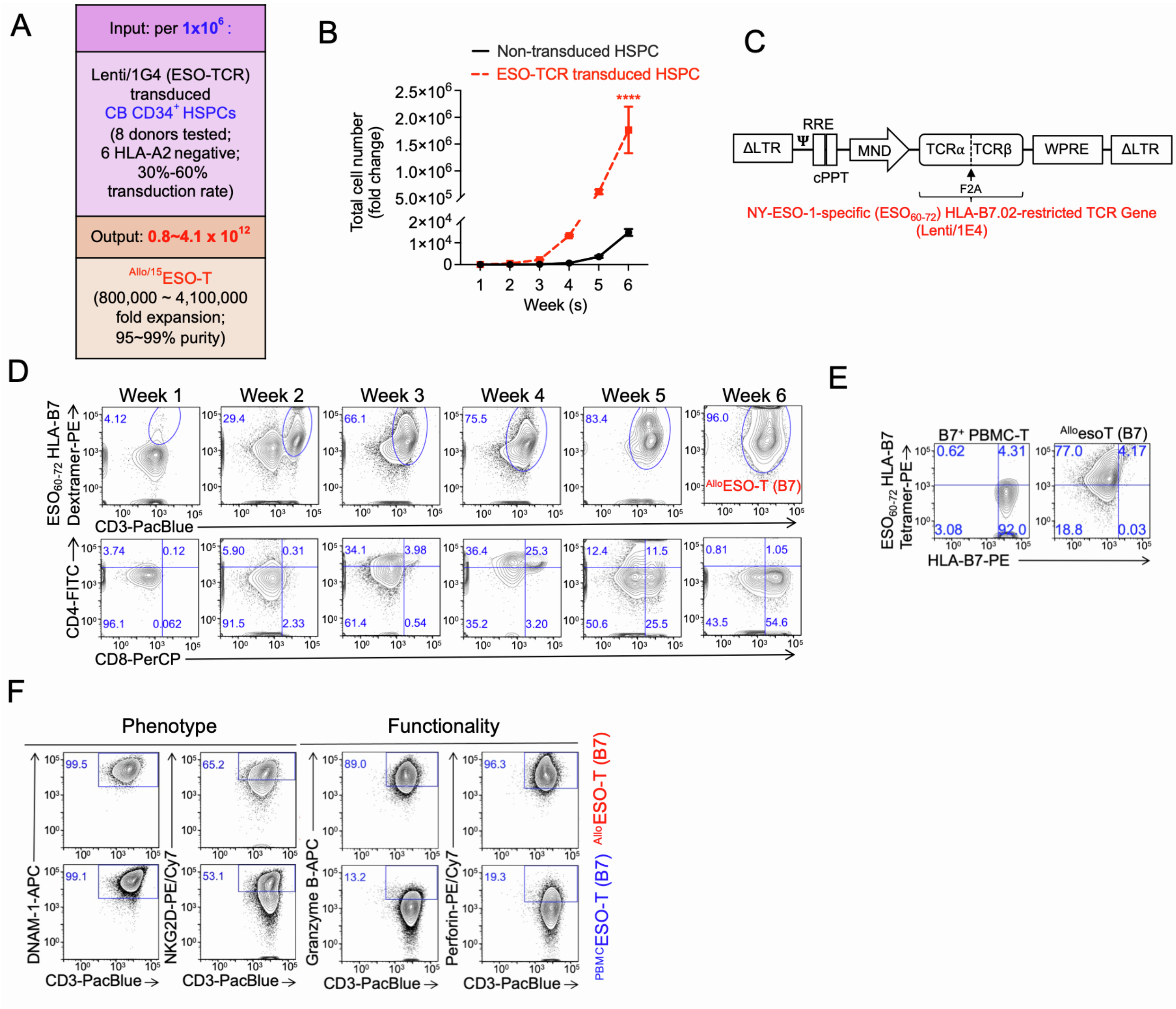

Figure S1: Generation and characterization of allogeneic NY-ESO-1 targeting mono-specific cytotoxic T (^{Allo}ESO-T) cells; related to Figure 1.

(A) Yield of $^{Allo/15}$ESO-T cells from 8 different CB donors.

(B) Growth curve of HSPCs either non-transduced or transduced with exogenous ESO-TCR in the culture (n = 3).

(C) Schematics showing the design of the lentiviral vector carrying HLA-B7.02 restricted, NY-ESO-1$_{60-72}$-specifc TCR.

(D) FACS monitoring of the generation of ^{Allo}ESO-T (B7) cells (identified as ESO$_{60-72}$ HLA-B7 Dextramer$^+$CD3$^+$ cells) over the 6-week *ex vivo* HSPC-ESO-T cell culture.

(E) FACS analyses showing the generation of ^{Allo}ESO-T (B7) is independent of matching MHC expression for positive selection.

(F) FACS detection of surface markers and cytotoxic molecules of ^{Allo}ESO-T (B7) cells.

Representative of 3 independent biological replicates. Data are presented as the mean ± SEM. ns, not significant, *p<0.05, ****p<0.0001 by Student's *t* test (B).

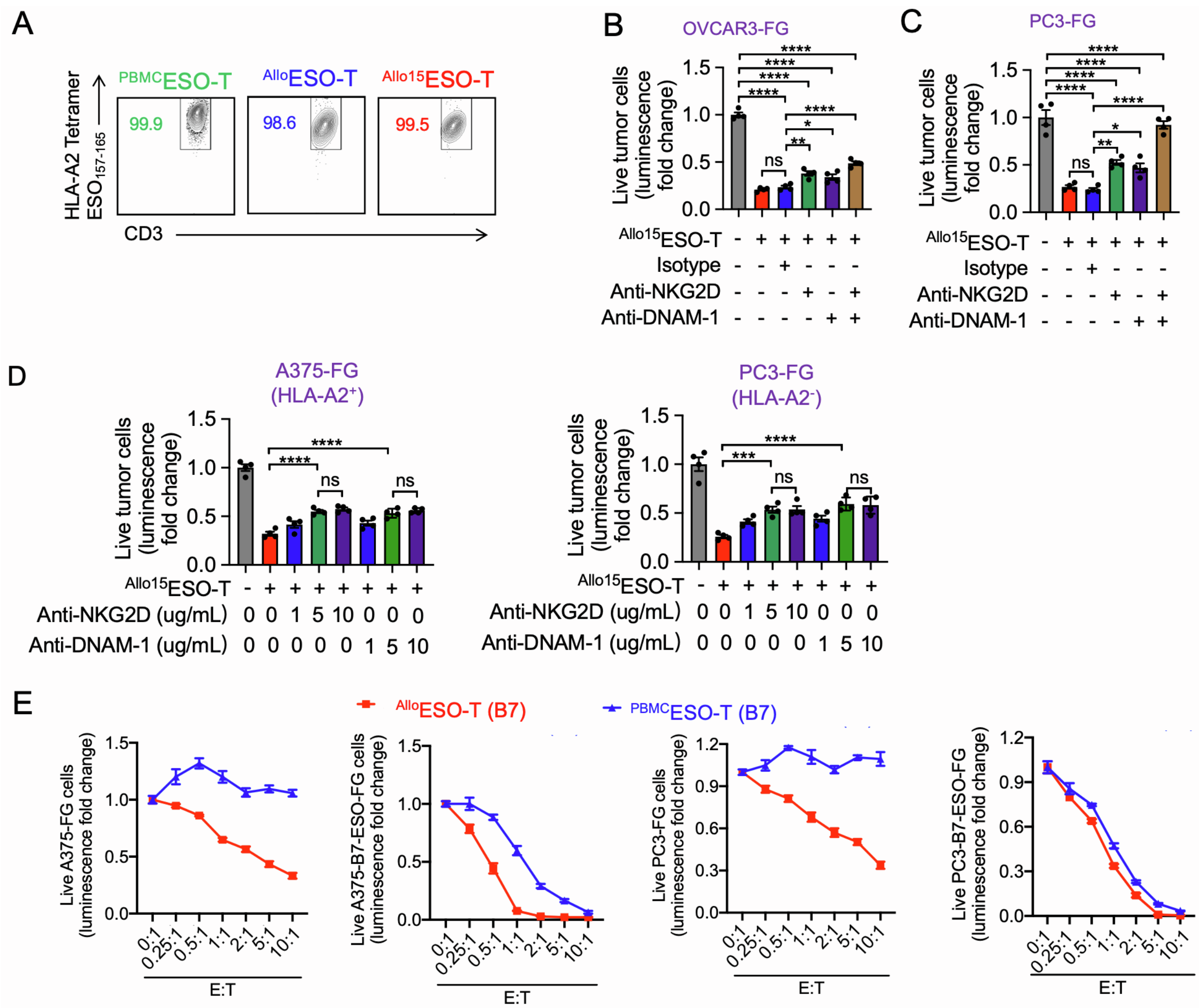

Figure S2: *In vitro* antitumor efficacy and mechanism of action (MOA) study of Allo/15ESO-T cells; related to Figure 2.

(A) NY-ESO-1 tetramer staining of Allo15ESO-T, AlloESO-T, and PBMCESO-T cell products.

(B-D) Studying the tumor cell killing mechanisms of Allo15ESO-T cells mediated by NKG2D and DNAM-1. (B) OVCAR3-FG tumor cell killing data at 24 h (E:T ratio = 0.5:1; n = 4). (C) PC3-FG tumor cell killing data at 24 h (E:T ratio = 5:1; n = 4). (D) Dose–response titration study of NK receptor–blocking antibodies. Tumor cell killing at 24 h at indicated concentrations.

(E) *In vitro* tumor cell killing data at 24 h by AlloESO-T (B7) (n = 4). PBMCESO-T (B7) cells were included as a therapeutic cell control.

Representative of 3 experiments. Data are presented as the mean ± SEM. ns, not significant, *p<0.05, **p<0.01, ***p<0.001, ****p<0.0001 by one-way ANOVA (A-D).

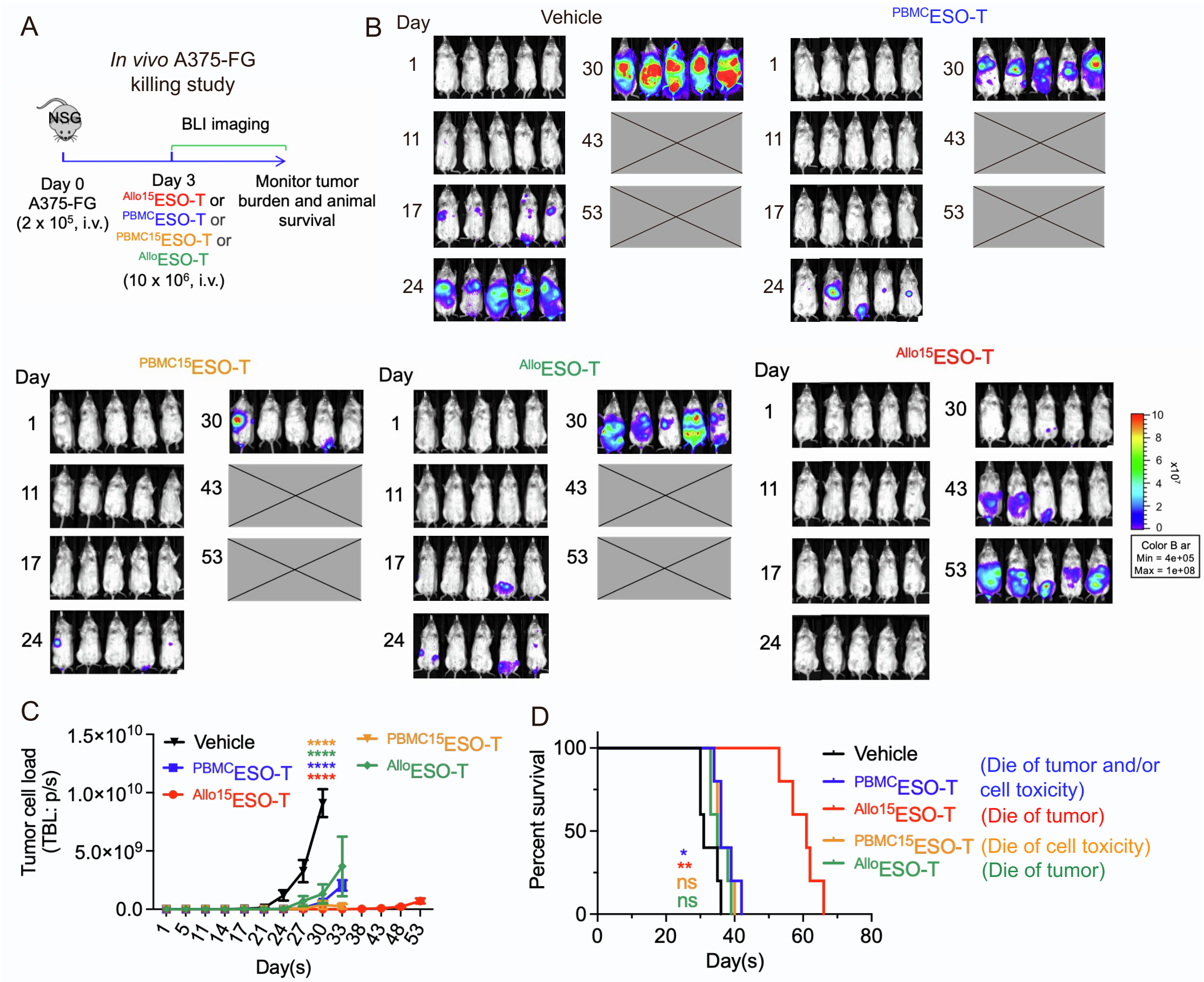

Figure S3: *In vivo* antitumor efficacy study of $^{Allo/15}$ESO-T cells in a metastatic melanoma tumor model. related to Figure 4.

(A) Experimental design. BLI, live animal bioluminescence imaging. (B) BLI images measuring tumor loads in experimental mice over time. (C) Quantification of c (n = 5). (D) Kaplan–Meier survival curves (n = 5).

Note that the data for the Vehicle, ^{Allo15}ESO-T, and ^{PBMC}ESO-T groups were also presented in the main Figs. 4A-4E.

Representative of 2 independent biological replicates. Data are presented as the mean ± SEM. ****p<0.0001 by one-way ANOVA (C)

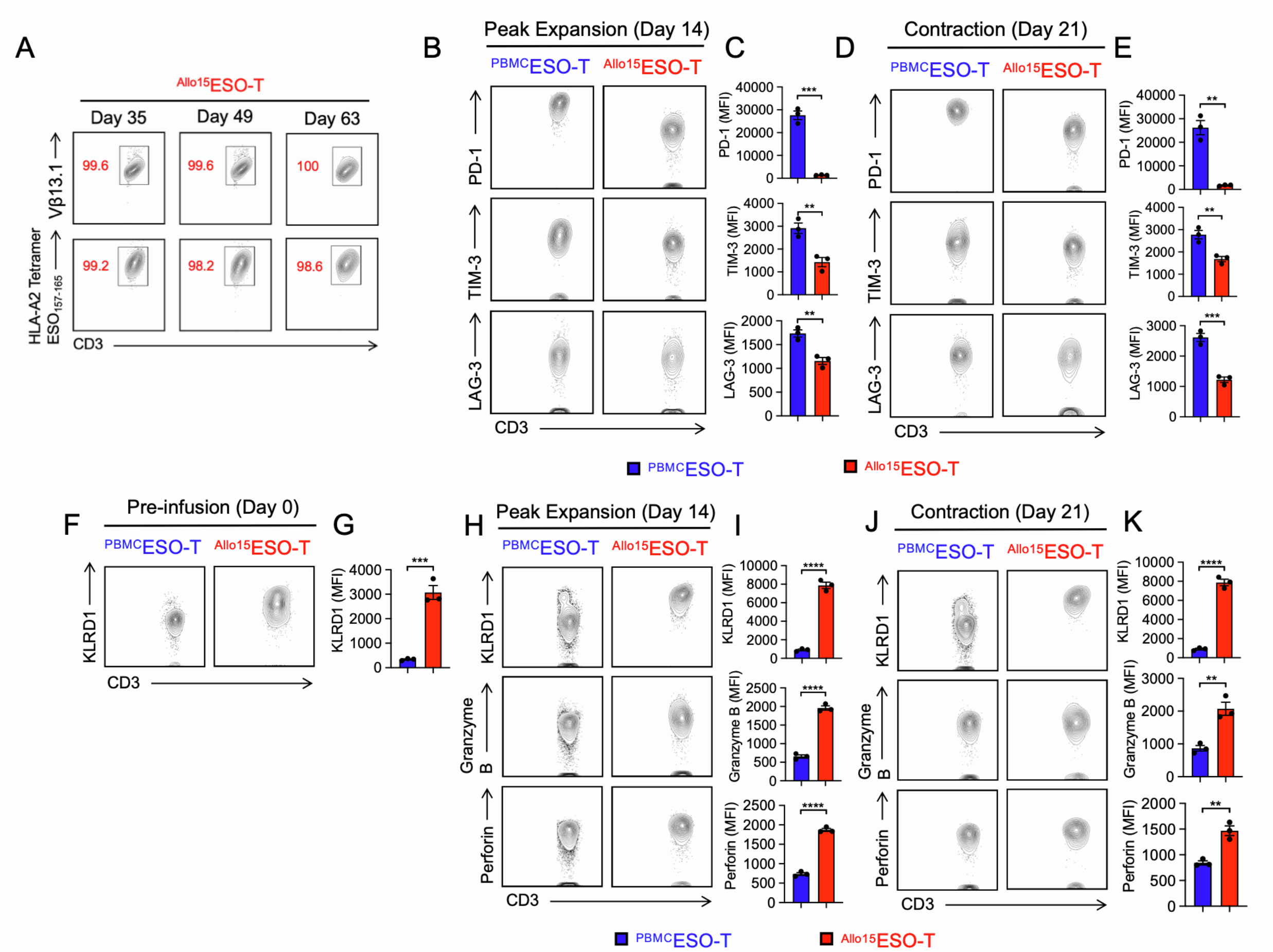

Figure S4: *In vivo* profiling of ^{Allo15}ESO-T cells in a metastatic melanoma tumor model. related to Figures 3 and 5.

(A) Transgenic TCR and NY-ESO-1 tetramer staining of ^{Allo15}ESO-T cells *in vivo* at multiple time points.

(B, D, F, H, and J) FACS detection of TEMRA and exhaustion marker expression *in vivo*. (C, E, G, I, and K) Quantification of (B, D, F, H, and J) (n = 3).

Representative of 3 independent biological replicates. Data are presented as the mean ± SEM. **p<0.01, ***p<0.001, ****p<0.0001 by Student's *t* test (C, E, G, I, and K)

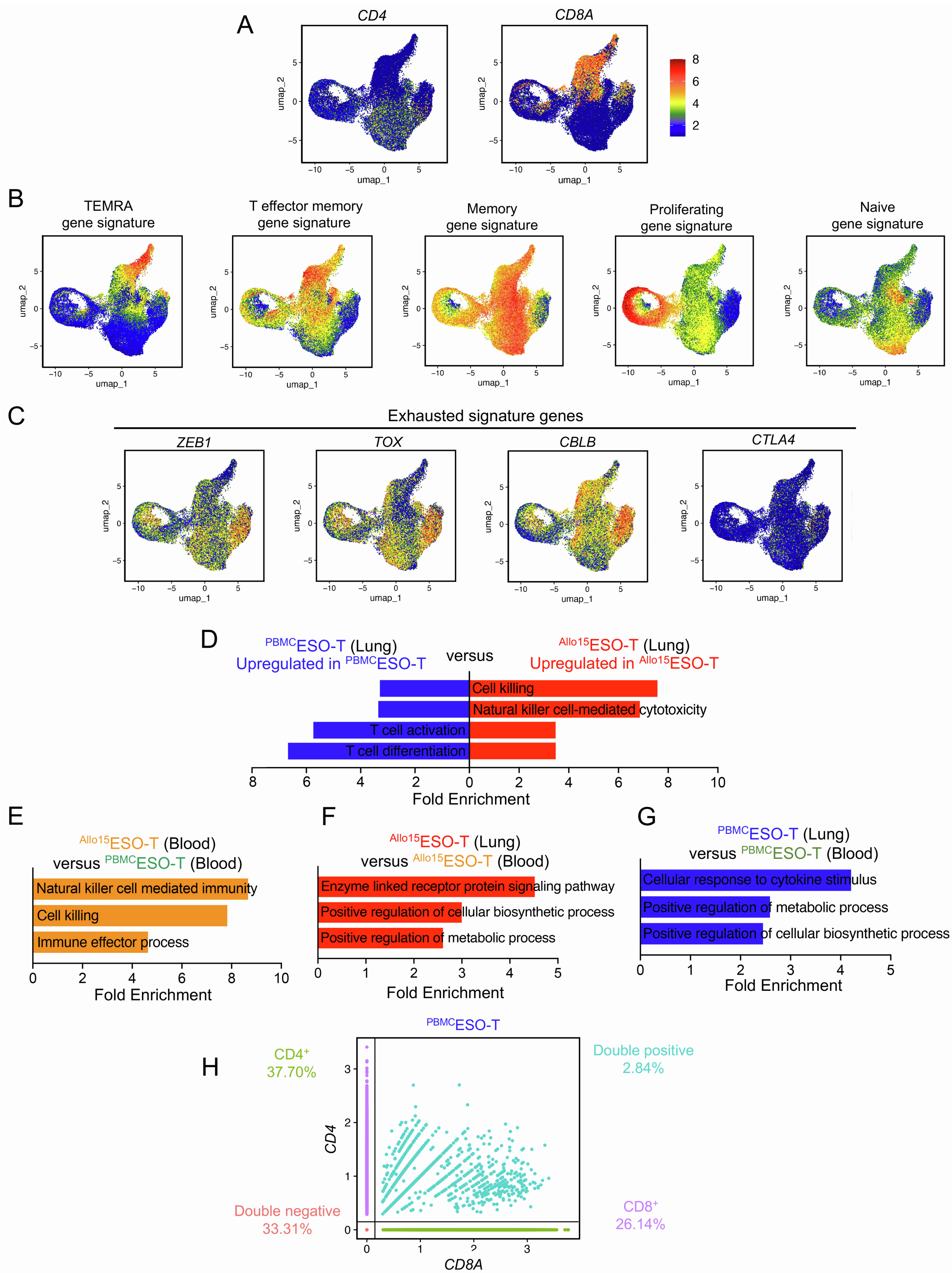

Figure S5: *In vivo* gene profiling study of Allo15ESO-T cells; related to Figure 5.
(A) UMAP plots showing the expression of *CD4* and *CD8A*.

(B) UMAP plots showing the expression distribution of TEMRA, T effector memory, memory, proliferating, naïve gene signatures.
(C) UMAP plots showing the expression of the exhausted signature genes *ZEB1*, *TOX*, *CBLB* and *CTLA4*.
(D-E) Pathway analyses of differentially expressed genes comparing lung-derived (D) and periphery blood-derived (E) ^{Allo15}ESO-T cells with ^{PBMC}ESO-T cells.
(F-G) Pathway analysis of differentially expressed genes comparing lung- versus blood-derived ^{Allo15}ESO-T cells (f) and lung- versus blood-derived ^{PBMC}ESO-T cells (G).
(H) Scatter plot showing single-cell expression levels of *CD4* (y-axis) and *CD8A* (x-axis) in ^{PBMC}ESO-T cells. Cells were classified into four populations: $CD4^+$ (green, 37.70%), $CD8^+$ (purple, 26.14%), double positive (cyan, 2.84%), and double negative (red, 33.31%), based on thresholded expression of *CD4* and *CD8A* transcripts.

The experiment was performed once; cells isolated from five mice of each experimental group were combined for analyses.

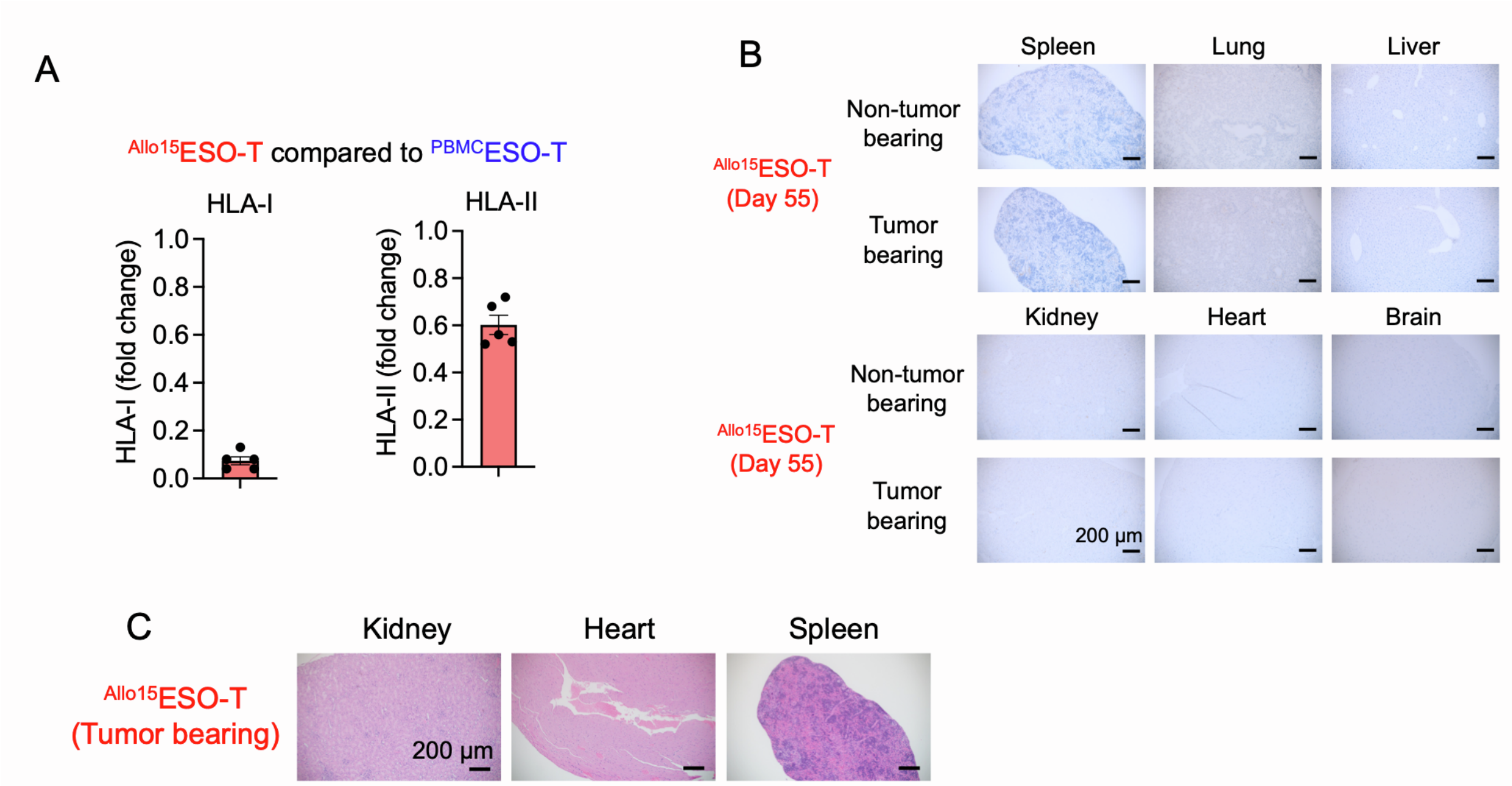

D

Group	Mouse ID	Pathologist's score																										
		Bone marrow			Spleen			Ileum			Pancreas			Kidney			Lung			Liver			Heart			Brain		
		Inf	HN	NHN	Inf	HN	NHN	Inf	HN	NHN	Inf	HN	NHN	Inf	HN	NHN	Inf	HN	NHN	Inf	HN	NHN	Inf	HN	NHN	Inf	HN	NHN
Allo15ESO-T	1	0	0	0	0	0	0	1	0	0	0	0	0	0	0	0	0	0	0	0	0	0	0	0	0	0	0	0
	2	0	0	0	0	0	0	0	0	0	0	0	0	0	0	0	0	0	0	0	0	0	0	0	0	0	0	0
	3	0	0	0	1	0	0	0	0	0	0	0	0	0	0	0	0	0	0	1	0	0	0	0	0	0	0	0
	4	0	0	0	0	0	0	0	0	0	0	0	0	0	0	0	1	0	0	0	0	0	0	0	0	0	0	0
	5	0	0	0	0	0	0	0	0	0	0	0	0	0	0	0	0	0	0	0	0	0	0	0	0	0	0	0
Blank NSG	6	0	0	0	0	0	0	0	0	0	0	0	0	0	0	0	0	0	0	0	0	0	0	0	0	0	0	0
	7	0	0	0	0	0	0	0	0	0	0	0	0	0	0	0	0	0	0	0	0	0	0	0	0	0	0	0
	8	0	0	0	0	0	0	0	0	0	0	0	0	0	0	0	0	0	0	0	0	0	0	0	0	0	0	0
	9	0	0	0	0	0	0	1	0	0	0	0	0	0	0	0	0	0	0	0	0	0	0	0	0	0	0	0
	10	0	0	0	0	0	0	0	0	0	0	0	0	0	0	0	0	0	0	1	0	0	0	0	0	0	0	0

Pathologist's score: 0 No abnormal findings; 1 Mild; 2 Moderate; 3 Severe

LEGEND: Inf: Inflammation; HN: Hematopoietic Neoplasm; NHN: Non-Hematopoietic Neoplasm

Figure S6. Safety and immunogenicity study of ^{Allo15}ESO-T cells; related to Figure 7.
(A) Fold change of HLA-I/II expression between ^{Allo15}ESO-T cells and ^{PBMC}ESO-T cells.
(B) Immunohistochemical staining of human CD3 in major organs at day 55 post-infusion. Scale bar, 200 μm.
(C) Hematoxylin and eosin-stained tissue sections. Tissues were collected on day 55.
(D) Studying the long-term safety of ^{Allo15}ESO-T cells. Tissues from experimental mice were collected 55 days after injection with ^{Allo15}ESO-T cells. Data were presented as pathologist's scores of individual mouse tissues (n = 5).

Representative of 3 biological replicates. Data are presented as the mean ± SEM. ns, not significant by one-way ANOVA (B).

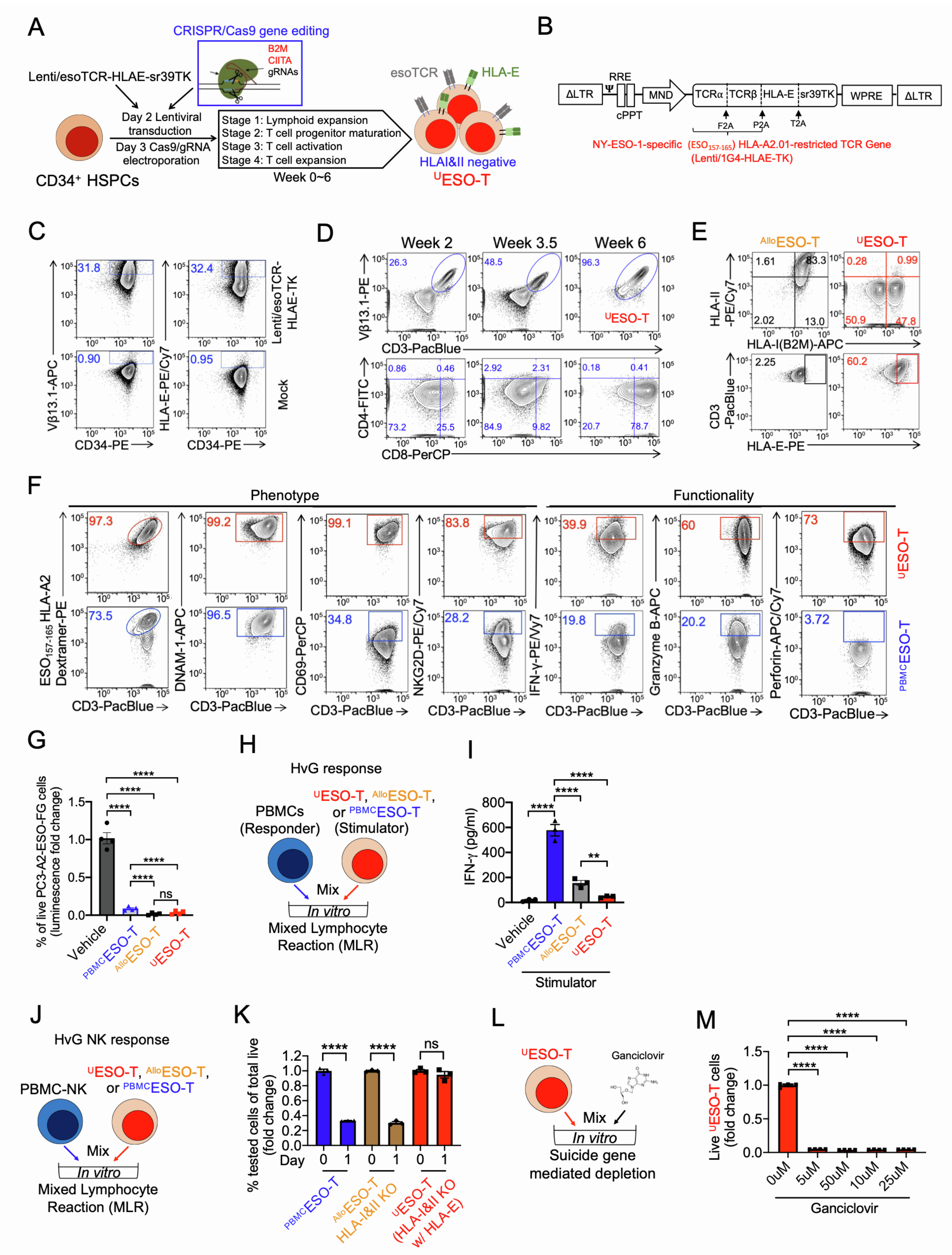

Figure S7: Development of allogeneic HLA-ablated universal T (^{U}ESO-T) cells.

(A) Schematics showing the generation of ^{U}ESO-T cells.
(B) Schematics showing the design of design of the lentiviral vector encoding a pair of ESO TCR α and β chains, an HLA-E, and a sr39TK suicide gene.
(C) Intracellular expression of ESO TCR and HLA-E in CD34^{+} HSPCs at 72 h after lentivector transduction.
(D) FACS monitoring of the generation of ^{U}ESO-T cells over the 6-week *ex vivo* HSPC-ESO-T cell culture.
(E) FACS detection of HLA-I/II and HLA-E expressions in ^{U}ESO-T cells.
(F) FACS detection of surface markers, intracellular cytokines and cytotoxic molecules in ^{U}ESO-T cells. ^{Allo}ESO-T cells were included for comparison.
(G) Tumor cell killing data by ^{U}ESO-T cells at 24 h.
(H-I) Studying the T cell-mediated allorejection of ^{U}ESO-T cells. (H) Experimental design. (I) ELISA analyses of IFNγ production on day 4 ($n = 4$).
(J-K) Studying the NK cell-mediated allorejection of ^{U}ESO-T cells. (J) Experimental design. (K) FACS analyses of the indicated live cells ($n = 3$).
(L-M) Studying the functional activity of the suicide gene sr39TK as an additional safety mechanism in ^{U}ESO-T cells. (L) Experimental design. ^{U}ESO-T cells were cultured in the presence of gradient concentrations of ganciclovir for 3 days. (M) FACS analyses of the indicated live cells on day 3 (n=4).

Representative of 3 independent biological replicates. Data are presented as the mean ± SEM. ns, not significant, $^{**}p < 0.01$, $^{****}p < 0.0001$, by one-way ANOVA (G, I, K, M).

Table S1. Cord blood donor information; related to Figure 1.

CB donors	Gender	Race	ABO/Rh
1	Male	Caucasian	O-
2	Female	Caucasian	A+
3	Female	Hispanic	O-
4	Male	Black	A-
5	Female	Hispanic	A+
6	Male	Asian	B+
7	Male	Caucasian	B-
8	Female	Unknown	O+
